# Exploring Ugi-Azide Four-Component Reaction Products for Broad-Spectrum Influenza Antivirals with a High Genetic Barrier to Drug Resistance

**DOI:** 10.1038/s41598-018-22875-9

**Published:** 2018-03-15

**Authors:** Jiantao Zhang, Yanmei Hu, Christopher Foley, Yuanxiang Wang, Rami Musharrafieh, Shuting Xu, Yongtao Zhang, Chunlong Ma, Christopher Hulme, Jun Wang

**Affiliations:** 1 Department of Pharmacology and Toxicology, College of Pharmacy, The University of Arizona, Tucson, Arizona 85721 United States; 2 Department of Chemistry and Biochemistry, The University of Arizona, Tucson, Arizona 85721 United States; 3 BIO5 Institute, The University of Arizona, Tucson, Arizona 85721 United States

## Abstract

Influenza viruses are respiratory pathogens that are responsible for seasonal influenza and sporadic influenza pandemic. The therapeutic efficacy of current influenza vaccines and small molecule antiviral drugs is limited due to the emergence of multidrug-resistant influenza viruses. In response to the urgent need for the next generation of influenza antivirals, we utilized a fast-track drug discovery platform by exploring multi-component reaction products for antiviral drug candidates. Specifically, molecular docking was applied to screen a small molecule library derived from the Ugi-azide four-component reaction methodology for inhibitors that target the influenza polymerase PA_C_-PB1_N_ interactions. One hit compound **5** was confirmed to inhibit PA_C_-PB1_N_ interactions in an ELISA assay and had potent antiviral activity in an antiviral plaque assay. Subsequent structure-activity relationship studies led to the discovery of compound **12a**, which had broad-spectrum antiviral activity and a higher *in vitro* genetic barrier to drug resistance than oseltamivir. Overall, the discovery of compound **12a** as a broad-spectrum influenza antiviral with a high *in vitro* genetic barrier to drug resistance is significant, as it offers a second line of defense to combat the next influenza epidemics and pandemics if vaccines and oseltamivir fail to confine the disease outbreak.

## Introduction

Influenza virus infection is responsible for both seasonal influenza as well as sporadic influenza pandemics^1^. In the annual influenza season, an estimated 10–20% of the human population is infected with the influenza virus. Despite the availability of influenza vaccines and small molecule antiviral drugs, the death toll of influenza virus-related illness surpasses that of breast cancer, which places the influenza virus among the top ten leading causes of death in the United States^2^. Moreover, the convenient transmission through airways, coupled with the high mortality rates associated with pandemic influenza viruses and highly pathogenic avian influenza (HPAI) viruses, renders the influenza virus a major public health concern^3^. For example, the CDC estimated the 2009 H1N1 influenza pandemic led to 284,000 deaths globally in the first 12 months of outbreak^4^. Over 400 cases of human infection by HPAI H7N9 were reported in the recent 2017 outbreak in China and the mortality rate was ~40%^5,6^. Therefore, next-generation vaccines and antiviral drugs are clearly needed with improved efficacy and antiviral spectrum to combat influenza virus infection.

Influenza vaccines remain the mainstay for the prophylaxis of influenza infection. They are normally effective in preventing seasonal influenza virus infection with an overall effectiveness of ~60%^7^. However, there is often a six-month delay from strain identification to batch production, which impedes its use at the beginning of an influenza outbreak^8^. Thus, small molecule antivirals are highly desired. They are not alternatives, but essential complements of influenza vaccines.

There are currently two classes of FDA-approved small molecule influenza antivirals: M2 channel blockers such as amantadine and rimantadine that inhibit the early stage of viral uncoating^9^, and neuraminidase inhibitors, such as oseltamivir, peramivir, and zanamivir that inhibit the last stage of viral egress^10^. The increasing incidences of drug-resistant viruses now call for the development of next generation influenza antivirals^11^. Indeed, amantadine and rimantadine are no longer recommended, due to the widespread M2-S31N mutant^12,13^. The 2008–2009 seasonal H1N1 strain circulating in the United States and Japan is completely resistant to the only orally bioavailable drug, oseltamivir, owing to H275Y mutation in the neuraminidase^14,15^. The emergence of drug-resistant viruses with acquired fitness of transmission is a timely reminder of the urgent need for new antivirals with a novel mechanism of action and a high genetic barrier to drug resistance.

In the search for novel influenza antivirals, we sought to utilize a fast-track drug discovery program by exploring multi-component reaction (MCR) products for inhibitors that target the influenza polymerase subunit PA_C_-PB1_N_ interactions. As drug discovery involves iterative cycles of design, synthesis, and pharmacological characterization, we envisioned that MCRs would greatly accelerate structure-activity relationship (SAR) studies during the drug discovery process, as final products can be conveniently synthesized in a one-pot one-step reaction, often containing 3 or more point of diversification^16–18^.

**Figure 1:**
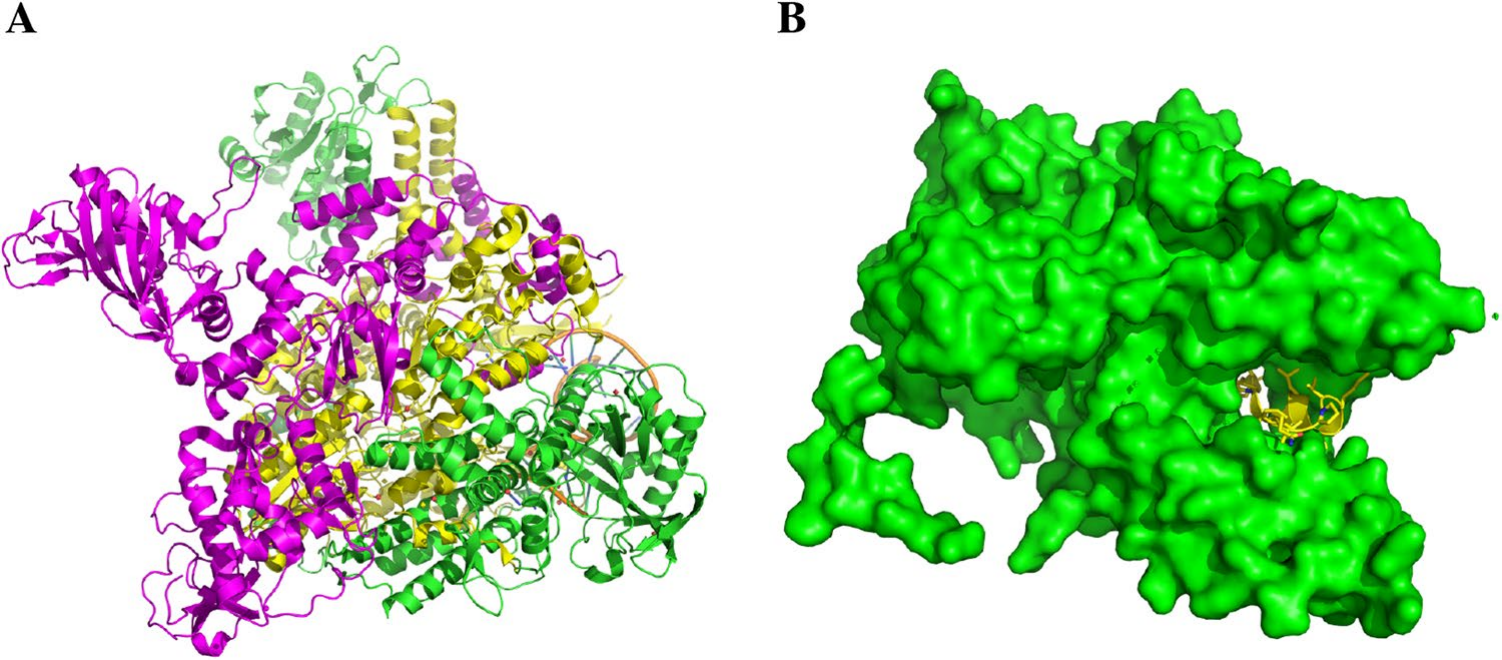
Structures of the influenza polymerase. (**A**) X-ray crystal structures of the viral polymerase complexes from the bat influenza A/H17N10 virus (PDB: 4WSB)^26^. PA: green; PB1: yellow; PB2: magenta. (**B**) X-ray crystal structure of PA_C_-PB1_N_ (PDB: 3CM8)^27^. PA: green; PB1: yellow.

Influenza polymerase consists of three subunits PA, PB1, and PB2 (Fig. 1A)^19,20^. X-ray crystal structures show that the *N*-terminal tail of PB1 (PB1_N_) interacts extensively with the C-terminal domain of PA (PA_C_) (Fig. 1B), and as such the concave shape of the PB1_N_-binding site in PA_C_ presents an attractive target for rational drug design^20,21^. Moreover, the PA_C_ domain is highly conserved among different types and subtypes of influenza viruses, and compounds that inhibit PA_C_-PB1_N_ interactions have shown to have broad-spectrum antiviral activity^22–24^. Using the crystal structure of PA_C_ bound to the PB1_N_ peptide (PDB: 3CM8) as a template, we screened two thousand compounds from an in-house library using the Schrödinger Glide standard precision docking program. The in-house library comprises a diverse set of compounds prepared by multicomponent reaction methodologies^25^. Top hits prioritized by *in silico* docking were tested in a PA_C_-PB1_N_ ELISA assay. Compound **5** was found to inhibit PA_C_-PB1_N_ interaction in a dose-dependent manner with an IC_50_ of 4.3 ± 0.1 µM. The antiviral activity of compound **5** was confirmed by the plaque assay and it had single to submicromolar EC_50_ values against several influenza A and B viruses, including both oseltamivir-sensitive and oseltamivir-resistant strains. Subsequent SAR led to the discovery of **12a** with an improved selectivity index. Similarly, compound **12a** had potent and broad-spectrum antiviral activity against several human clinical isolates of influenza A and B viruses. More importantly, no resistant virus was selected under drug selection pressure of compound **12a**. Overall, we view the discovery of compound **12a** - a broad-spectrum influenza antiviral - as proof-of-concept for the inherent advantage of our in-house fast-track MCR drug discovery platform, which has been recapitulated throughout the drug discovery arena.

## Results and Discussion

### Chemistry

**Figure 2:**
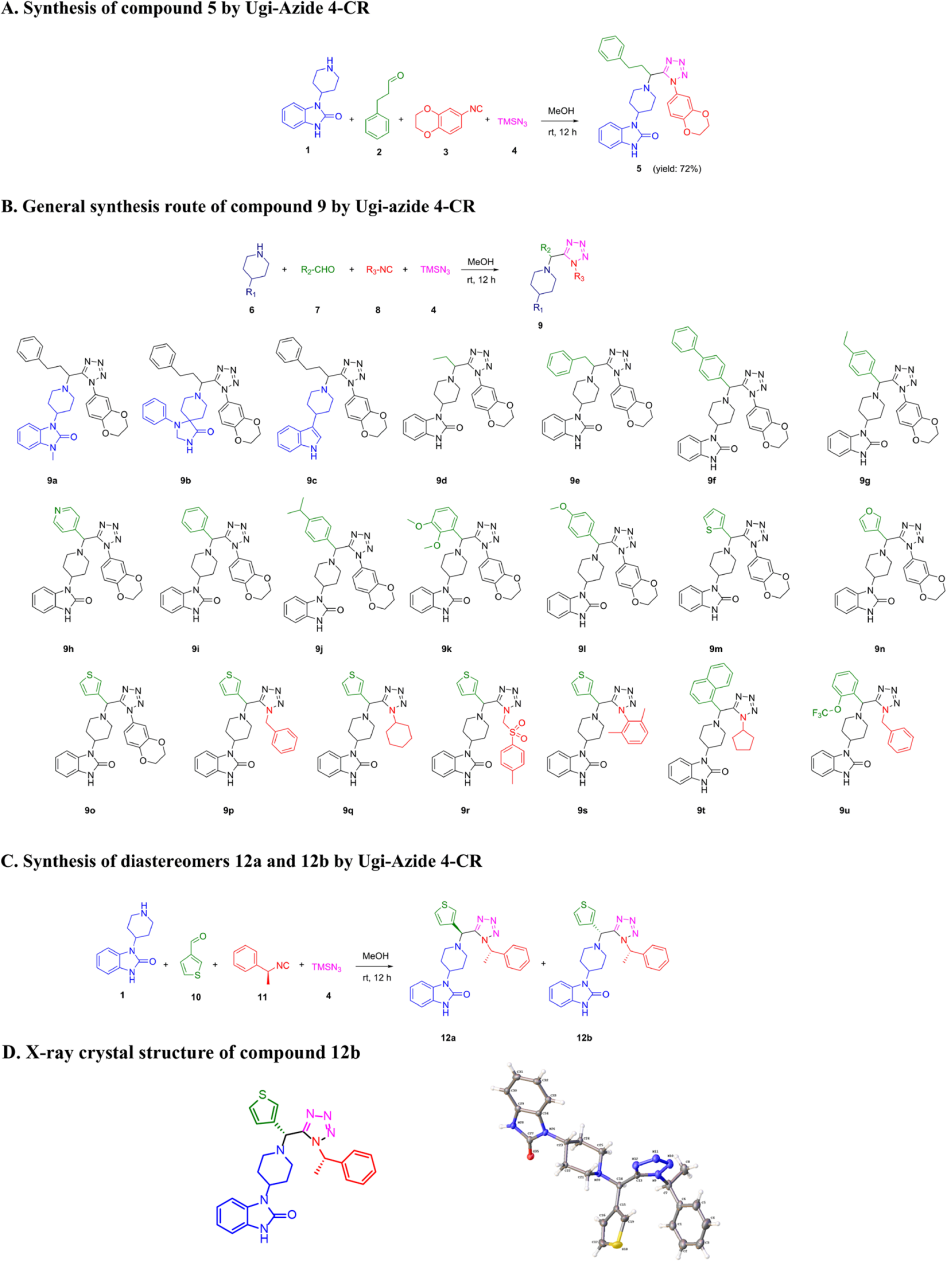
Synthesis routes and structures for compounds 5, 9a-9u and 12a-12b. (**A**) Synthesis of compound 5 by Ugi-azide 4-CR. (**B**) Analogs synthesized for the structure-activity relationship studies. (**C**) Synthesis of diastereomers by the chiral isocyanide strategy. (**D**) X-ray crystal structure of the diastereomer 12b.

Compound **5** was synthesized by the Ugi-azide 4-CR in methanol at room temperature in 72% yield (Fig. 2A). A focused library of compounds **9a**-**9u** with diversity elements amine (R_1_), aldehyde (R_2_), and isocyanide (R_3_) was synthesized according to the general procedure employed to afford compound **5** (Fig. 2B). Compounds **5** and **9a**-**9u** were synthesized and tested as enantiomeric mixtures, whilst compounds **12a** and **12b** were prepared as diastereomeric mixtures (Fig. 2C), and separated by silica gel flash chromatography. The absolute stereochemistry of compound **12b** was determined by X-ray crystallography (Fig. 2D).

### PA_C_-PB1_N_ inhibition and antiviral activity of the initial hit compound 5

**Figure 3:**
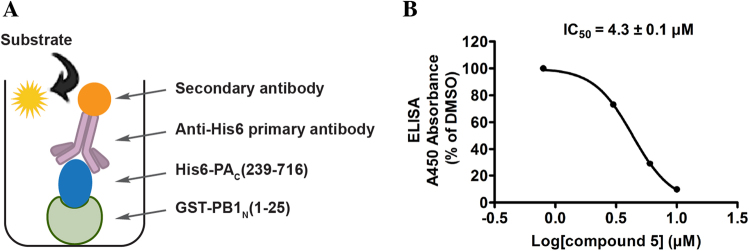
Inhibition of PA_C_-PB1_N_ by compound **5** in ELISA assay. (**A**) Cartoon representation of the PA_C_-PB1_N_ ELISA assay. (**B**) IC_50_ curve of compound **5** in the PA_C_-PB1_N_ ELISA assay.

The initial hit **5** was confirmed as a potent inhibitor of the PA_C_-PB1_N_ polymerase subunit interactions in the ELISA assay with an IC_50_ of 4.3 ± 0.1 µM (Fig. 3) and its antiviral activity was tested in an antiviral plaque assay. Compound **5** was found to inhibit multiple strains of influenza A and B viruses with single to submicromolar EC_50_ values (Table 1). Its cellular cytotoxicity in MDCK cells with a 48 h incubation time was 17.4 ± 1.2 µM; therefore, the antiviral activity was not due to its cellular cytotoxicity.

**Table 1:** Broad-spectrum antiviral activity and cytotoxicity of compound **5**.

Influenza viruses	Drug sensitivity	EC_50_ (µM)	CC_50_ (µM)	SI^a^
A/WSN/33 (H1N1)		1.2 ± 0.6		14.5
A/California/07/2009 (H1N1)	Amantadine resistant Oseltamivir sensitive	4.5 ± 0.7		3.9
A/Denmark/524/2009 (H1N1)		1.8 ± 0.4		9.7
A/Texas/04/2009 (H1N1)	Amantadine resistant	3.5 ± 1.1	17.4 ± 1.2	5.0
A/Washington/29/2009 (H1N1)	Oseltamivir resistant	1.6 ± 0.5		10.9
B/Wisconsin/1/2010 (Yamagata)	Amantadine resistant	0.9 ± 0.2		19.3
B/Brisbane/60/2008 (Victoria)	Oseltamivir sensitive	1.0 ± 0.3		17.4

The values are the mean ± S.D. from two independent experiments. ^a^SI = selectivity index.

### Structure-activity relationship studies of compound 5

**Figure 4:**
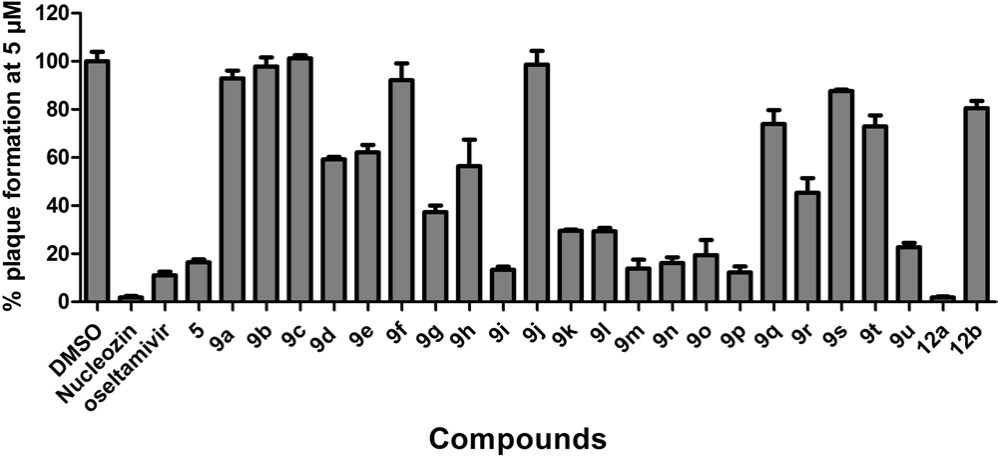
Structure-activity relationship studies of compound **5**. Oseltamivir carboxylate and nucleozin were tested at 200 nM and 1 µM, respectively. All other compounds were tested at 5 µM. The results are the mean ± S.D. from two independent experiments.

Encouraged by the broad-spectrum antiviral activity of **5**, we were thus interested in pursuing SAR studies to further optimize antiviral potency and its selectivity index. To this end, we synthesized a focused library of 21 compounds (**9a**-**9u**) using the expeditious Ugi-azide 4-CR. The library includes compounds that have points of diversity at the amine component R_1_ (**9a**-**9c**), the aldehyde component R_2_ (**9d**-**9o**), and the aldehyde and isocyanide components combined (R_2_ & R_3_) (**9p**-**9u**, and **12a**-**12b**). All compounds were initially tested at 5 µM against the A/WSN/33 (H1N1) virus in the plaque assay to rule out compounds that have no cellular antiviral activity (Fig. 4). Nucleozin and oseltamivir carboxylate, two known influenza antivirals, were included as positive controls. It was found that the amine component, 4-(2-keto-1-benzimidazolinyl)piperidine, is essential for the antiviral activity, as compounds with amine modifications **9a**-**9c** had no antiviral activity (Fig. 4 and Table 2). Amongst compounds with modifications of the aldehyde input R_2_ (**9d**-**9o**), compounds **9i** and **9k**-**9o** had significantly improved antiviral activity compared with compound **5**, and infected cells had less than 30% plaque formation when treated with 5 µM of compound. Compounds **9d**, **9e**, **9 g**, and **9 h** had moderate antiviral activity, while compounds **9 f** and **9j** were not active. These results suggest that hydrophobic aromatic groups such as benzene (**9 g**, **9i**-**9l**), thiophene (**9 m**, **9o**), and furan (**9n**) are preferred at the R_2_ position. When R_2_ substitution was benzene, a methoxyl group was tolerated at the *ortho*-, *meta*-, and *para-*positions (**9k** and **9 l**). A small alkyl group such as ethyl (**9 g**) was also tolerated at the *para*- position; however, branched alkyl group such as isopropyl (**9j**) and a bulky substitution such as benzene (**9 f**) were not tolerated. For compounds with simultaneous modifications at both the aldehyde component R_2_ and the isocyanide component R_3_ (**9p**-**9u**), it was found that benzyl is preferred at R_3_ (**9p**, **9 u** versus **9q**, **9r**, **9 s** and **9t**), and compound **9p** had similar antiviral activity as **9o** (12.3% versus 19.5% plaque formation at 5 µM). Compound **9t** was not active (73.0% plaque formation at 5 µM), possibly due to the presence of the bulky naphthalene substitution at the R_2_ position. Compound **9 u** had antiviral activity similar to compound **5** (22.7% vs 16.5% plaque formation at 5 µM) as it combines favorable substitutions from both the aldehyde component ((*o*-trifluoromethoxy)phenyl) and the isocyanide component (benzyl).

**Table 2:** Antiviral activity and PA-PB1 inhibition of tetrazole analogs.

Compounds	% plaque formation at 5 µM	PA_C_-PB1_N_ ELISA IC_50_ (µM)
**5**	16.5 ± 1.2	4.3 ± 0.1
**9a**	92.9 ± 4.5	N.D.
**9b**	97.9 ± 5.3	N.D.
**9c**	101.3 ± 1.8	26.9 ± 4.1
**9d**	59.3 ± 0.8	N.D.
**9e**	62.2 ± 4.3	N.D.
**9 f**	92.2 ± 9.8	>30
**9 g**	37.4 ± 3.7	N.D.
**9 h**	56.5 ± 12.3	N.D.
**9i**	13.4 ± 0.8	13.6 ± 0.9
**9j**	98.7 ± 8.0	N.D.
**9k**	29.5 ± 1.0	7.0 ± 0.6
**9 l**	29.3 ± 1.3	N.D.
**9 m**	13.8 ± 1.8	15.1 ± 1.2
**9n**	16.1 ± 1.2	N.D.
**9o**	19.5 ± 8.8	9.9 ± 0.7
**9p**	12.3 ± 2.1	N.D.
**9q**	73.9 ± 8.2	N.D.
**9r**	45.4 ± 8.6	N.D.
**9 s**	87.7 ± 0.9	N.D.
**9t**	73.0 ± 6.5	N.D.
**9 u**	22.7 ± 2.2	N.D.
**12a**	1.9 ± 0.3	7.6 ± 1.4
**12b**	80.6 ± 4.5	37.0 ± 7.0
Nucleozin	0	>30
Oseltamivir	11.1 ± 2.1	>30

The results are the mean ± S.D. from two independent experiments. N.D. = not determined.

One feature of Ugi-azide 4-CR is that it produces a new chiral center during the reaction, and hence the product is a mixture of enantiomers. Although the enantiomers could be separated by chiral HPLC or other methods, chiral separation is generally time consuming and expensive, which presents a challenge for future development. Indeed, this somewhat compromises the advantages of exploring these specific chemotypes in the drug discovery arena. With grams of material required for possible downstream pharmacokinetic and *in vivo* animal studies, we therefore sought to develop a convenient synthesis and separation strategy to bypass chiral separation. Hence, we employed a chiral isocyanide, (*S*)-(−)-α-methylbenzyl isocyanide in Ugi-azide 4-CR, which we felt would be well-tolerated based on prior SAR results of **9a**-**9u**. With this chiral isocyanide strategy, a mixture of diastereomers was thus produced (Fig. 2C) that were conveniently separated by silica gel flash column chromatography. The absolute stereochemistry of compound **12b** was determined by X-ray crystallography as (R, S). It was found that the (S, S) diastereomer **12a** had potent antiviral activity (1.9% plaque formation at 5 µM), while the (R, S) diastereomer **12b** was not active (80.6% plaque formation at 5 µM) (Fig. 4).

Selected compounds were also tested for their inhibition of PA_C_-PB1_N_ interactions in the ELISA assay (Table 2). For compounds **9i**, **9k**, **9 m**, **9o**, and **12a**, which had potent antiviral activity (less than 30% plaque formation at 5 µM), the IC_50_ values in the ELISA assay ranged from 7.6 µM to 15.1 µM. For compounds **9c**, **9 f**, and **12b** which had no antiviral activity (greater than 90% plaque formation at 5 µM), the IC_50_ values were above 26.9 µM. Overall, there is in general a positive correlation between the compounds’ PA_C_-PB1_N_ inhibition and their antiviral efficacy. The lack of a straight linear correlation was expected as the antiviral efficacy is a combined effect of PA_C_-PB1_N_ inhibition, cellular permeability, and potential off-target interactions.

Subsequently, the cellular cytotoxicity and broad-spectrum antiviral activity of one of the most potent compounds **12a** was further profiled (Table 3). Compound **12a** was not cytotoxic to either MDCK or A549 cells and the CC_50_ values were greater than 150 µM and 98.1 ± 2.5 µM, respectively. When tested against a panel of human clinical isolates of influenza A and B viruses, compound **12a** showed broad-spectrum antiviral activity, with EC_50_ values ranging from 0.6 µM to 2.7 µM. It is noteworthy that compound **12a** had no cross-resistance with the FDA-approved influenza antivirals amantadine and oseltamivir, as shown by the fact that compound **12a** had potent antiviral activity against viruses that are resistant to amantadine, oseltamivir, or both (Table 3).

**Table 3:** Broad-spectrum antiviral activity and cytotoxicity of compound 12a.

Influenza viruses	Drug sensitivity	EC_50_ (µM)^a^	MDCK CC_50_ (µM)^a^	A549 CC_50_ (µM)^a^	SI^b^
A/WSN/33 (H1N1)		0.7 ± 0.1			>214.3/140.1
A/California/07/2009 (H1N1)	Amantadine resistant	1.3 ± 0.4			>115.4/75.5
A/Denmark/524/2009 (H1N1)	Oseltamivir sensitive	0.8 ± 0.2			>187.5/122.6
A/Switzerland/9715293/2013 (H3N2)		1.4 ± 0.6			>107.1/70.1
A/Texas/04/2009 (H1N1)		1.1 ± 0.4			>136.4/89.2
A/Denmark/528/2009 (H1N1)	Amantadine resistant Oseltamivir resistant	0.9 ± 0.3			>166.7/109.0
A/Washington/29/2009 (H1N1)		0.9 ± 0.3	>150	98.1 ± 2.5	>166.7/109.0
B/Wisconsin/1/2010 (Yamagata)		0.8 ± 0.3			>187.5/122.6
B/Memphis/20/1996 (Yamagata)		2.7 ± 0.2			>55.6/36.3
B/Utah/09/2014 (Yamagata)	Amantadine resistant Oseltamivir sensitive	1.5 ± 0.3			>100/65.4
B/Phuket/3073/2013 (Yamagata)		1.2 ± 0.2			>125/81.8
B/Brisbane/60/2008 (Victoria)		0.6 ± 0.3			>250/163.5

^a^The values are the mean ± S.D. from two independent experiments. ^b^Selectivity index is expressed as MDCK/A549.

### Cellular antiviral mechanism of compound 12a

**Figure 5:**
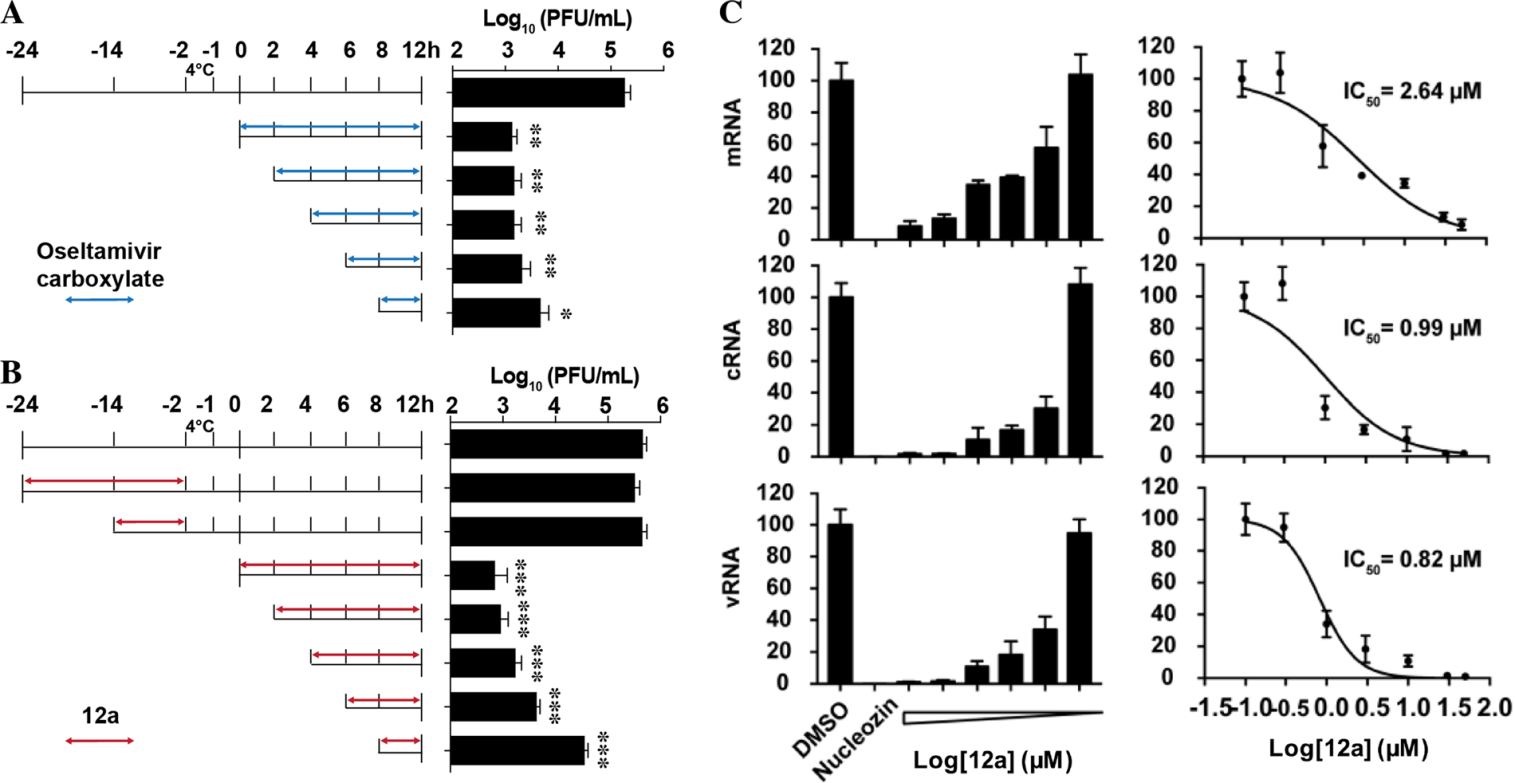
Cellular antiviral mechanism of compound **12a**. (**A**) Time-of-addition experiments with oseltamivir carboxylate. (**B**) Time-of-addition experiments with compound **12a**. For the time-of-addition experiments, MDCK cells were infected with the A/WSN/33 (H1N1) at MOI of 0.01 at −2 h time point; viruses were first incubated at 4 °C for 1 h for attachment followed by 37 °C for 1 h for viral entry. At time point 0 h, cells were washed with PBS buffer and viruses were harvested at 12 h p.i. The titer of harvested virus was determined by plaque assay. Arrows indicate the period in which (**A**) 1 µM oseltamivir carboxylate or (**B**) 10 µM **12a** was present. (**C**) RT-qPCR quantification of the NP mRNA, cRNA and vRNA levels upon compound **12a** treatment. Asterisks indicate statistically significant difference in comparison with the DMSO control (student’s *t*-test, **p < 0.01, ***p < 0.001). The results represent the average of two repeats ± standard deviation.

To gain insights into the cellular antiviral mechanism of **12a**, we performed time-of-addition and RT-qPCR. As **12a** was confirmed to inhibit influenza polymerase PA-PB1 interactions, it is expected to inhibit the intermediate stage of viral replication post viral fusion in the time-of-addition experiment. Furthermore, when the viral polymerase is inhibited, viral RNA transcription and replication should also be reduced. To confirm which stage of viral replication was affected by **12a**, we performed a time-of-addition experiment by adding **12a** at different time points during viral replication (Fig. 5A and B). Thus, it was found that the efficacy of **12a** gradually decreased when it was added at later stages of viral replication (Fig. 5B). Pre-treatment of cells with **12a** has little to no effect on viral replication, indicating that **12a** might not target the host factors (Fig. 5B). As a control, oseltamivir carboxylate retained potent antiviral activity even when it was added 8 h post viral infection (Fig. 5A), which is consistent with its known mechanism of inhibiting viral egress. Next, to test whether **12a** inhibits viral RNA transcription and replication, we performed RT-qPCR assay. Quantification of the viral nucleoprotein (NP) RNA expression levels by RT-qPCR revealed that the levels of vRNA, cRNA, and mRNA were all inhibited in a dose-dependent manner by **12a** (Fig. 5C). The IC_50_ values are consistent with the antiviral efficacy EC_50_ values of compound **12a**. Collectively, the results from the time-of-addition experiment and RT-qPCR are consistent with the antiviral mechanism of **12a** by inhibiting the viral polymerase PA-PB1 interactions.

### Compound 12a has a higher in vitro genetic barrier to drug resistance than oseltamivir

**Figure 6:**
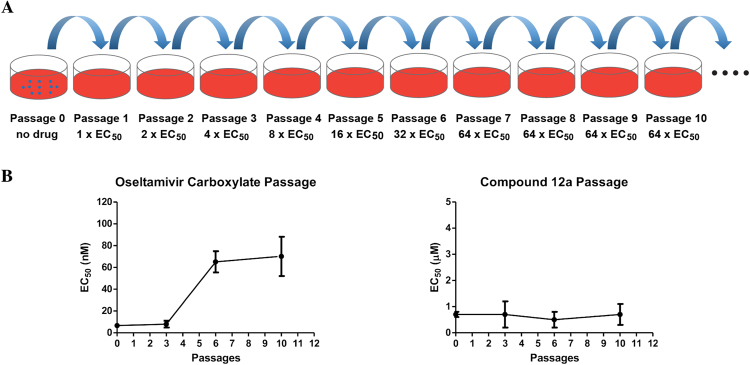
Compound **12a** has a high *in vitro* genetic barrier to drug resistance as shown by the serial viral pasage experiment. (**A**) Cartoon representation of the serial viral passage experiment. (**B**) Comparison of the *in vitro* genetic barrier of drug resistance between oseltamivir carboxylate and compound **12a**.

Drug-induced resistance is one of the major obstacles facing antiviral drugs^10^. We therefore designed serial viral passage experiments to characterize the genetic barrier to drug resistance of compound **12a** (Fig. 6)^28–31^. In this experiment, the A/WSN/33 (H1N1) virus was amplified in the presence of increasing concentrations of compound **12a** and the drug sensitivity of the resulting viruses at different passages was assayed against compound **12a** using a plaque assay. Oseltamivir carboxylate was included as a control. Gratifyingly, viruses at passage 10 remained sensitive to compound **12a**, and no increase in EC_50_ value was observed (Fig. 6B and Table 4). In contrast, the EC_50_ for oseltamivir carboxylate increased 10-fold at passage six and onwards, which is consistent with previous reports^32,33^. These results suggest that compound **12a** targets a vital viral replication component such as the viral polymerase that is less prone to mutate. Overall, compound **12a** demonstrated a high *in vitro* genetic barrier to drug resistance, rendering it a desirable drug candidate for further development.

**Table 4:** *In vitro* serial drug passage experiment with compound 12a.

Passage #	Oseltamivir carboxyalte applied (nM)	EC_50_ (nM)	Compound **12a** applied (µM)	EC_50_ (µM)
0		6.7 ± 1.8		0.7 ± 0.1
1	10	N.D.	1	N.D.
2	20	N.D.	2	N.D.
3	40	7.9 ± 3.1	4	0.7 ± 0.5
4	80	N.D.	8	N.D.
5	160	N.D.	16	N.D.
6	320	60.1 ± 14.7	32	0.5 ± 0.3
7	640	N.D.	64	N.D.
8	640	N.D.	64	N.D.
9	640	N.D.	64	N.D.
10	640	70.2 ± 18.0	64	0.7 ± 0.4

Passage was performed using the A/WSN/33 (H1N1) virus by following our reported procedure^29–31^. Oseltamivir carboxylate was included as a control. The EC_50_ values at selected passages were determined by plaque assay. The values are the mean ± S.D. from two independent experiments. N.D. = not determined.

### Docking model of compound 12a in PA_c_

**Figure 7:**
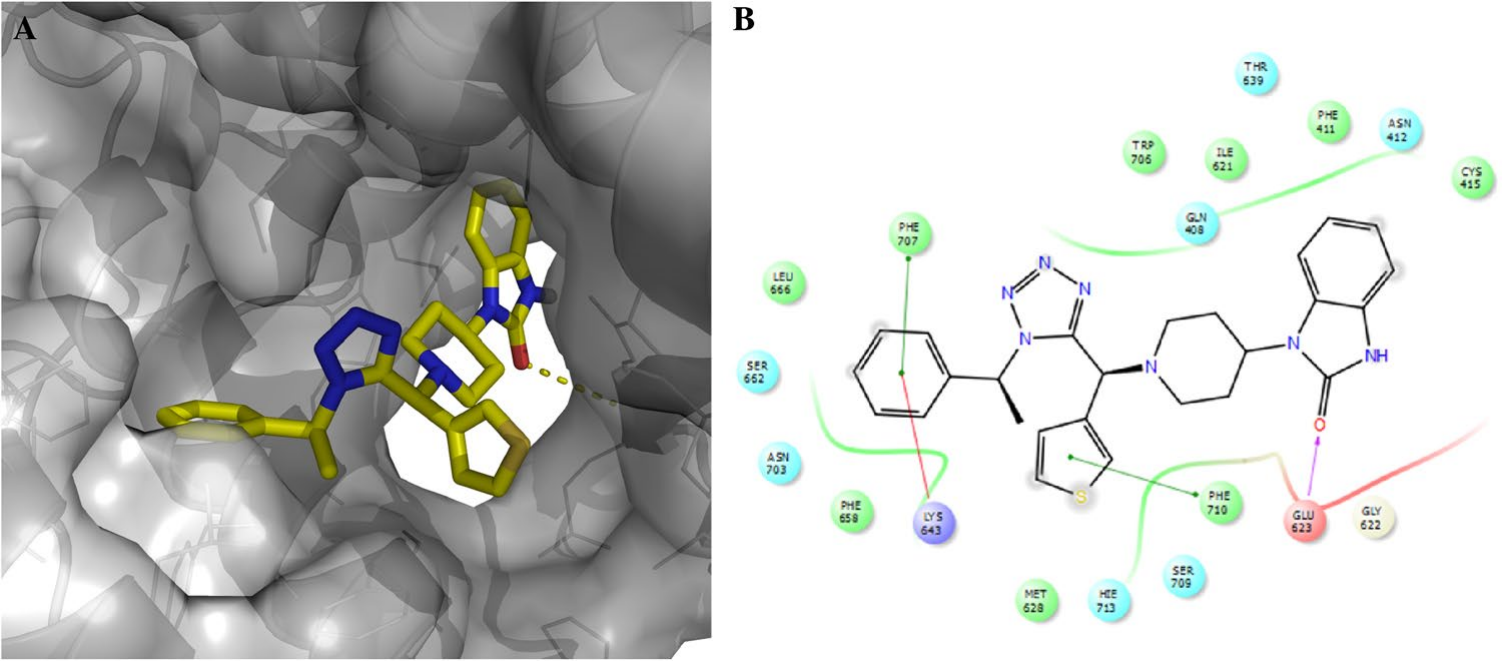
Docking model of compound **12a** in the PB1_N_-binding pocket in PA_C_. (**A**) Surface view of the docking model of compound **12a** in PA_C_. (**B**) Ligand interaction diagram of compound **12a** with residues in the binding site.

To gain insights on how compound **12a** binds to the PB1_N_-binding pocket in PA_C_, we performed molecular docking using Schrödinger Glide software. In the docking model of **12a** in PA, **12a** snuggly fits in the PB1-binding pocket in PA (Fig. 7A), forming extensive hydrophobic interactions and multiple π–π interactions (Fig. 7B). For example, the phenyl ring from the isocyanide input (R_3_) in **12a** interacts with F707 and K643 through π–π and cation–π interactions, respectively. The thiophene ring from **12a** forms π–π interactions with F710. In addition, the carbonyl from the benzimidazol-2-one in **12a** forms a hydrogen bond with the E623 backbone amide NH, while the benzene ring of the benzimidazol-2-one fits in the hydrophobic pocket formed by F411 and I621.

## Conclusions

Drug discovery is an expensive and time-consuming endeavor. On average, it costs billions of dollars and 10–15 years to advance a drug to the market^34^. Development of antiviral drugs comes with an even greater challenge because of the intrinsic issue of drug resistance, which could render years of research efforts futile^14^. To shorten the length of antiviral drug development, we are interested in further building our fast-track drug discovery platform by exploiting MCR products to identify hits with broad-spectrum antiviral activity and a high genetic barrier to drug resistance. Exploring MCRs for antiviral drug discovery offers the key advantage of expeditious synthesis that could feasibly enable timely development of newer generations of antivirals if resistance to earlier generations were to emerge. Toward this goal, we launched an *in silico* virtual screening campaign of an in-house, MCR-derived small-molecule library^25,35,36^ against the highly conserved PA_C_ subunit of the influenza polymerase. The rationale for choosing this library were: (1) compounds in this library were synthesized by one-pot MCR reactions, which will ensure expeditious turnover of subsequent structure–activity relationship (SAR) studies; (2) the synthesis methodologies for compounds in this library are established and optimized, which ensures expeditious re-synthesis of interesting hits in gram quantities for subsequent mechanistic studies and *in vivo* animal studies and (3) the tetrazole ring embedded in all the molecules are *cis*-amide bond isosteres and as such are attractive constrained peptidomimetics^37^. As a proof-of-concept, our efforts have already yielded several hits. For example, compound **5** (Table 1) is active against multiple influenza A and B strains. Subsequent SAR by the expeditious Ugi-azide 4-CR led to the discovery of compound **12a** that: (1) inhibits PA_C_-PB1_N_ interactions in the ELISA assay, (2) has broad-spectrum antiviral activity against a panel of human clinical isolates of influenza A and B viruses, (3) has high selectivity index, and (4) has a high *in vitro* genetic barrier to drug resistance. The cellular antiviral mechanism of **12a** was further confirmed by time-of-addition and RT-qPCR experiments. Importantly, by introducing a chiral isocyanide as the building block in the Ugi-azide 4-CR, we circumvent the problem of chiral separation and the diastereomeric product mixture was conveniently separated by silica gel flash chromatography. In summary, the promising *in vitro* antiviral efficacy of compound **12a** warrants its further development as a next-generation influenza antiviral.

### Experimental Section

#### Molecular Docking

The X-ray crystal structure of PAc-PB1_N_ (PDB: 3CM8) was used as the protein template for the molecular docking. Docking was performed using Schrödinger Glide. In house small molecule library was processed in Ligprep and all possible enantiomers and diasteromers were generated and docked individually. The grid box center was set as the centroid of workspace ligand (PB1_N_). The grid box size was set to dock ligands similar in size to the workspace ligand (PB1_N_). All compounds were initially docked using standard precision algorithm, and top 10% of the hits were further docking using extra precision algorithm. Hits were ranked by Glide score.

#### Chemistry

All commercially available chemicals were used without further purification. All final compounds were purified by flash column chromatography. Compound **5** and **9a**-**9u** were synthesized and tested as a mixture of enantiomers. Compounds **12a** and **12b** were separated as pure diastereomers and tested individually. ^1^H and ^13^C NMR spectra were recorded on a Bruker-400 NMR spectrometer. Chemical shifts are reported in parts per million referenced with respect to residual solvent (DMSO-d6) 2.50 ppm and (Chloroform-d) 7.26 ppm or from internal standard tetramethylsilane (TMS) 0.00 ppm. The following abbreviations were used in reporting spectra: s, singlet; d, doublet; t, triplet; q, quartet; m, multiplet; dd, doublet of doublets; ddd, doublet of doublet of doublets. HPLC-grade solvents were used for all reactions. Flash column chromatography was performed using silica gel (230–400 mesh, Merck). Low-resolution mass spectra were obtained using an ESI technique on a 3200 Q Trap LC/MS/MS system (Applied Biosystems). High-resolution mass spectra were obtained using the positive ESI method for all the compounds, obtained in an Ion Cyclotron Resonance (ICR) spectrometer. The purity was assessed by using a Shimadzu LC-MS with a Waters XTerra MS C-18 column (part #186000538), 50 × 2.1 mm, at a flow rate of 0.3 mL/min; λ = 250 and 220 nm; mobile phase A, 0.1% formic acid in H_2_O, and mobile phase B’, 0.1% formic in 60% isopropanol, 30% CH_3_CN and 9.9% H_2_O. All compounds submitted for testing in the ELISA and plaque assays were confirmed to be >95.0% purity by LC-MS traces. All compounds were characterized by proton and carbon NMR and MS.

#### Synthesis Procedures

Amine (1.0 equiv) and aldehyde (1.0 equiv) were mixed in methanol (5 ml) and stirred at room temperature for 15 minutes. Then TMS-azide (1.0 equiv) and isocyanide (1.0 equiv) were added sequentially and the resulting mixture was stirred at room temperature overnight. After that, the solvent was removed under reduced pressure and the crude product was purified with flash silica gel chromatography (Ethyl acetate in hexane 20–70%).

*1‐(1‐{1‐[1‐(2,3‐dihydro‐1,4‐benzodioxin‐6‐yl)‐1,2,3,4‐tetrazol‐5‐yl]‐3‐phenylpropyl}piperidin‐4*‐ *yl)‐3* *H‐1*,*3‐benzodiazol‐2‐one (****5****)*. White solid. Yield: 65%. ^1^H NMR (400 MHz, CDCl_3_) *δ* 10.31 (s, 1 H), 7.27–6.89 (m, 12 H), 4.24–4.22 (m, 5 H), 3.95–3.85 (m, 1 H), 2.97–2.95 (m, 1 H), 2.86–2.19 (m, 9 H), 1.84–1.70 (m, 2 H); ^13^C NMR (101 MHz, CDCl_3_) *δ* 144.8, 143.5, 134.8, 133.6, 130.2, 118.6, 118.1, 117.9, 117.7, 116.7, 115.8, 110.9, 110.6, 107.8, 107.6, 104.3, 99.5, 99.0, 54.0, 53.9, 46.7, 40.3, 38.6, 37.5, 22.1, 19.2, 19.1, 18.6; C_30_H_31_N_7_O_3_ HRMS (ESI) m/z calculated for [M + H]^+^  = 538.25611, found 538.25612.

*1‐(1‐{1‐[1‐(2*,*3‐dihydro‐1*,*4‐benzodioxin‐6‐yl)‐1* *H‐1*,*2*,*3*,*4‐tetrazol‐5‐yl]‐3‐phenylpropyl}piperidin‐4‐yl)‐3‐methyl‐2*,*3‐dihydro‐1* *H‐1*,*3‐benzodiazol‐2‐one* (***9a***). Beige oil. Yield: 89%. ^1^H NMR (400 MHz, DMSO-d_6_) *δ* 7.28–7.24 (m, 3 H), 7.18–7.10 (m, 5 H), 7.07–7.06 (m, 2 H), 7.04–7.02 (m, 2 H), 4.33 (2, 4 H), 4.14–3.96 (m, 2 H), 3.28 (s, 3 H), 2.87 (d, *J* = 12.1 Hz, 1 H), 2.66–2.59 (m, 2 H), 2.46 (d, *J* = 9.4 Hz, 1 H), 2.31–2.05 (m, 5 H), 1.63 (d, *J* = 13.8 Hz, 1 H), 1.52 (d, *J* = 11.5 Hz, 1 H), 1.33–1.17 (m, 1 H); ^13^C NMR (101 MHz, DMSO-d_6_) *δ* 154.1, 152.9, 144.9, 143.6, 141.1, 129.7, 128.3, 128.2, 127.8, 126.7, 125.9, 120.7, 120.6, 118.5, 117.6, 114.7, 108.6, 107.8, 64.22, 64.15, 56.3, 50.5, 47.9, 47.7, 31.8, 29.03, 28.97, 26.7; C_31_H_33_N_7_O_3_ HRMS (ESI) m/z calculated for [M + H]^+^  = 552.27245, found 552.27176.

*8‐{1‐[1‐(2*,*3‐dihydro‐1*,*4‐benzodioxin‐6‐yl)‐1* *H‐1*,*2*,*3*,*4‐tetrazol‐5‐yl]‐3‐phenylpropyl}‐1‐phenyl‐ 1*,*3*,*8‐triazaspiro[4*.*5]decan‐4‐one* (***9b***). White solid. Yield: 66%. ^1^H NMR (400 MHz, DMSO-d_6_) *δ* 8.62 (s, 1 H), 7.31 (d, *J* = 2.3 Hz, 1 H), 7.28–7.13 (m, 7 H), 7.12–7.01 (m, 2 H), 6.81–6.68 (m, 3 H), 4.53 (s, 2 H), 4.33–4.30(m, 4 H), 3.95–3.92 (m, 1 H), 3.08–3.02 (m, 1 H), 2.89–2.78 (m, 2 H), 2.62–2.57 (m, 3 H), 2.49–2.40 (m, 1 H), 2.38–2.22 (m, 3 H), 1.56 (d, *J* = 13.2 Hz, 1 H), 1.50–1.41 (m, 1 H); ^13^C NMR (101 MHz, DMSO-d_6_) *δ* 176.0, 154.1, 144.9, 143.7, 143.2, 141.1, 128.9, 128.3, 128.2, 126.7, 125.9, 118.3, 117.6, 117.5, 114.4, 113.9, 64.2, 64.1, 58.6, 58.1, 56.4, 45.4, 44.2, 31.8, 28.7, 28.6; C_31_H_33_N_7_O_3_ HRMS (ESI) m/z calculated for [M + H]^+^  = 552.27176, found 552.27210.

*3‐(1‐{1‐[1‐(2*,*3‐dihydro‐1*,*4‐benzodioxin‐6‐yl)‐1 H‐1*,*2*,*3*,*4‐tetrazol‐5‐yl]‐3‐phenylpropyl}piperidin‐4‐yl)‐1 H‐indole (****9c****)*. White solid. Yield: 75%. ^1^H NMR (400 MHz, CDCl_3_) δ 8.02 (s, 1 H), 7.66–7.57 (m, 1 H), 7.41–7.34 (m, 1 H), 7.30–7.23 (m, 3 H), 7.23–7.08 (m, 6 H), 7.00–6.92 (m, 3 H), 4.46–4.21 (m, 4 H), 3.92 (dd, *J* = 8.6, 5.4, 1 H), 2.97 (d, *J* = 11.4, 1 H), 2.84–2.72 (m, 2 H), 2.74–2.62 (m, 2 H), 2.60–2.46 (m, 2 H), 2.45–2.26 (m, 2 H), 2.11 (d, *J* = 13.1, 1 H), 1.97 (d, *J* = 12.8, 1 H), 1.83–1.56 (m, 2 H). ^13^C NMR (101 MHz, CDCl_3_) δ 154.10, 145.11, 143.93, 140.88, 136.41, 128.47, 128.37, 127.18, 126.55, 126.09, 121.94, 121.23, 119.64, 119.14, 119.11, 118.35, 117.87, 114.81, 111.21, 64.40, 64.29, 57.41, 49.81, 49.09, 33.58, 33.42, 33.06, 32.61, 29.12; C_31_H_32_N_6_O_2_ HRMS (ESI) m/z calculated for [M + H]^+^  = 521.25867, found 521.26690.

*1‐(1‐{1‐[1‐(2*,*3‐dihydro‐1*,*4‐benzodioxin‐6‐yl)‐1 H‐1*,*2*,*3*,*4‐tetrazol‐5‐yl]propyl}piperidin‐4‐yl)‐2*,*3‐dihydro‐1* *H‐1*,*3‐benzodiazol‐2‐one (****9d****)*. Beige solid. Yield: 34%. ^1^H NMR (400 MHz, DMSO-d_6_) *δ* 10.79 (s, 1 H), 7.35–7.34 (m, 1 H), 7.17–7.11 (m, 2 H), 7.08–7.05 (m, 1 H), 6.99–6.94 (m, 3 H), 4.34 (s, 4 H), 4.13–3.93 (m, 2 H), 2.90 (d, *J* = 10.4 Hz, 1 H), 2.51–2.46 (m, 3 H), 2.23–1.99 (m, 4 H), 1.62 (d, *J* = 13.7 Hz, 1 H), 1.51 (d, *J* = 11.8 Hz, 1 H), 0.89 (t, *J* = 7.3 Hz, 3 H); ^13^C NMR (100 MHz, DMSO-d_6_) *δ* 154.3, 153.6, 144.9, 143.7, 129.1, 128.3, 127.0, 120.5, 120.3, 118.6, 117.7, 114.7, 108.8, 108.6, 64.2, 58.9, 49.9, 48.4, 47.5, 29.0, 28.9, 20.7, 11.2; C_24_H_27_N_7_O_3_ HRMS (ESI) m/z calculated for [M + H]^+^  = 462.22481, found 462.22522.

*1‐(1‐{1‐[1‐(2*,*3‐dihydro‐1*,*4‐benzodioxin‐6‐yl)‐1 H‐1*,*2*,*3*,*4‐tetrazol‐5‐yl]‐2‐phenylethyl}piperidin‐4‐yl)‐2*,*3‐dihydro‐1 H‐1*,*3‐benzodiazol‐2‐one (****9e****)*. White solid. Yield: 75%. ^1^H NMR (400 MHz, DMSO-*d*_6_) δ 10.81 (s, 1 H), 7.30–7.13 (m, 5 H), 7.08–6.92 (m, 5 H), 6.83 (d, *J* = 2.5 Hz, 1 H), 6.71 (dd, *J* = 8.6, 2.5 Hz, 1 H), 4.35–4.23 (m, 5 H), 4.07–3.96 (m, 1 H), 3.44–3.27 (m, 2 H), 3.07 (d, *J* = 11.4 Hz, 1 H), 2.65 (d, *J* = 11.3 Hz, 1 H), 2.50–2.42 (m, 1 H), 2.30 (dd, *J* = 12.2, 9.8 Hz, 1 H), 2.22–2.01 (m, 2 H), 1.64 (d, *J* = 11.9 Hz, 1 H), 1.54 (d, *J* = 11.7 Hz, 1 H). ^13^C NMR (101 MHz, DMSO-*d*_6_) δ 154.36, 154.05, 145.46, 144.08, 138.50, 129.77, 129.53, 128.80, 128.76, 126.96, 126.88, 120.97, 120.76, 118.85, 118.10, 114.93, 114.26, 109.28, 109.07, 64.67, 64.61, 60.14, 50.27, 49.07, 48.45, 35.35, 29.42, 29.30; C_29_H_29_N_7_O_3_ HRMS (ESI) m/z calculated for [M + H]^+^  = 524.23319, found 524.24136.

*1‐[1‐({[1*,*1′‐biphenyl]‐4‐yl}[1‐(2*,*3‐dihydro‐1*,*4‐benzodioxin‐6‐yl)‐1 H‐1*,*2*,*3*,*4‐tetrazol‐5‐yl]methyl)piperidin‐4‐yl]‐2*,*3‐dihydro‐1 H‐1*,*3‐benzodiazol‐2‐one (****9 f****)*. White solid. Yield: 62%. ^1^H NMR (400 MHz, DMSO-*d*_6_) δ 10.81 (s, 1 H), 7.74–7.65 (m, 4 H), 7.56–7.43 (m, 4 H), 7.42–7.33 (m, 1 H), 7.24 (d, *J* = 2.5 Hz, 1 H), 7.17–6.92 (m, 6 H), 5.27 (s, 1 H), 4.41–4.22 (m, 4 H), 4.06–3.84 (m, 1 H), 2.89 (dd, *J* = 30.1, 10.5 Hz, 2 H), 2.35–2.03 (m, 4 H), 1.66–1.53 (m, 2 H). ^13^C NMR (101 MHz, DMSO-*d*_6_) δ 154.93, 154.07, 145.64, 144.15, 140.44, 140.06, 134.07, 130.30, 129.62, 129.43, 128.74, 128.07, 127.17, 126.98, 120.98, 120.77, 119.35, 118.15, 115.46, 109.25, 109.01, 64.74, 64.63, 62.55, 50.28, 50.16, 49.28, 29.21, 29.11; C_34_H_31_N_7_O_3_ HRMS (ESI) m/z calculated for [M + H]^+^  = 586.24884, found 586.25730.

*1‐(1‐{[1‐(2*,*3‐dihydro‐1*,*4‐benzodioxin‐6‐yl)‐1* *H‐1*,*2*,*3*,*4‐tetrazol‐5‐yl](4‐ethylphenyl)methyl}piperidin‐4‐yl)‐2*,*3‐dihydro‐1* *H‐1*,*3‐benzodiazol‐2‐one* (***9 g***). White solid. Yield: 69%. ^1^H NMR (400 MHz, DMSO-*d*_6_) δ 10.80 (s, 1 H), 7.32 (d, *J* = 8.2 Hz, 2 H), 7.29–7.15 (m, 3 H), 7.15–7.06 (m, 2 H), 7.04–6.92 (m, 4 H), 5.14 (s, 1 H), 4.44–3.78 (m, 5 H), 2.84 (dd, *J* = 26.0, 10.1 Hz, 2 H), 2.62 (q, *J* = 7.6 Hz, 2 H), 2.31–2.13 (m, 3 H), 2.02 (t, *J* = 11.4 Hz, 1 H), 1.57 (t, *J* = 14.4 Hz, 2 H), 1.20 (t, *J* = 7.6 Hz, 3 H). ^13^C NMR (101 MHz, DMSO-*d*_6_) δ 155.13, 154.07, 145.62, 144.19, 144.14, 132.29, 129.60, 128.73, 128.10, 127.12, 120.97, 120.77, 119.32, 118.14, 115.43, 114.26, 109.24, 109.00, 64.73, 64.62, 62.69, 50.31, 50.13, 49.34, 29.16, 29.07, 28.28, 15.88; C_30_H_31_N_7_O_3_ HRMS (ESI) m/z calculated for [M + H]^+^  = 538.24884, found 538.25708.

*1‐(1‐{[1‐(2,3‐dihydro-1,4-benzodioxin-6-yl)-1 H-1,2,3,4-tetrazol-5-yl](pyridin-4-yl)methyl}piperidin-4-yl)-2,3-dihydro-1 H-1,3-benzodiazol-2-one (****9 h****)*. Whites solid. Yield: 43%. ^1^H NMR (400 MHz, DMSO-d_6_) *δ* 10.08 (s, 1 H), 8.60 (d, *J* = 6.0 Hz, 2 H), 7.61–7.37 (d, *J* = 6.0 Hz, 2 H), 7.30 (d, *J* = 2.1 Hz, 1 H), 7.19–7.03 (m, 3 H), 6.97 (m, 3 H), 5.39 (s, 1 H), 4.34 (t, *J* = 3.3 Hz, 4 H), 4.18–3.81 (m, 1 H), 2.88 (d, *J* = 9.3 Hz, 1 H), 2.74 (d, *J* = 10.0 Hz, 1 H), 2.35–1.91 (m, 4 H), 1.72–1.40 (m, 2 H). ^13^C NMR (100 MHz, DMSO-d_6_) δ 153.58, 153.23, 149.56, 145.17, 143.66, 142.55, 129.09, 128.27, 126.75, 124.25, 120.52, 120.28, 118.83, 117.65, 114.93, 108.80, 108.55, 79.16, 64.28, 64.16, 61.14, 49.60, 49.32, 48.45, 28.73, 28.66; C_27_H_26_N_8_O_3_ HRMS (ESI) m/z calculated for [M + H]^+^  = 511.21279, found 511.22126.

*1*-*(1*-*{[1*-*(2*,*3*-*dihydro‐1*,*4‐benzodioxin*-*6‐yl)*‐*1* *H‐1*,*2*,*3*,*4‐tetrazol‐5‐yl](phenyl)methyl}piperidin‐4‐yl)‐2*,*3‐dihydro‐1 H‐1*,*3‐benzodiazol‐2‐one (****9i****)*. White solid. Yield: 88%. ^1^H NMR (400 MHz, DMSO-d_6_) *δ* 10.79 (s, 1 H), 7.41–7.33 (m, 5 H), 7.19–7.18 (m, 1 H), 7.11–7.07 (m, 2 H), 7.01–6.94 (m, 4 H), 5.19 (s, 1 H), 4.36–4.33 (m, 4 H), 4.02–3.94 (m, 1 H), 2.87 (d, *J* = 9.7 Hz, 1 H), 2.80 (d, *J* = 11.9 Hz, 1 H), 2.51–2.50 (m, 1 H), 2.28–2.12 (m, 2 H), 1.61–1.52 (m, 1 H); ^13^C NMR (101 MHz, DMSO-d_6_) *δ* 154.5, 153.6, 145.1, 143.7, 134.5, 129.2, 129.1, 128.3, 128.2, 126.7, 120.5, 120.3, 118.8, 117.7, 115.0, 108.8, 108.5, 64.3, 64.1, 62.4, 59.7, 49.8, 49.6, 48.8, 28.6, 20.7, 14.1, 10.8; C_28_H_27_N_7_O_3_ HRMS (ESI) m/z calculated for [M + H]^+^  = 510.22481, found 510.22513.

*1‐(1‐{[1‐(2*,*3‐dihydro‐1*,*4‐benzodioxin‐6‐yl)‐1* *H‐1*,*2*,*3*,*4‐tetrazol‐5‐yl][4‐(propan‐2‐yl)phenyl]methyl}piperidin‐4‐yl)‐2*,*3‐dihydro‐1 H‐1*,*3‐benzodiazol‐2‐one (****9j****)*. White solid. Yield: 65%. ^1^H NMR (400 MHz, DMSO-*d*_6_) δ 10.80 (s, 1 H), 7.33 (d, *J* = 8.3 Hz, 2 H), 7.26 (d, *J* = 8.3 Hz, 2 H), 7.21–7.07 (m, 3 H), 7.05–6.92 (m, 4 H), 5.14 (s, 1 H), 4.48–4.24 (m, 4 H), 4.07–3.89 (m, 1 H), 2.94–2.77 (m, 3 H), 2.30–2.10 (m, 3 H), 2.03 (t, *J* = 11.5 Hz, 1 H), 1.64–1.52 (m, 2 H), 1.22 (d, *J* = 6.9 Hz, 6 H). ^13^C NMR (101 MHz, DMSO-*d*_6_) δ 155.15, 154.07, 148.77, 145.62, 144.13, 132.43, 129.60, 128.73, 127.13, 126.63, 120.97, 120.77, 119.35, 118.13, 115.46, 109.24, 109.01, 64.73, 64.62, 62.67, 50.30, 50.16, 49.35, 33.58, 29.16, 29.06, 24.29, 24.24; C_31_H_33_N_7_O_3_ HRMS (ESI) m/z calculated for [M + H]^+^  = 552.26449, found 552.27210.

*1‐(1‐{[1‐(2*,*3‐dihydro‐1*,*4‐benzodioxin‐6‐yl)‐1 H‐1*,*2*,*3*,*4‐tetrazol‐5‐yl](2*,*3‐dimethoxyphenyl)methyl}piperidin‐4‐yl)‐2*,*3‐dihydro‐1 H‐1*,*3‐benzodiazol‐2‐one (****9k****)*. White solid. Yield: 56%. ^1^H NMR (400 MHz, CDCl_3_) *δ* 10.00 (s, 1 H), 7.32–7.29 (m, 1 H), 7.18–7.16 (m, 1 H), 7.10–7.06 (m, 2 H), 7.04–6.98 (m, 4 H), 6.92–6.85 (m, 2 H), 5.56 (s, 1 H), 4.35–4.30 (m, 4 H), 4.26–4.21 (m, 1 H), 3.87 (s, 3 H), 3.64 (s, 3 H), 3.10–2.98 (m, 2 H), 2.54–2.34 (m, 3 H), 2.32–2.16 (m, 1 H), 1.84–1.71 (m, 2 H); ^13^C NMR (101 MHz, CDCl_3_) *δ* 155.2, 154.8, 152.7, 147.3, 145.5, 144.1, 129.2, 129.0, 128.2, 127.0, 123.9, 122.1, 121.3, 121.1, 119.1, 118.1, 115.4, 112.7, 109.8, 109.7, 64.5, 64.4, 60.7, 56.0, 55.9, 50.8, 50.5, 50.1, 29.6; C_30_H_31_N_7_O_5_ HRMS (ESI) m/z calculated for [M + H]^+^  = 570.24594, found 570.24716.

*1‐(1‐{[1‐(2*,*3‐dihydro‐1*,*4‐benzodioxin‐6‐yl)‐1 H‐1*,*2*,*3*,*4‐tetrazol‐5‐yl](4‐methoxyphenyl)methyl}piperidin‐4‐yl)‐2*,*3‐dihydro‐1 H‐1*,*3‐benzodiazol‐2‐one* (***9l***). White solid. Yield: 76%. ^1^H NMR (400 MHz, CDCl_3_) δ 10.16 (s, 1 H), 7.35 (d, J = 8.3 Hz, 2 H), 7.26–7.15 (m, 1 H), 7.15–6.96 (m, 4 H), 6.96–6.84 (m, 3 H), 6.77 (dd, J = 8.6, 2.5 Hz, 1 H), 4.89 (s, 1 H), 4.34 (t, J = 3.8 Hz, 4 H), 4.31 (s, 1 H), 3.81 (s, 3 H), 3.07 (d, J = 11.1 Hz, 1 H), 2.96 (d, J = 7.0 Hz, 1 H), 2.58–2.30 (m, 3 H), 2.11 (m, 1 H), 1.75 (m, 2 H). ^13^C NMR (101 MHz, CDCl_3_) δ 159.85, 155.25, 154.95, 145.63, 144.16, 130.59, 129.15, 128.19, 126.89, 126.79, 121.30, 121.11, 118.95, 118.15, 115.27, 114.10, 109.88, 109.73, 64.53, 64.43, 63.19, 55.44, 50.74, 50.50, 49.99, 29.47, 29.35; C_29_H_29_N_7_O_4_ HRMS (ESI) m/z calculated for [M + H]^+^  = 540.22810, found 540.23656.

*1‐(1‐{[1‐(2*,*3‐dihydro‐1*,*4‐benzodioxin‐6‐yl)‐1* *H‐1*,*2*,*3*,*4‐tetrazol‐5‐yl](thiophen‐2‐yl)methyl}piperidin‐4‐yl)‐2*,*3‐dihydro‐1* *H‐1*,*3‐benzodiazol‐2‐one* (***9 m****)*. White solid. Yield: 61%. ^1^H NMR (400 MHz, CDCl_3_) *δ* 10.31 (s, 1 H), 7.37–7.35 (m, 1 H), 7.18–6.92 (m, 8 H), 5.35 (s, 1 H), 4.35–4.21 (m, 5 H), 3.13–3.02 (m, 2 H), 2.64–2.56 (m, 1 H), 2.49–2.37 (m, 2 H), 2.26–2.19 (m, 1 H), 1.84–1.76 (m, 2 H); ^13^C NMR (100 MHz, CDCl_3_) *δ* 155.3, 153.6, 145.7, 144.3, 136.8, 129.1, 128.6, 128.2, 126.9, 126.8, 126.7, 121.4, 121.2, 118.6, 118.3, 115.0, 110.0, 109.6, 64.5, 64.4, 60.5, 58.4, 50.6, 50.0, 48.8, 29.5, 29.3, 21.2, 14.3, 13.8; C_26_H_25_N_7_O_3_S HRMS (ESI) m/z calculated for [M + H]^+^  = 516.18123, found 516.18154.

*1‐(1‐{[1‐(2*,*3‐dihydro‐1*,*4‐benzodioxin‐6‐yl)‐1 H‐1*,*2*,*3*,*4‐tetrazol‐5‐yl](furan‐3‐yl)methyl}piperidin‐4‐yl)‐2*,*3‐dihydro‐1 H‐1*,*3‐benzodiazol‐2‐one (****9n****)*. White solid. Yield: 68%. ^1^H NMR (400 MHz, CDCl_3_) δ 9.69 (s, 1 H), 7.66–7.39 (m, 2 H), 7.25–7.15 (m, 2 H), 7.14–7.00 (m, 5 H), 6.67 (dd, *J* = 1.9, 0.9 Hz, 1 H), 5.05 (s, 1 H), 4.41–4.30 (m, 4 H), 4.30–4.18 (m, 1 H), 3.04 (dd, *J* = 40.0, 11.3 Hz, 2 H), 2.71–2.04 (m, 4 H), 1.92–1.72 (m, 2 H). ^13^C NMR (101 MHz, CDCl_3_) δ 154.90, 153.74, 145.45, 144.08, 143.33, 142.36, 129.11, 127.97, 127.00, 121.21, 121.05, 118.44, 118.12, 118.10, 114.81, 111.53, 109.68, 109.47, 64.44, 64.37, 54.67, 50.54, 50.33, 47.81, 29.50, 29.16; C_26_H_25_N_7_O_4_ HRMS (ESI) m/z calculated for [M + H]^+^  = 500.19680, found 500.20514.

*1‐(1‐{[1‐(2*,*3‐dihydro‐1*,*4‐benzodioxin‐6‐yl)‐1 H‐1*,*2*,*3*,*4‐tetrazol‐5‐yl](thiophen‐3‐yl)methyl}piperidin‐4‐yl)‐2*,*3‐dihydro‐1 H‐1*,*3‐benzodiazol‐2‐one (****9o****)*. White solid. Yield: 76%. ^1^H NMR (400 MHz, CDCl_3_) δ 9.82 (s, 1 H), 7.42–7.26 (m, 3 H), 7.25–7.15 (m, 1 H), 7.16–7.00 (m, 5 H), 6.93 (dd, *J* = 8.6, 2.5 Hz, 1 H), 5.17 (d, *J* = 0.5 Hz, 1 H), 4.42–4.32 (m, 4 H), 4.25 (tt, *J* = 12.2, 4.2 Hz, 1 H), 3.52 (d, *J* = 4.9 Hz, 1 H), 3.18–3.10 (m, 1 H), 3.04–2.96 (m, 1 H), 2.62–2.34 (m, 3 H), 2.23–2.12 (m, 1 H), 1.86–1.78 (m, 2 H). ^13^C NMR (101 MHz, CDCl_3_) δ 154.97, 154.08, 145.49, 144.08, 134.78, 129.09, 128.44, 128.00, 126.83, 126.10, 125.46, 121.21, 121.04, 118.60, 118.10, 114.95, 109.70, 109.52, 64.43, 64.35, 58.57, 50.57, 50.34, 48.79, 29.48, 29.23; C_26_H_25_N_7_O_3_S HRMS (ESI) m/z calculated for [M + H]^+^  = 516.17396, found 516.18240.

*1‐{1‐[(1‐benzyl‐1* *H‐1*,*2*,*3*,*4‐tetrazol‐5‐yl)(thiophen‐3‐yl)methyl]piperidin‐4‐yl}‐2*,*3‐dihydro‐1 H‐1*,*3‐benzodiazol‐2‐one (****9p****)*. White solid. Yield: 82%. ^1^H NMR (400 MHz, DMSO-*d*_6_) δ 10.81 (s, 1 H), 7.55 (d, *J* = 4.5 Hz, 2 H), 7.46–7.16 (m, 6 H), 7.16–6.79 (m, 4 H), 6.01–5.76 (m, 2 H), 5.63 (s, 1 H), 3.97 (m, 1 H), 3.00 (d, *J* = 6.9 Hz, 1 H), 2.89 (d, *J* = 10.8 Hz, 1 H), 2.37–1.93 (m, 4 H), 1.68–1.45 (m, 2 H). ^13^C NMR (101 MHz, DMSO-*d*_6_) δ 154.22, 153.64, 134.86, 134.37, 129.10, 128.80, 128.74, 128.25, 128.15, 127.72, 125.88, 125.66, 120.48, 120.25, 108.76, 108.62, 79.16, 57.27, 54.88, 50.29, 49.84, 48.62, 47.89, 28.79, 28.52; C_25_H_25_N_7_OS HRMS (ESI) m/z calculated for [M + H]^+^  = 472.18413, found 472.19237.

*1*‐*{1‐[(1‐cyclohexyl‐1 H‐1*,*2*,*3*,*4‐tetrazol‐5‐yl)(thiophen‐3‐yl)methyl]piperidin‐4‐yl}‐2*,*3‐dihydro‐1 H‐1*,*3‐benzodiazol‐2‐one (****9q****)*. White solid. Yield: 65%. ^1^H NMR (400 MHz, DMSO-*d*_6_) δ 10.79 (s, 1 H), 7.71–7.44 (m, 2 H), 7.27 (dd, *J* = 4.8, 1.4 Hz, 1 H), 7.22–7.06 (m, 1 H), 7.06–6.79 (m, 3 H), 5.66 (s, 1 H), 4.03 (m, 1 H), 3.04 (d, *J* = 10.8 Hz, 1 H), 2.87 (d, *J* = 10.8 Hz, 1 H), 2.46–2.15 (m, 3 H), 2.16–2.00 (m, 2 H), 2.00–1.77 (m, 5 H), 1.77–1.38 (m, 5 H), 1.38–1.20 (m, 1 H). ^13^C NMR (101 MHz, DMSO-*d*_6_) δ 153.63, 153.24, 134.77, 129.21, 128.67, 128.32, 125.92, 125.41, 120.48, 120.20, 108.81, 108.34, 79.16, 57.51, 57.12, 50.29, 49.76, 47.93, 32.87, 32.60, 28.91, 28.63, 24.85, 24.82, 24.63; C_24_H_29_N_7_OS HRMS (ESI) m/z calculated for [M + H]^+^  = 464.21543, found 464.22364.

*1‐[1‐({1‐[(4‐methylbenzenesulfonyl)methyl]‐1* *H‐1*,*2*,*3*,*4‐tetrazol‐5‐yl}(thiophen‐3‐yl)methyl)piperidin‐4‐yl]‐2*,*3‐dihydro‐1* *H‐1*,*3‐benzodiazol‐2‐one (****9r****)*. White solid. Yield: 53%. ^1^H NMR (400 MHz, DMSO-*d*_6_) δ 10.79 (s, 1 H), 7.73–7.62 (m, 2 H), 7.58 (dd, *J* = 5.0, 2.9 Hz, 1 H), 7.55–7.42 (m, 3 H), 7.25–7.14 (m, 2 H), 7.04–6.86 (m, 3 H), 6.72–6.50 (m, 2 H), 5.47 (s, 1 H), 4.04–3.87 (m, 1 H), 2.92 (dd, *J* = 53.6, 11.2 Hz, 2 H), 2.42 (s, 3 H), 2.39–2.14 (m, 3 H), 2.03–1.84 (m, 1 H), 1.62 (dd, *J* = 31.7, 11.8 Hz, 2 H). ^13^C NMR (101 MHz, DMSO) δ 154.84, 153.64, 146.08, 133.76, 133.12, 130.24, 129.19, 128.72, 128.27, 125.80, 125.77, 120.50, 120.28, 108.74, 108.61, 64.73, 57.23, 49.82, 49.71, 47.81, 28.86, 28.56, 21.22; C_26_H_27_N_7_O_3_S_2_ HRMS (ESI) m/z calculated for [M + H]^+^  = 550.16168, found 550.17011.

*1‐(1‐{[1‐(2*,*6‐dimethylphenyl)‐1 H‐1*,*2*,*3*,*4‐tetrazol‐5‐yl](thiophen‐3‐yl)methyl}piperidin‐4‐yl)‐2*,*3‐dihydro‐1 H‐1*,*3‐benzodiazol‐2‐one (****9 s****)*. White solid. Yield: 72%. ^1^H NMR (400 MHz, DMSO-*d*_6_) δ 10.78 (s, 1 H), 7.55 (dd, *J* = 5.0, 3.0 Hz, 1 H), 7.50 (t, *J* = 7.6 Hz, 1 H), 7.44 (dd, *J* = 3.0, 1.3 Hz, 1 H), 7.41 (m, 1 H), 7.29 (m, 1 H), 7.21 (dd, *J* = 5.0, 1.3 Hz, 1 H), 7.08 (m, 1 H), 7.02– 6.92 (m, 3 H), 4.87 (s, 1 H), 3.98 (m, 1 H), 3.10–2.72 (m, 2 H), 2.39–2.11 (m, 3 H), 2.06 (s, 3 H), 2.04–1.90 (m, 1 H), 1.70–1.53 (m, 2 H), 1.48 (s, 3 H). ^13^C NMR (101 MHz, DMSO) δ 155.34, 153.61, 135.41, 135.35, 134.17, 131.42, 131.05, 129.23, 128.90, 128.79, 128.52, 128.25, 126.45, 126.10, 120.46, 120.28, 108.76, 108.34, 79.16, 57.98, 51.00, 49.84, 48.14, 28.67, 28.41, 17.15, 16.28; C_26_H_27_N_7_OS HRMS (ESI) m/z calculated for [M + H]^+^  = 486.19978, found 486.20824.

*1‐{1‐[(1‐cyclopentyl‐1* *H‐1*,*2*,*3*,*4‐tetrazol‐5‐yl)(naphthalen‐1‐yl)methyl]piperidin‐4‐yl}‐2*,*3‐dihydro‐1* *H‐1*,*3‐benzodiazol‐2‐one* (***9t***). White solid. Yield: 62%. ^1^H NMR (400 MHz, CDCl_3_) δ 9.66 (s, 1 H), 8.53–8.50 (m, 1 H), 7.91–7.85 (m, 2 H), 7.65–7.61 (m, 1 H), 7.58–7.53 (m, 2 H), 7.49–7.45 (m, 1 H), 7.22–7.20 (m, 1 H), 7.12–7.05 (m, 3 H), 5.93 (s, 1 H), 4.91–4.83 (m, 1 H), 4.40–4.33 (m, 1 H), 3.27–3.23 (m, 1 H), 2.92–2.88 (m, 1 H), 2.81–2.76 (m, 1 H), 2.72–2.59 (m,1 H), 2.53–2.44 (m, 1 H), 1.92–1.78 (m, 8 H), 1.64–1.59 (m, 2 H); ^13^C NMR (101 MHz, CDCl_3_) δ 155.1, 153.5, 134.3, 131.7, 131.5, 129.7, 129.4, 129.1, 128.1, 127.0, 126.6, 126.3, 125.1, 123.7, 121.4, 121.2, 109.8, 109.4, 61.3, 59.3, 52.0, 51.2, 50.0, 33.5, 33.3, 29.6, 24.9, 24.7; C_29_H_31_N_7_O HRMS (ESI) m/z calculated for [M^ + ^H] ^+^  = 494.26629, found 494.26652.

*1‐{1‐[(1‐benzyl‐1* *H‐1*,*2*,*3*,*4‐tetrazol‐5‐yl)[2‐(trifluoromethoxy)phenyl]methyl]piperidin‐4‐yl}‐2*,*3‐dihydro‐1* *H‐1*,*3‐benzodiazol‐2‐one* (***9u***). White solid. Yield: 79%. ^1^H NMR (400 MHz, CDCl_3_) δ 10.15 (s, 1 H), 7.89–7.86 (m, 1 H), 7.40–7.31 (m, 5 H), 7.28–7.26 (m, 1 H), 7.22–7.19 (m, 2 H), 7.12–7.01 (m, 4 H), 5.78 (d, J = 15.3 Hz, 1 H), 5.56 (d, J = 15.3 Hz, 1 H), 5.36 (s, 1 H), 4.19–4.12 (m, 1 H), 2.88–2.85 (m, 1 H), 2.76–2.75 (m, 1 H), 2.46–2.29 (m, 2 H), 2.19–2.12 (m, 2 H), 1.73–1.67 (m, 2 H); ^13^C NMR (101 MHz, CDCl_3_) δ 155.3, 154.0, 147.6, 133.3, 131.9, 130.2, 129.3, 129.2, 129.1, 128.2, 127.6, 126.9, 126.2, 121.8, 121.4, 121.2, 119.8, 109.9, 109.5, 55.8, 51.5, 50.6, 50.5, 49.3, 29.4, 29.3; C_28_H_26_F_3_N_7_O_2_ HRMS (ESI) m/z calculated for [M + H]^+^  = 550.21728, found 550.21728.

*1‐{1‐[(S)‐{1‐[(1 S)‐1‐phenylethyl]‐1 H‐1,2,3,4‐tetrazol‐5‐yl}(thiophen‐3‐yl)methyl]piperidin‐4‐yl}‐2,3‐dihydro‐1 H‐1,3‐benzodiazol‐2‐one (****12a****)*. White solid. Yield: 35%. ^1^H NMR (400 MHz, CDCl_3_) δ 10.53 (s, 1 H), 7.46–7.34 (m, 4 H), 7.34–7.25 (m, 3 H), 7.22 (dd, *J* = 5.0, 1.3 Hz, 1 H), 7.17–7.08 (m, 1 H), 7.07–7.01 (m, 3 H), 5.73 (q, *J* = 7.0 Hz, 1 H), 5.15 (s, 1 H), 4.26–4.14 (m, 1 H), 3.06–2.73 (m, 2 H), 5.53–2.32 (m, 2 H), 2.28–2.07 (m, 2 H), 2.00 (d, *J* = 7.0 Hz, 3 H), 1.74 (d, *J* = 11.6 Hz, 1 H), 1.70–1.52 (d, *J* = 11.6 Hz, 1 H). ^13^C NMR (101 MHz, CDCl_3_) δ 155.30, 139.51, 129.20, 128.99, 128.70, 128.49, 128.17, 126.40, 126.31, 121.29, 120.97, 109.91, 109.61, 59.39, 58.55, 50.84, 50.52, 48.91, 29.39, 28.98, 22.69; C_26_H_27_N_7_OS HRMS (ESI) m/z calculated for [M + H]^+^  = 486.19978, found 486.20677.

*1‐{1‐[(R)‐{1‐[(1* *S)‐1‐phenylethyl]‐1* *H‐1*,*2*,*3*,*4‐tetrazol‐5‐yl}(thiophen‐3‐yl)methyl]piperidin‐4‐yl}‐2*,*3‐dihydro‐1* *H‐1*,*3‐benzodiazol‐2‐one* (***12b***). White solid. Yield: 31%. ^1^H NMR (400 MHz, CDCl_3_) δ 10.57 (s, 1 H), 7.39–7.24 (m, 6 H), 7.22 (dd, J = 2.9, 1.3 Hz, 1 H), 7.20–7.10 (m, 3 H), 7.10–7.00 (m, 2 H), 6.15 (q, J = 6.8 Hz, 1 H), 5.31–5.01 (m, 1 H), 4.32–4.13 (m, 1 H), 3.25–3.01 (m, 1 H), 2.95–2.74 (m, 1 H), 2.63–2.35 (m, 3 H), 2.31–2.16 (m, 1 H), 2.02 (d, J = 7.0 Hz, 3 H), 1.80 (dd, J = 23.7, 10.9 Hz, 2 H). ^13^C NMR (101 MHz, CDCl_3_) δ 155.32, 139.26, 129.15, 129.11, 128.69, 128.26, 128.22, 128.18, 126.42, 126.14, 121.42, 121.07, 110.00, 109.24, 59.81, 58.91, 51.48, 50.48, 49.32, 29.14, 22.62; C_26_H_27_N_7_OS HRMS (ESI) m/z calculated for [M + H]^+^  = 486.19978, found 486.20677. Compound **12b** was crystalized by solvent evaporation in ethyl acetate and chloroform. The X-ray crystal structure of **12b** was deposited in Cambridge Crystallographic Data Centre and the deposition number is CCDC 1573497.

#### Plaque assay

The plaque reduction assay was performed as previously reported^38^, except MDCK cells expressing ST6Gal I were used instead of regular MDCK cells^39^. Briefly, the confluent monolayers of ST6Gal MDCK cells were incubated with ~100 pfu virus samples in DMEM with 0.5% BSA for 1 h at 4 °C, then 37 °C for 1 h. The inoculums were removed, and the cells were washed with phosphate buffered saline (PBS). The cells were then overlaid with DMEM containing 1.2% Avicel microcrystalline cellulose (FMC BioPolymer, Philadelphia, PA) and NAT (2.0 µg/mL). To examine the effect of the compounds on plaque formation, the overlay media was supplemented with compounds at testing concentrations. At two days after infection, the monolayers were fixed and stained with crystal violet dye solution (0.2% crystal violet, 20% methanol). Influenza A virus A/WSN/33 (H1N1) was obtained from Dr. Robert Lamb at the Northwestern University. The influenza viruses A/Texas/04/2009 (H1N1), B/Wisconsin/1/2010, and B/Brisbane/60/2008 were obtained from Dr. James Noah at the Southern Research Institute. Influenza A and B viruses A/Switzerland/9715293/2013 × −247 (H3N2), FR-1366, A/Washington/29/2009 (H1N1), FR-460, A/California/07/2009 (H1N1), FR-201, A/Washington/29/2009 (H1N1), FR-460, B/Memphis/20/1996, FR-486, B/Utah/9/2014, FR-1372, and B/Phuket/3073/2013, FR-1364, were obtained through the Influenza Reagent Resource, Influenza Division, WHO Collaborating Center for Surveillance, Epidemiology and Control of Influenza, Centers for Disease Control and Prevention, Atlanta, GA, USA. The influenza viruses A/Denmark/524/2009 (H1N1) and A/Denmark/528/2009 (H1N1) was obtained from Dr. Elena Govorkova at St. Jude Children’s Research Hospital. All influenza strains used in this study are biosafety level 2 (BSL-2) pathogens and all experiments using influenza viruses were performed in BSL-2 certified laboratory.

#### Cytotoxicity assay

Evaluation of the cytotoxicity of compounds was carried out using the neutral red uptake assay^40^. Briefly, 80,000 cells/mL of MDCK or A549 cells in DMEM medium supplemented with 10% FBS and 100 U/mL Penicillin-Streptomycin were dispensed into 96-well cell culture plates at 100 µL/well. Twenty-four hours later, the growth medium was removed and washed with 100 µL PBS buffer; then for the cytotoxicity assay, 200 µL fresh DMEM (no FBS) medium containing serial diluted compounds was added to each well. After incubating for 48 h at 37 °C with 5% CO_2_ in a CO_2_ incubator, the medium was removed and replaced with 100 µL DMEM medium containing 40 µg/mL neutral red for four hours at 37 °C. The amount of neutral red uptake was determined at absorbance 540 nm using a Multiskan FC Microplate Photometer (Fisher Scientific). The CC_50_ values were calculated from best-fit dose response curves with variable slope in GraphPad Prism version 5.

#### ELISA assay

To test the inhibitory activity of compound on PA_C_–PB1_N_ interaction, ELISA was performed^22^. Briefly, microtiter plates were coated with 400 ng of His-tagged PA_239-716_ (PA_C_) for 3 h at 37 °C, followed by blocking with 2% (wt/vol) BSA in phosphate buffer saline (PBS) for 1 h. After washing with PBS containing 0.3% Tween 20, plates were incubated with 200 ng of GST-tagged PB1_1-25_ (PB1_N_) protein and compounds overnight at room temperature. Then the PA_C_–PB1_N_ interaction was detected using a horseradish peroxidase (HRP)-conjugated anti-GST monoclonal antibody and its chromogenic substrate TMB. The absorbance at 450 nm was read on a plate reader. The IC_50_ values were calculated from best-fit dose response curves with variable slope in GraphPad Prism version 5.

#### Time-of-Addition Experiment

A time-of-addition experiment was performed according to the procedure described earlier^29,41,42^. Briefly, MDCK cells were seeded at 6 cm^2^ dishes at a 2 × 10^5^ cells/dish cell density. After incubating for 24 h to allow cells to attach, the cells were infected with A/WSN/33 (H1N1) virus at a MOI of 0.01 at −2 h time point. Antiviral compounds, such as oseltamivir carboxylate (1 µM) or **12a** (10 µM) was added at different time points before, during, or after viral infection as illustrated in Fig. 4. Viruses were harvested from the cell culture supernatant at 12 h post infection. The virus titers were quantified by plaque assay.

#### RNA Extraction and Real-Time PCR

Total RNA was extracted from influenza A/WSN/33 (H1N1) infected cells using Trizol reagents (Thermo Fisher Scientific). After removing genomic DNA by RQ1 RNase-Free DNase (Promega), the first strand of cDNA was synthesized using 1.2 μg of total RNA and AMV Reverse Transcriptase (Promega). vRNA specific primer (5′-AGCAAAAGCAGG-3′), cRNA specific primer (5′-AGTAGAAACAAGG-3′) or oligo (dT)18 was used for detecting influenza vRNA, cRNA or mRNA, respectively. Real-time PCR was performed on a StepOnePlus Real-Time PCR System (Thermo Fisher Scientific) using FastStart Universal SYBR Green Master (Rox) (Roche, Basel, Switzerland) and following influenza NP-specific primers: NP-F: 5′-AGGGTCAGTTGCTCACAAGTCC-3′; NP-R: 5′-TTTGAAGCAGTCTGAAAGGGTCTA-3′. GAPDH was also amplified to serve as a control using human GAPDH-specific primers (GAPDH-F: 5′-ACACCCACTCCTCCACCTTTG-3′ and GAPDH-R: 5′-CACCACCCTGTTGCTGTAGCC-3′). The amplification conditions were: 95 °C for 10 min; 40 cycles of 15 s at 95 °C and 60 s at 60 °C. Melting curve analysis was performed to verify the specificity of each amplification. All experiments were repeated three times independently.

#### Serial viral passage experiments

Serial drug passage experiments were performed accordingly to previously published protocol^29,30,42^. Briefly, MDCK cells were infected with the A/WSN/33 (H1N1) virus at MOI 0.001 for 1 h. Then the inoculum was removed and MDCK cells were incubated with 1 µM compound **12a** in the first passage and the concentration of **12a** was gradually increased 2-fold in passages 2–7 and kept constant at 64 µM in passages 7–10. In each passage, the viruses were harvested when a significant cytopathic effect was observed, which usually takes 2-3 days after virus infection. The titers of harvested viruses were determined by plaque assay. The drug sensitivity after passages 3, 6, and 10 was determined via plaque assay as described previously^43^. Oseltamivir carboxylate was included as a control and similar fold of drug selection pressure was applied. The drug sensitivity of oseltamivir at passages 3, 6, and 10 was determined via plaque assay.

## Electronic supplementary material

Supplementary information
