# Supplementary material for: Exploring Ugi-Azide Four-Component Reaction Products for Broad-Spectrum Influenza Antivirals with a High Genetic Barrier to Drug Resistance
Source: Sci Rep. 2018 Mar 15;8:4653. doi: 10.1038/s41598-018-22875-9 (PMC5854701; doi:10.1038/s41598-018-22875-9)

Supplementary Information

Exploring Ugi-Azide Four-Component Reaction Products for Broad-Spectrum Influenza Antivirals with a High Genetic Barrier to Drug Resistance

Jiantao Zhang,[†,#] Yanmei Hu,[†,#] Christopher Foley,[§] Yuanxiang Wang,[†] Rami Musharrafieh,[§] Shuting Xu,[§] Yongtao Zhang,[§] Chunlong Ma,[‡] Christopher Hulme,[§,†] Jun Wang*[,†,‡]

[†]Department of Pharmacology and Toxicology, College of Pharmacy, The University of Arizona, Tucson, Arizona 85721, United States

[§]Department of Chemistry and Biochemistry, The University of Arizona, Tucson, Arizona 85721, United States

[‡]BIO5 Institute, The University of Arizona, Tucson, Arizona, 85721, United States

[#]J. Z. and Y. H. contributed equally to this work.

*Corresponding author:

Jun Wang, Tel: 520-626-1366, Fax: 520-626-0749, email: junwang@pharmacy.arizona.edu

^{1}H NMR for **5** ($CDCl_3$)

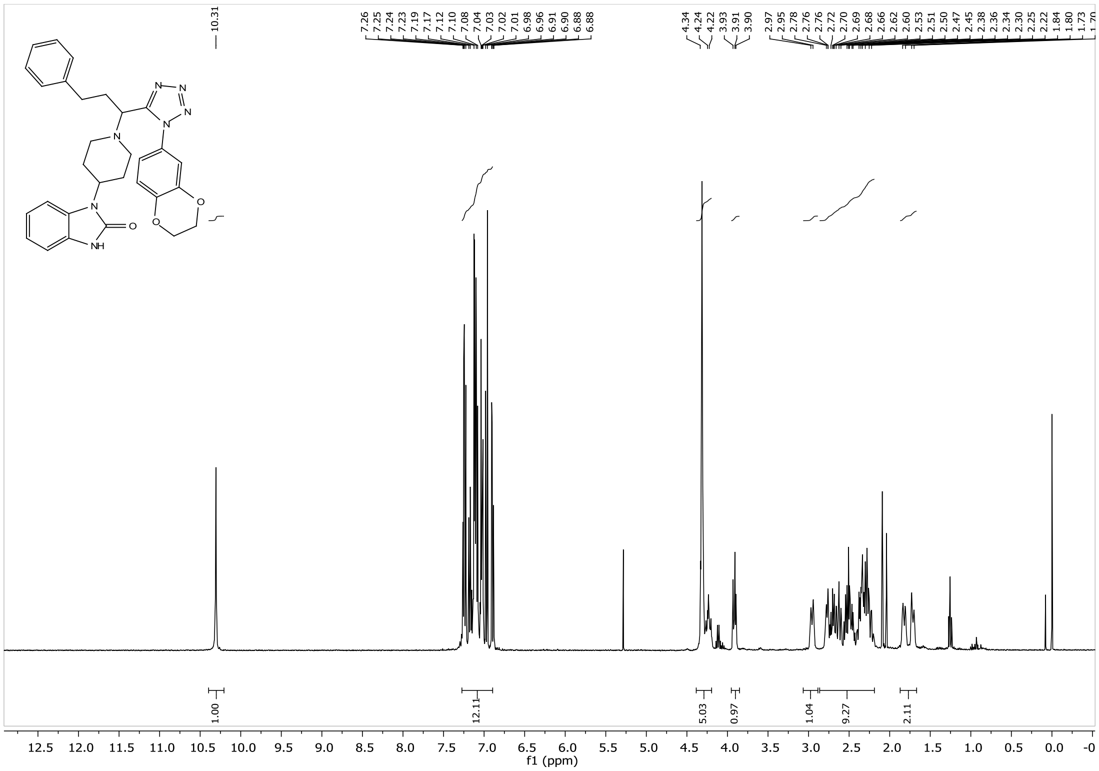

^{13}C NMR for **5** ($CDCl_3$)

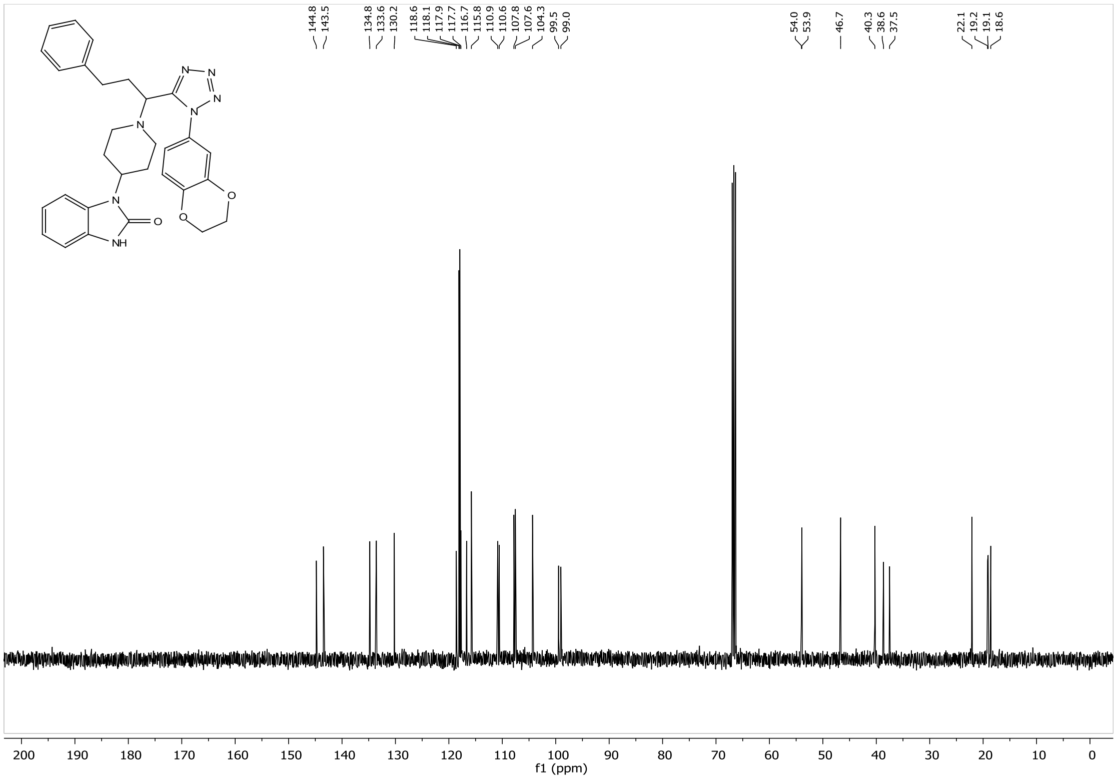

^{1}H NMR for **9a** (DMSO-d$_6$)

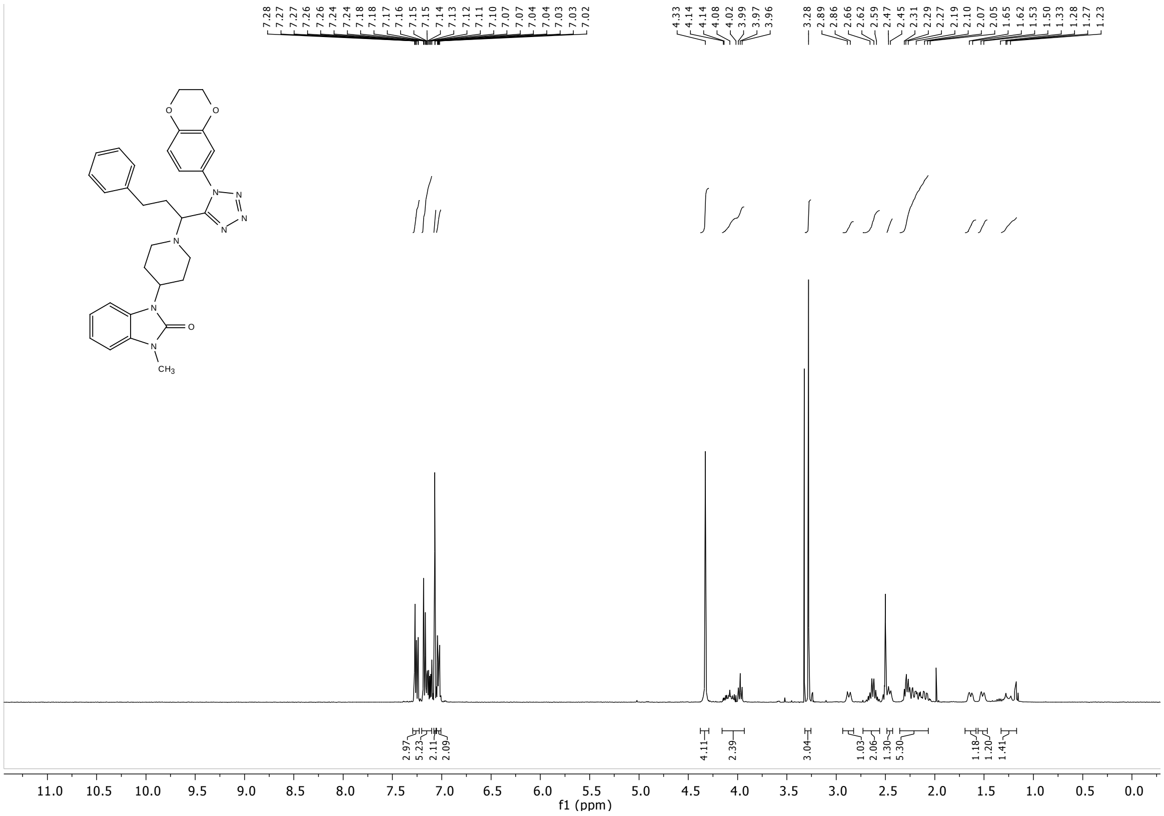

^{13}C NMR for **9a** (DMSO-d$_6$)

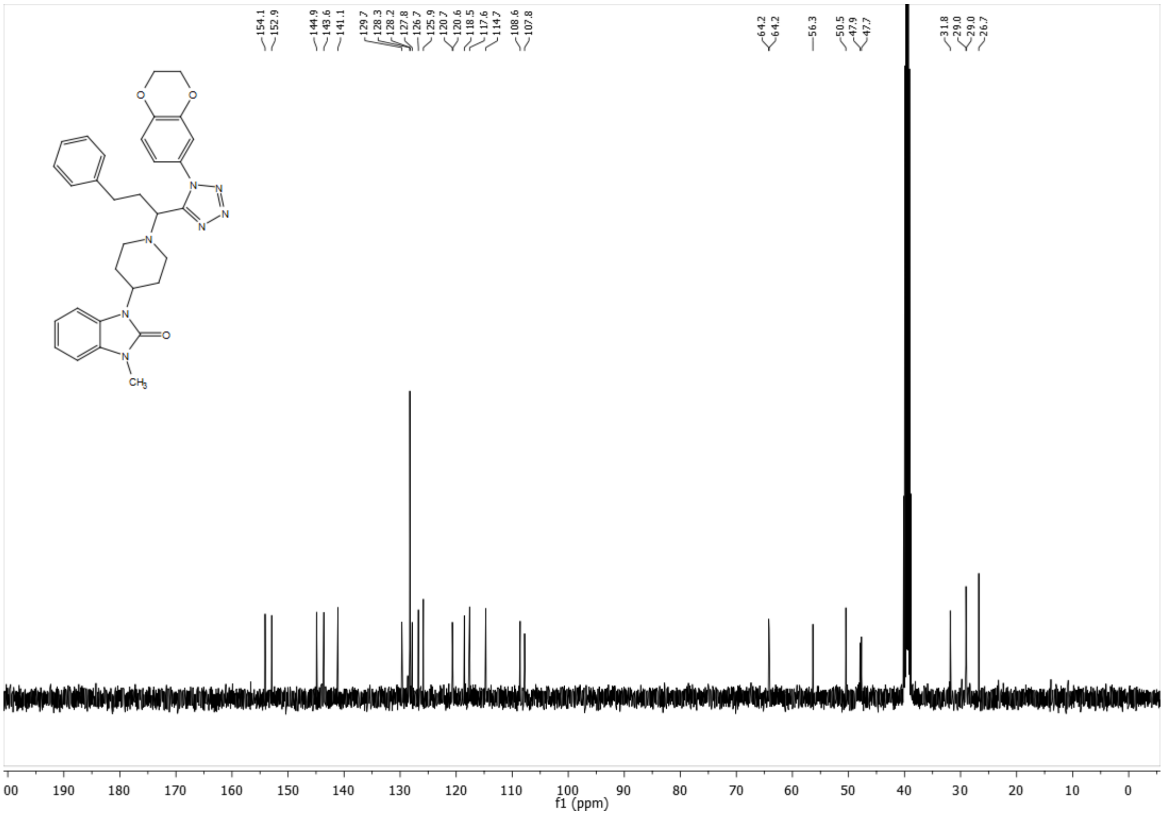

^{1}H NMR for **9b** (DMSO-d_6)

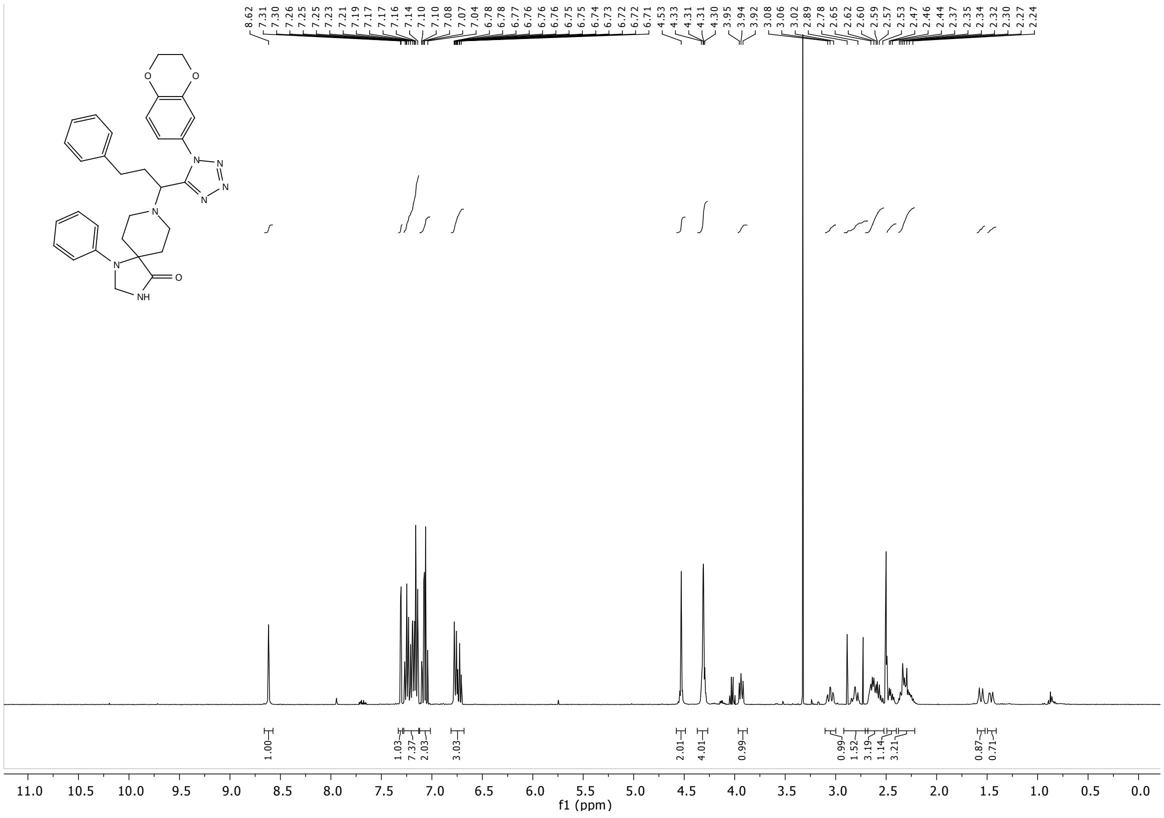

^{13}C NMR for **9b** (DMSO-d_6)

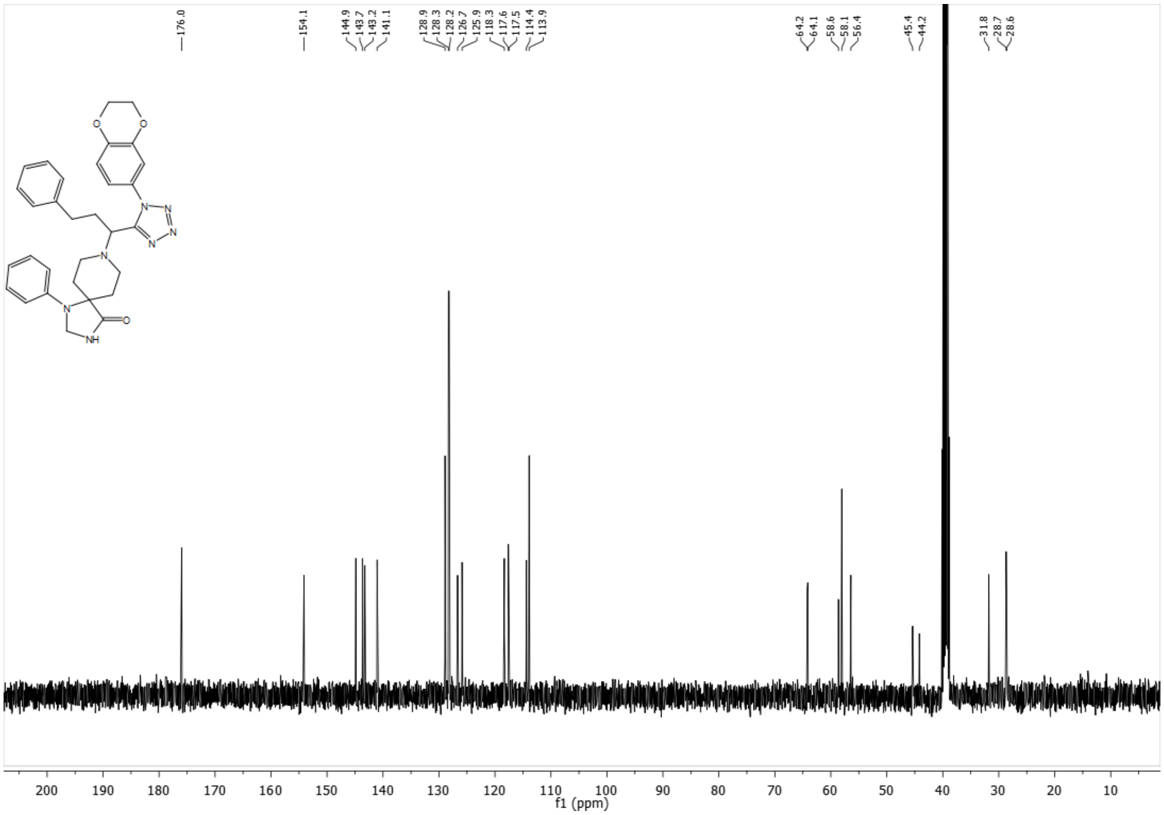

^{1}H NMR for **9c** ($CDCl_3$)

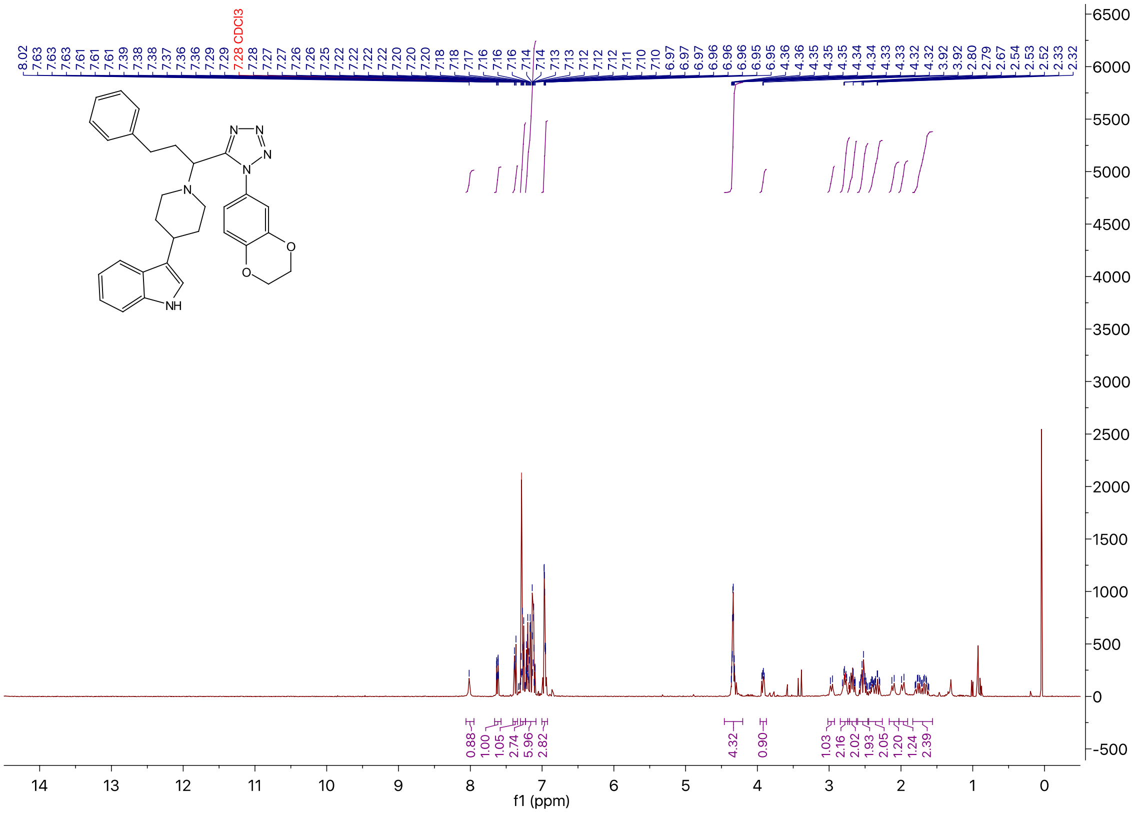

^{13}C NMR for **9c** ($CDCl_3$)

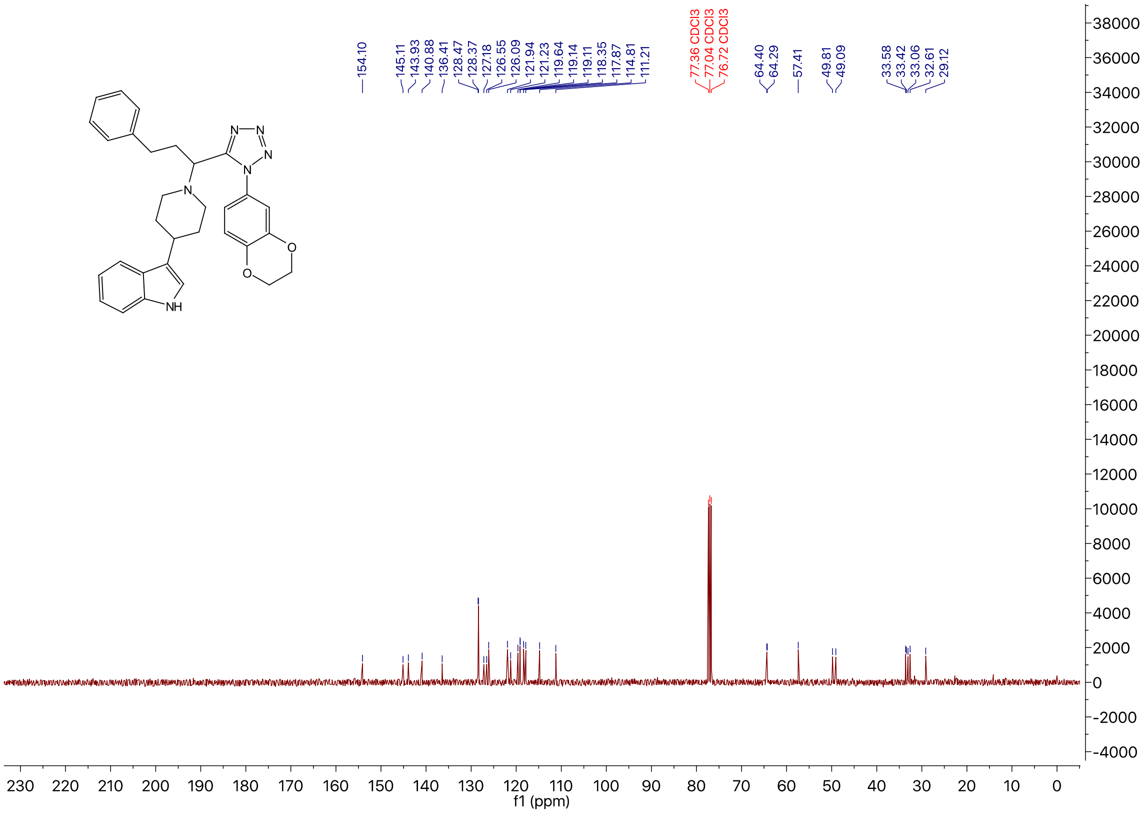

^{1}H NMR for **9d** (DMSO-d$_6$)

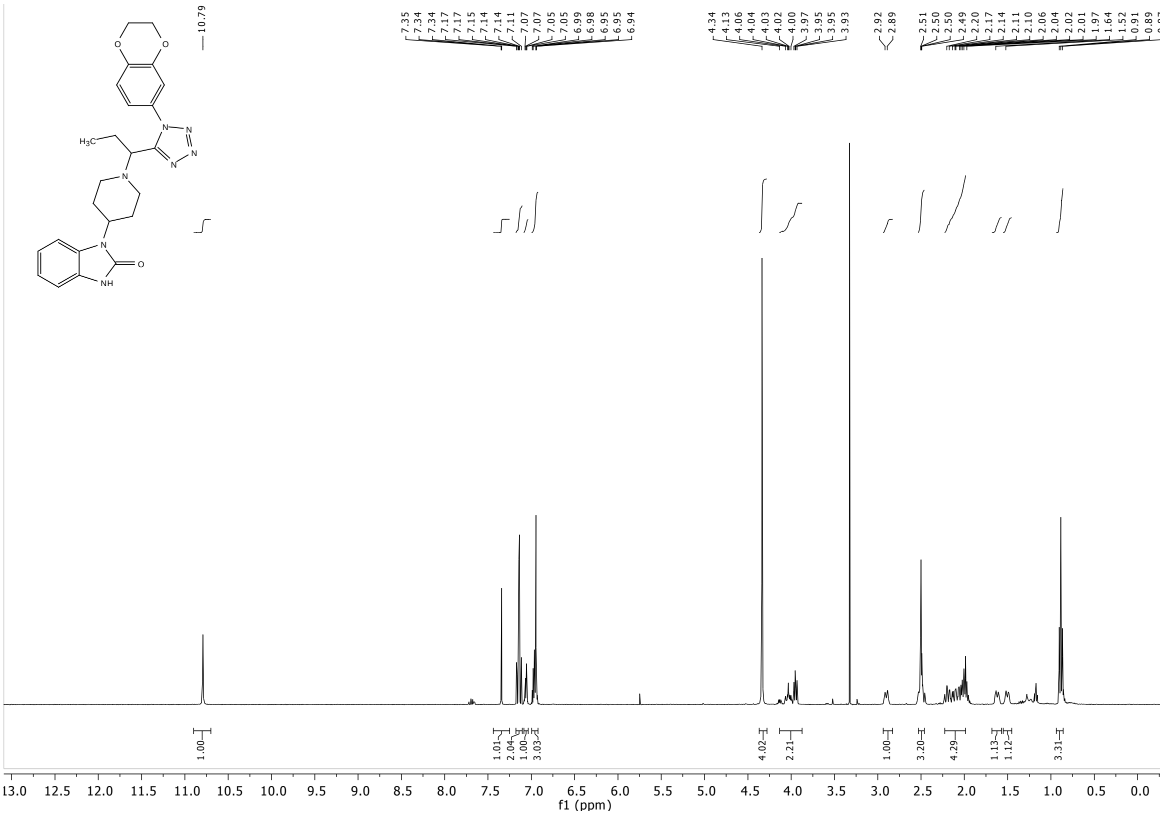

^{13}C NMR for **9d** (DMSO-d$_6$)

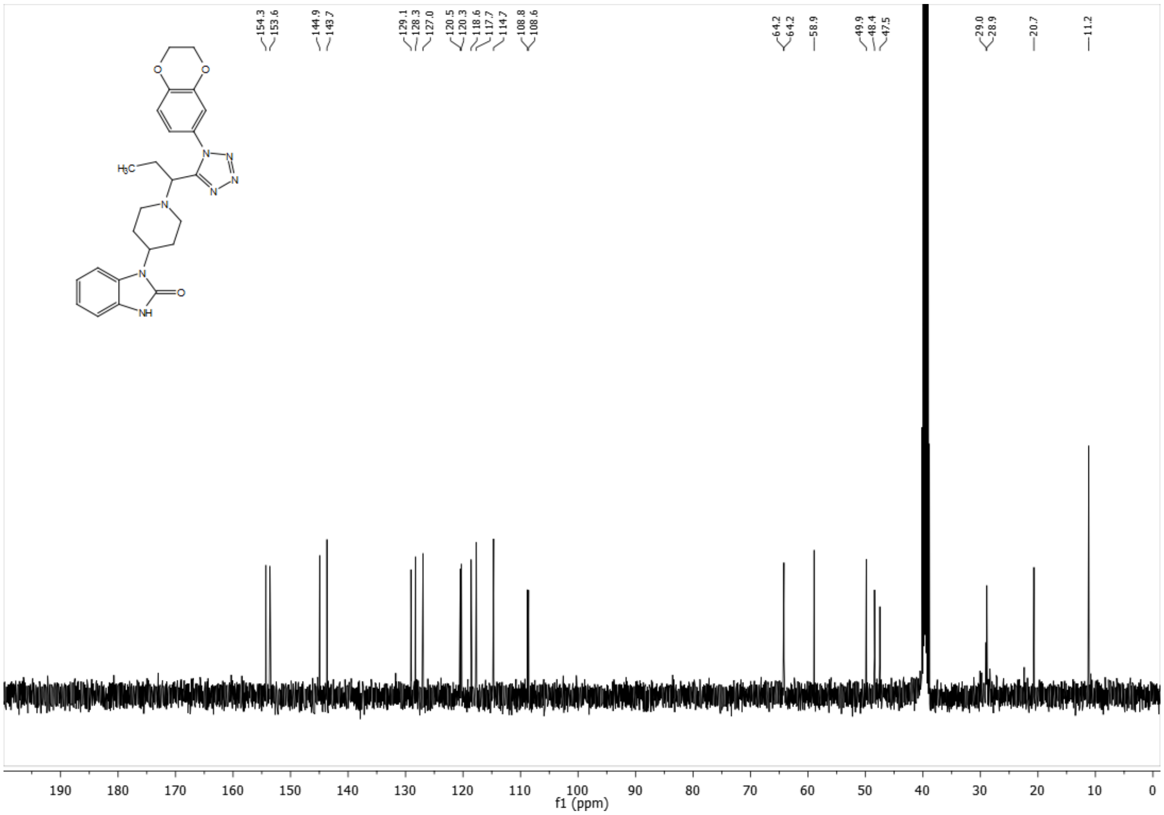

^{1}H NMR for **9e** (DMSO-d_6)

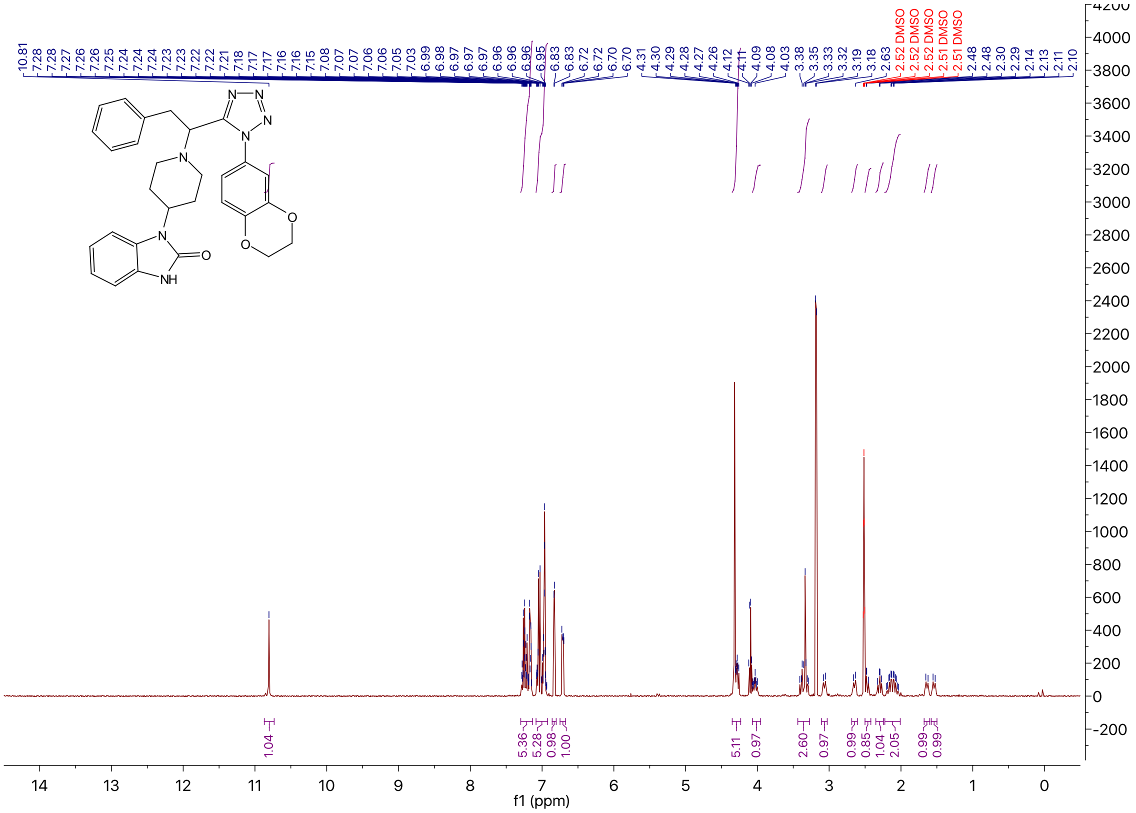

^{13}C NMR for **9e** (DMSO-d_6)

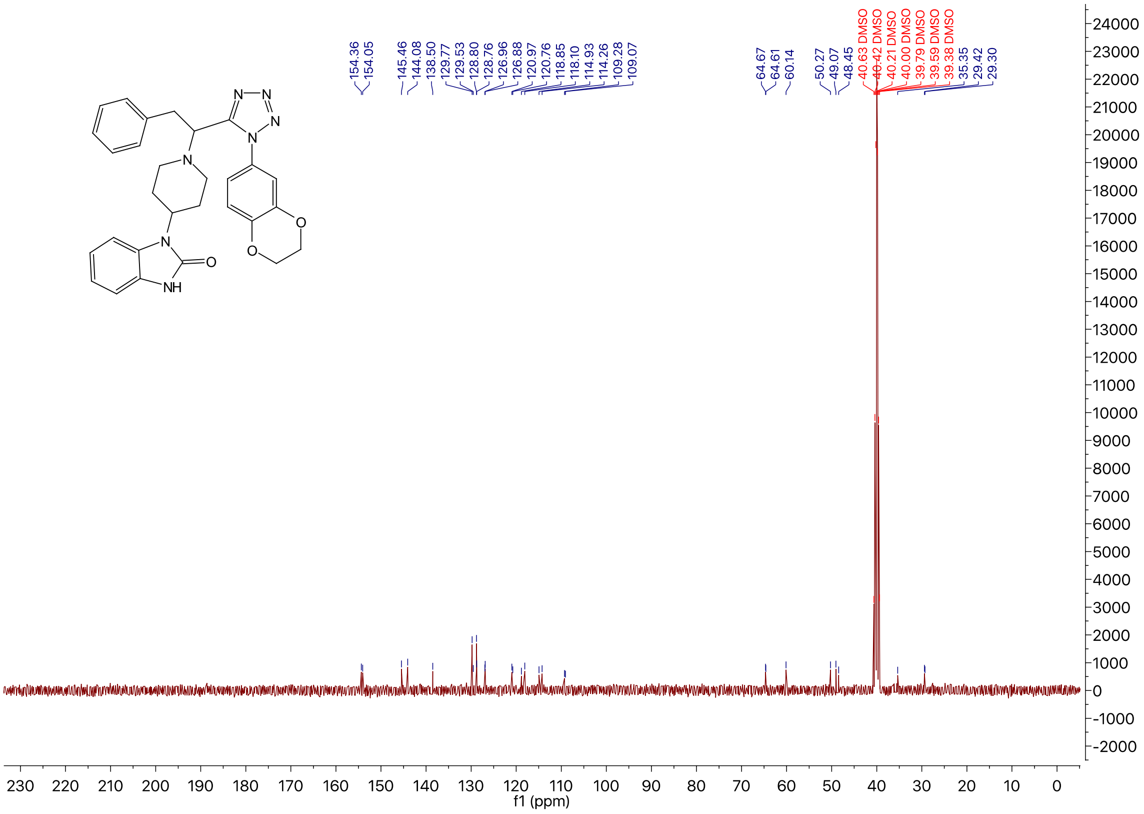

^{1}H NMR for **9f** ((DMSO-d_6)

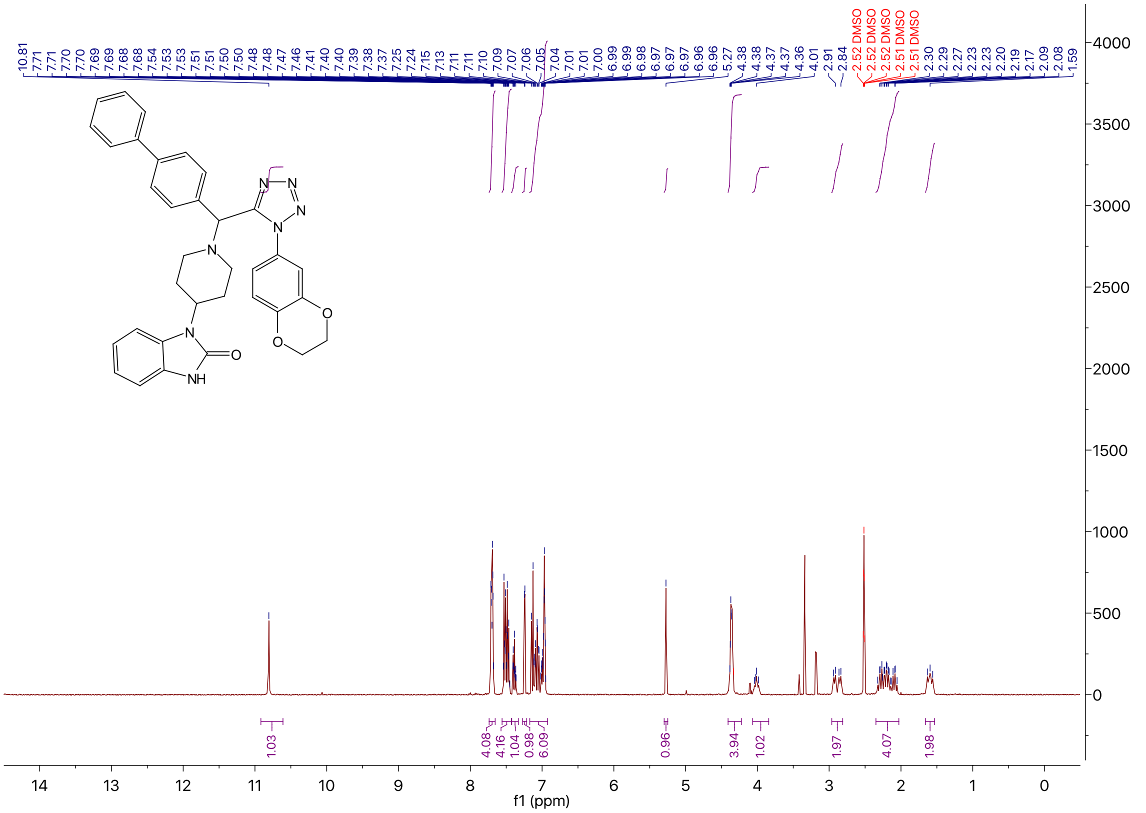

^{13}C NMR for **9f** (DMSO-d_6)

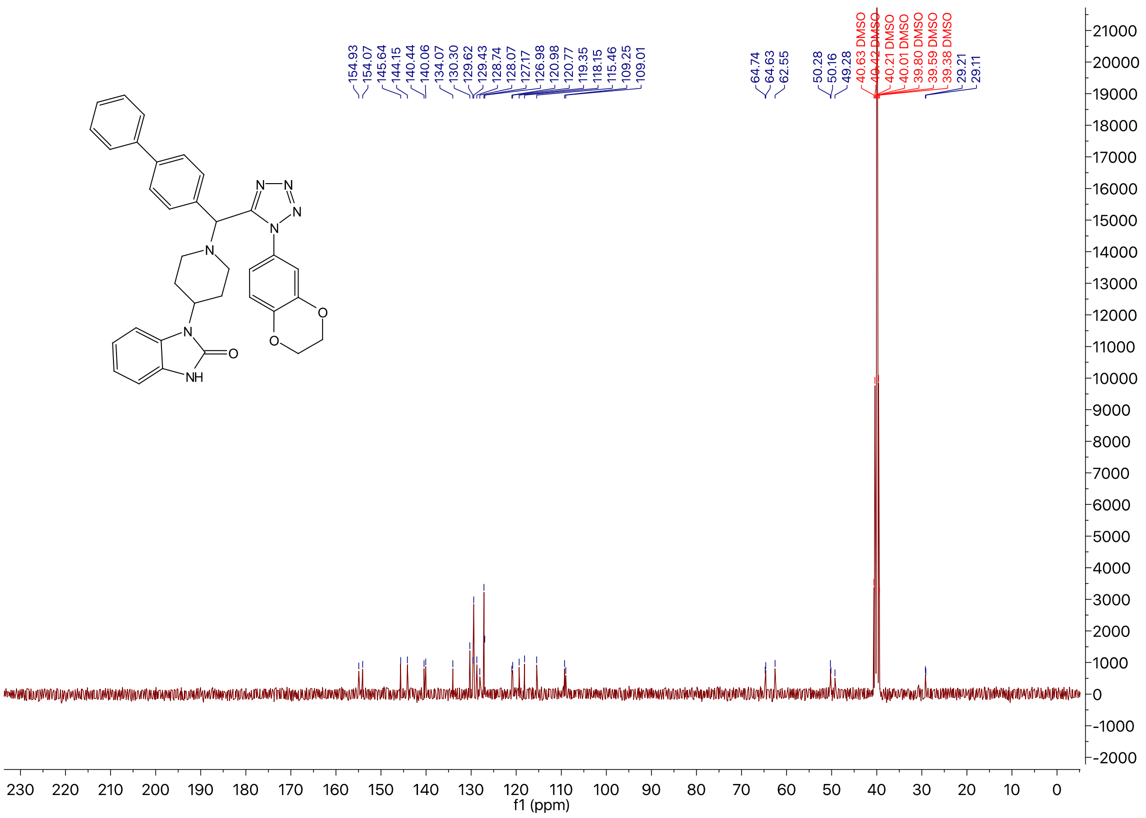

^{1}H NMR for **9g** (DMSO-d$_6$)

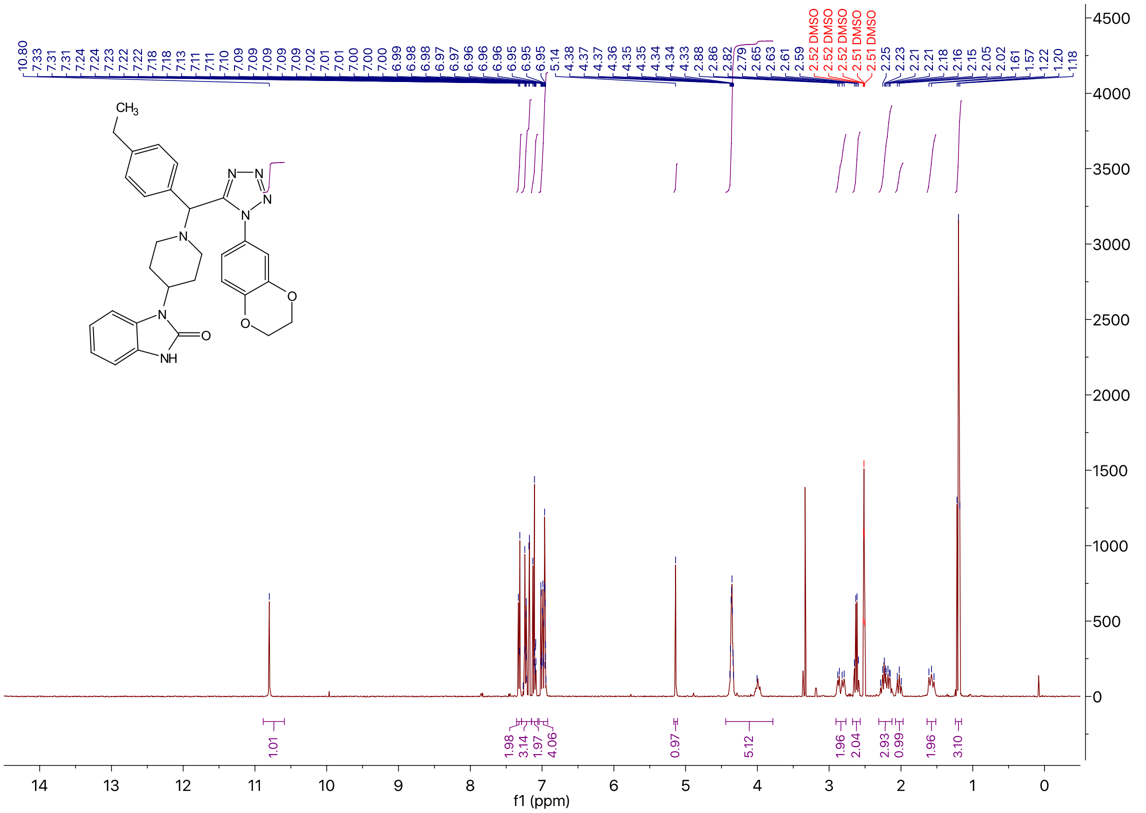

^{13}C NMR for **9g** (DMSO-d$_6$)

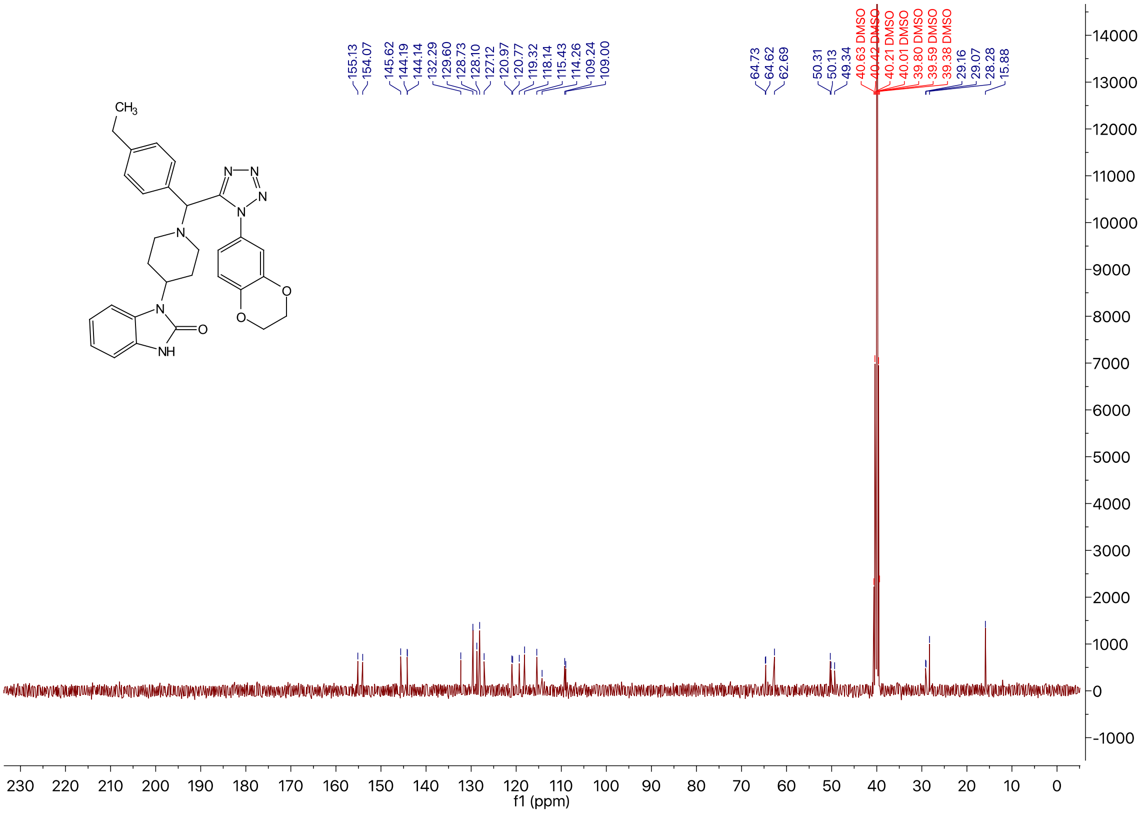

^{1}H NMR for **9h** (DMSO-d$_6$)

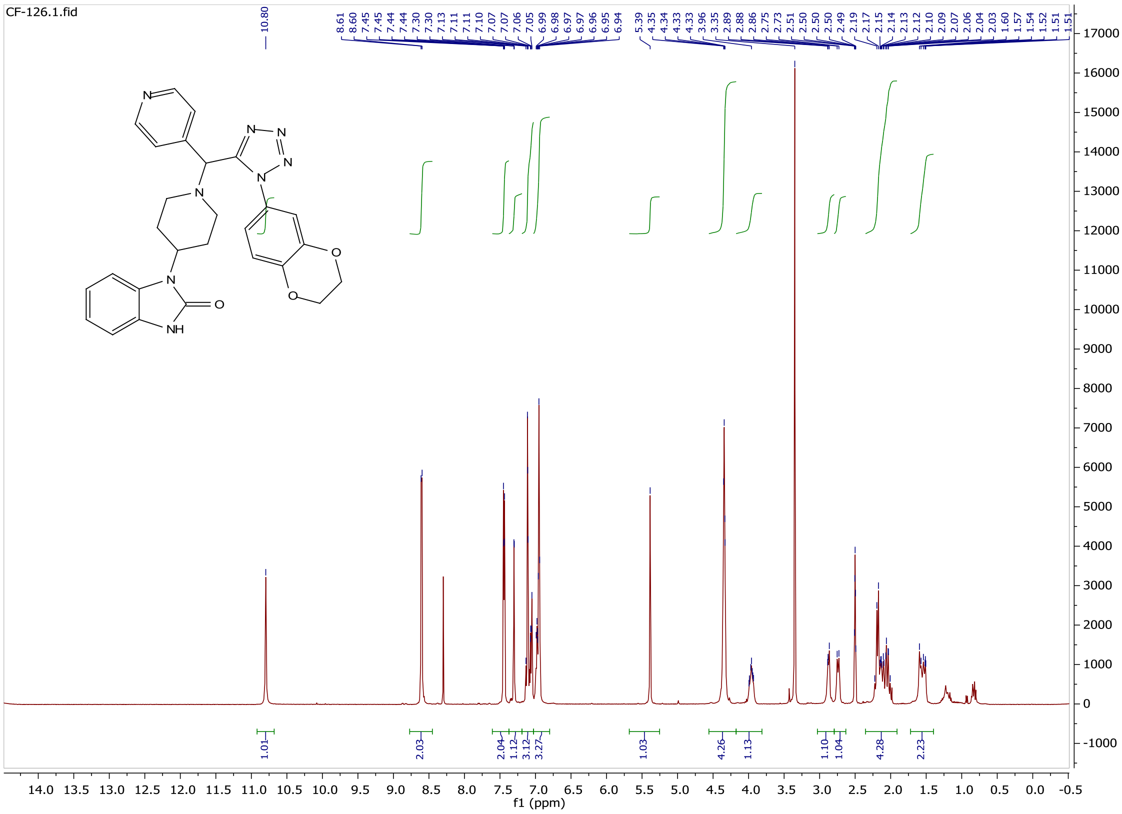

^{13}C NMR for **9h** (DMSO-d$_6$)

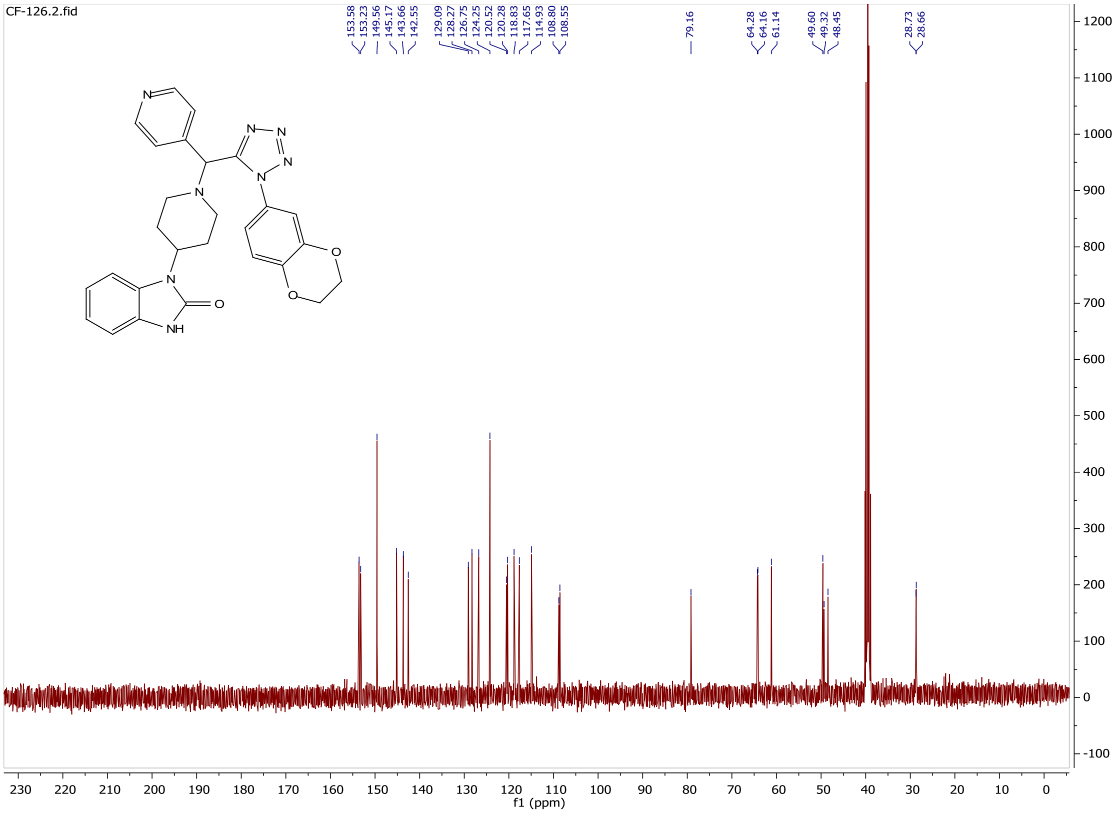

^{1}H NMR for **9i** (DMSO-d_6)

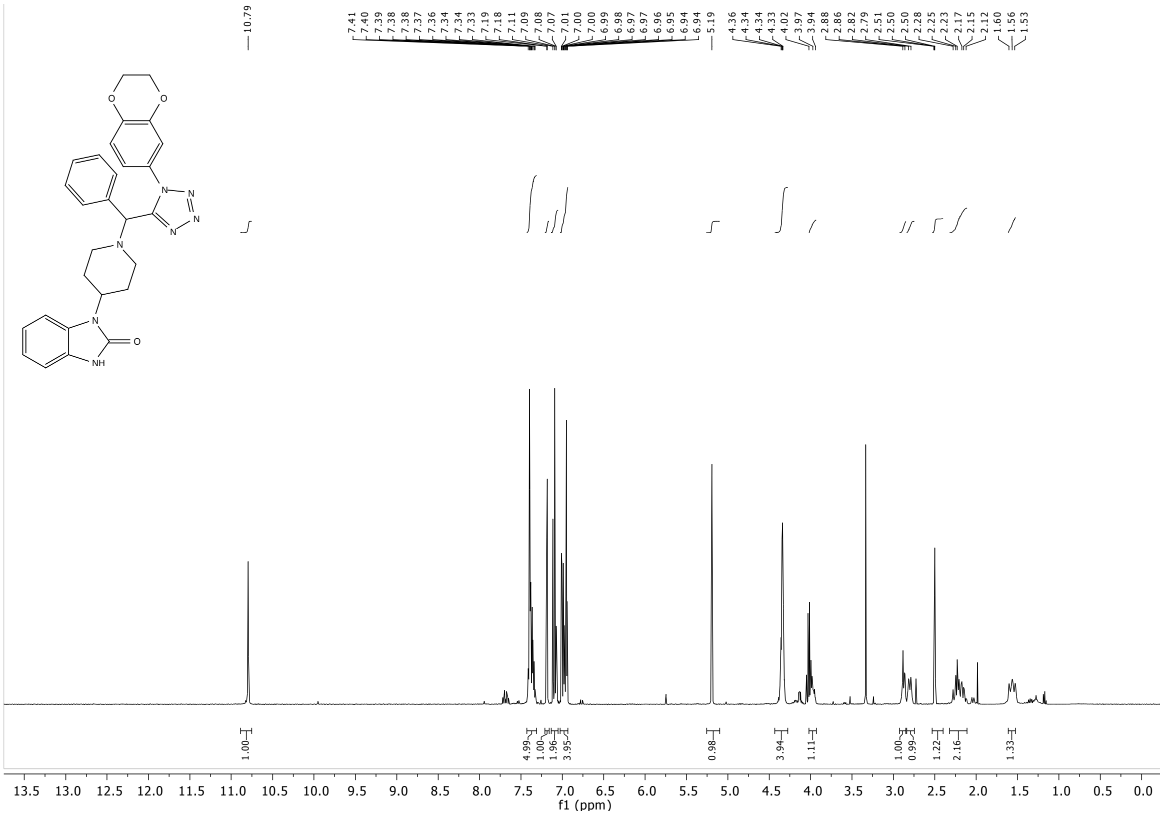

^{13}C NMR for **9i** (DMSO-d_6)

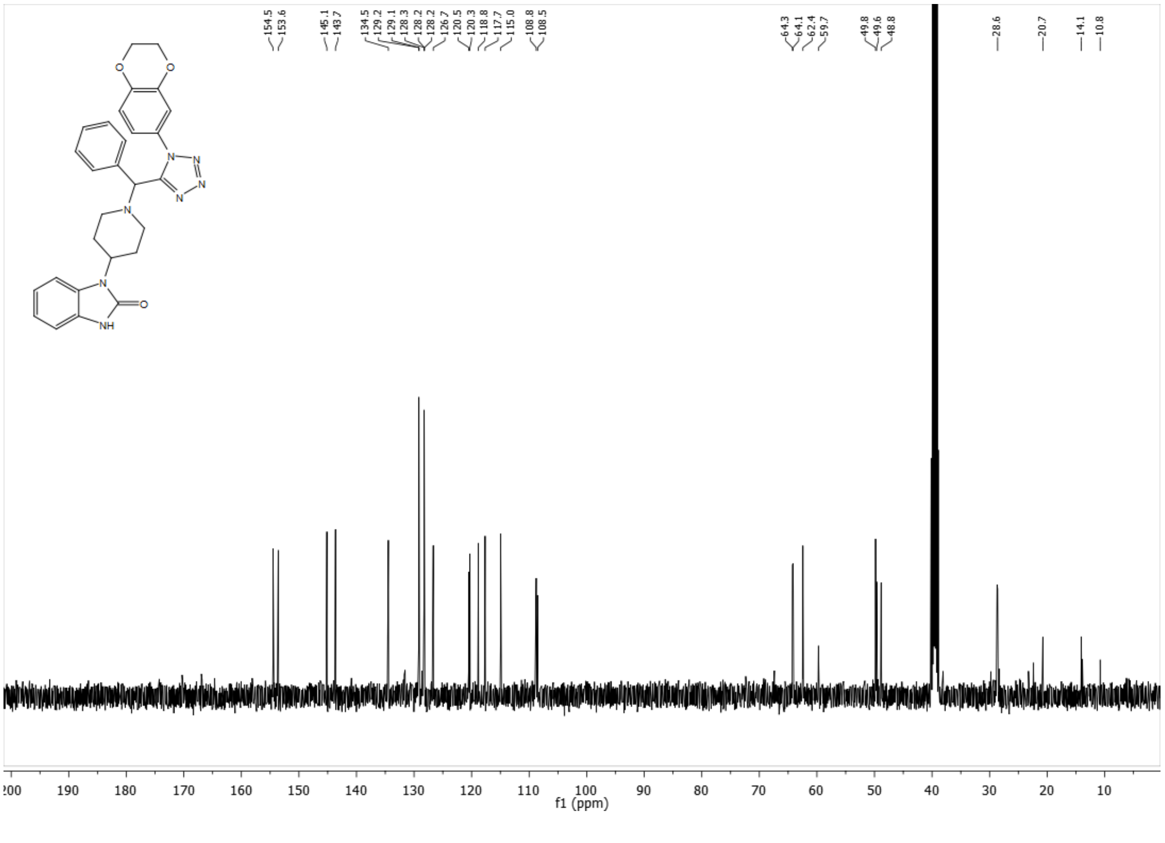

^{1}H NMR for **9j** ((DMSO-d_6)

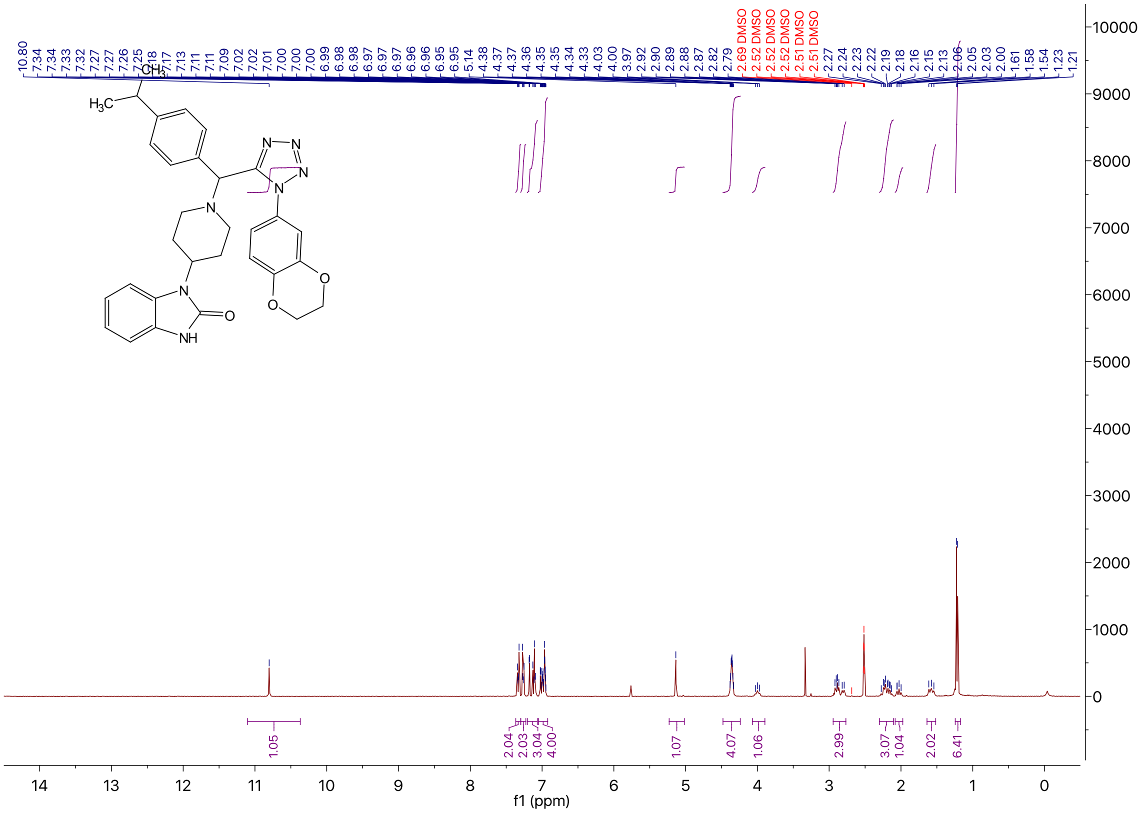

^{13}C NMR for **9j** (DMSO-d_6)

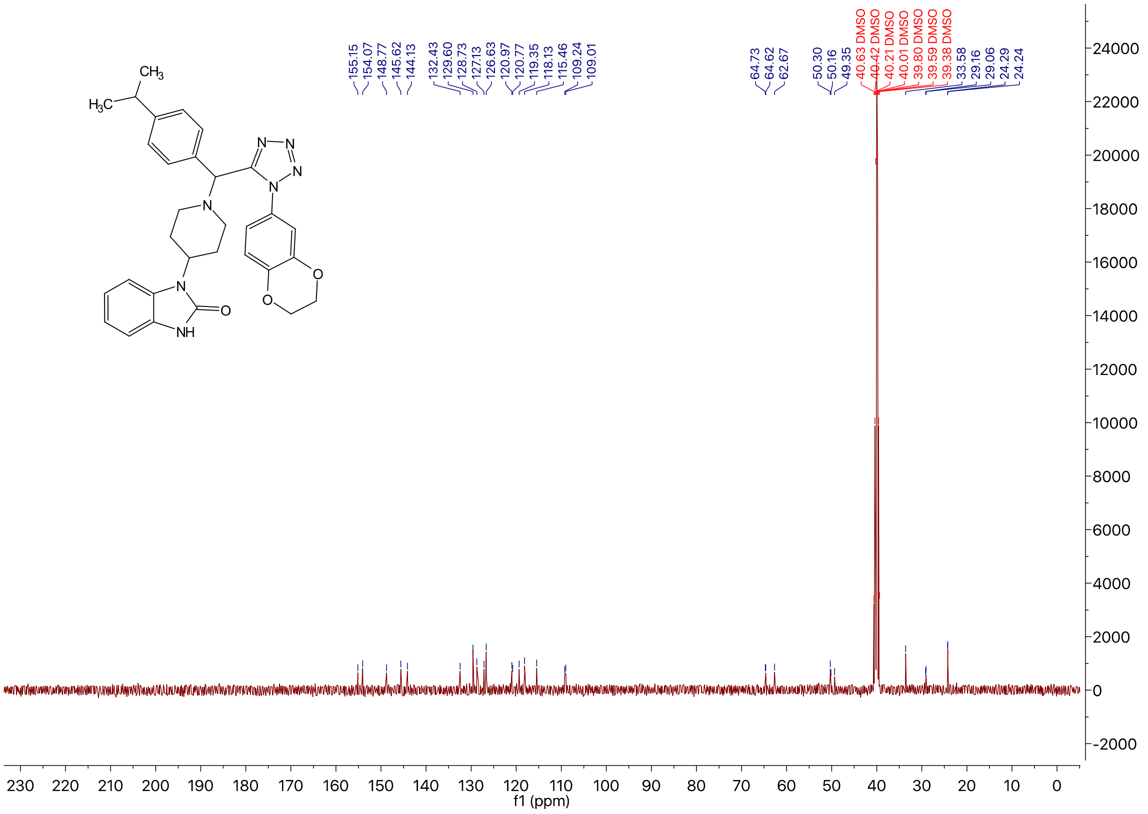

^{1}H NMR for **9k** ($CDCl_3$)

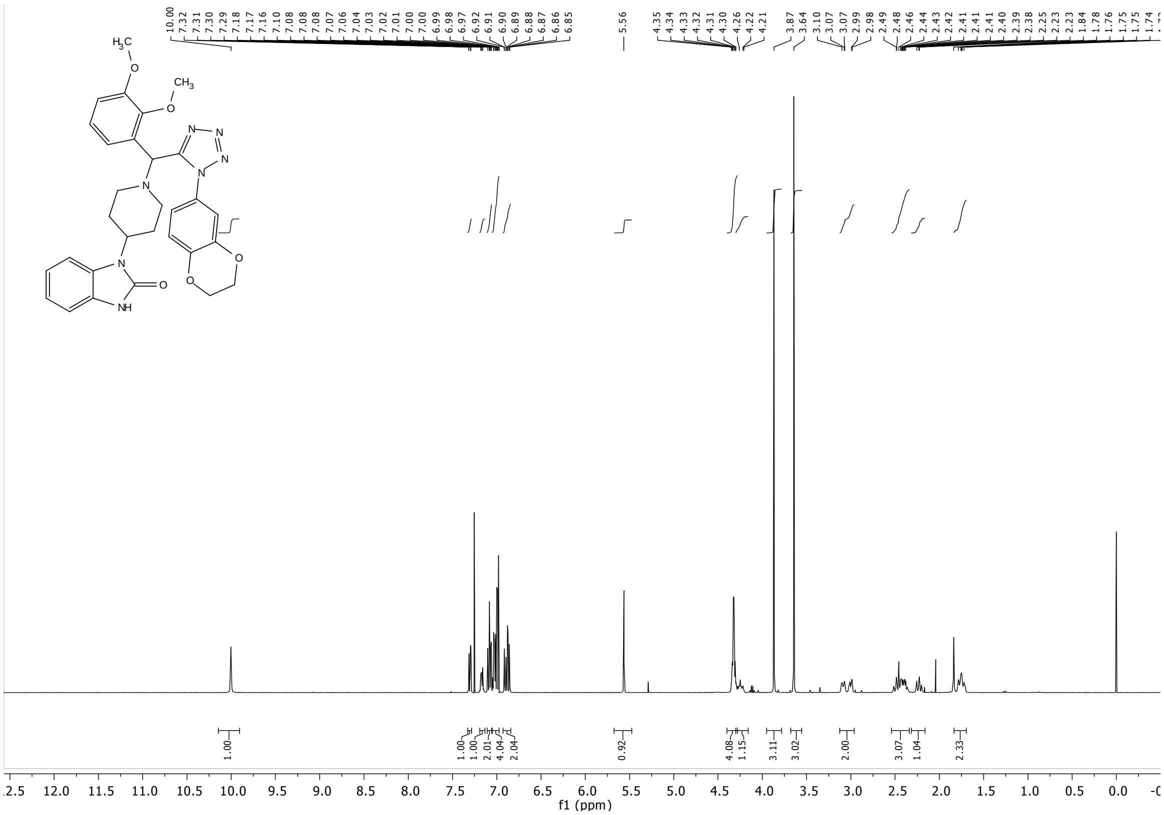

^{13}C NMR for **9k** ($CDCl_3$)

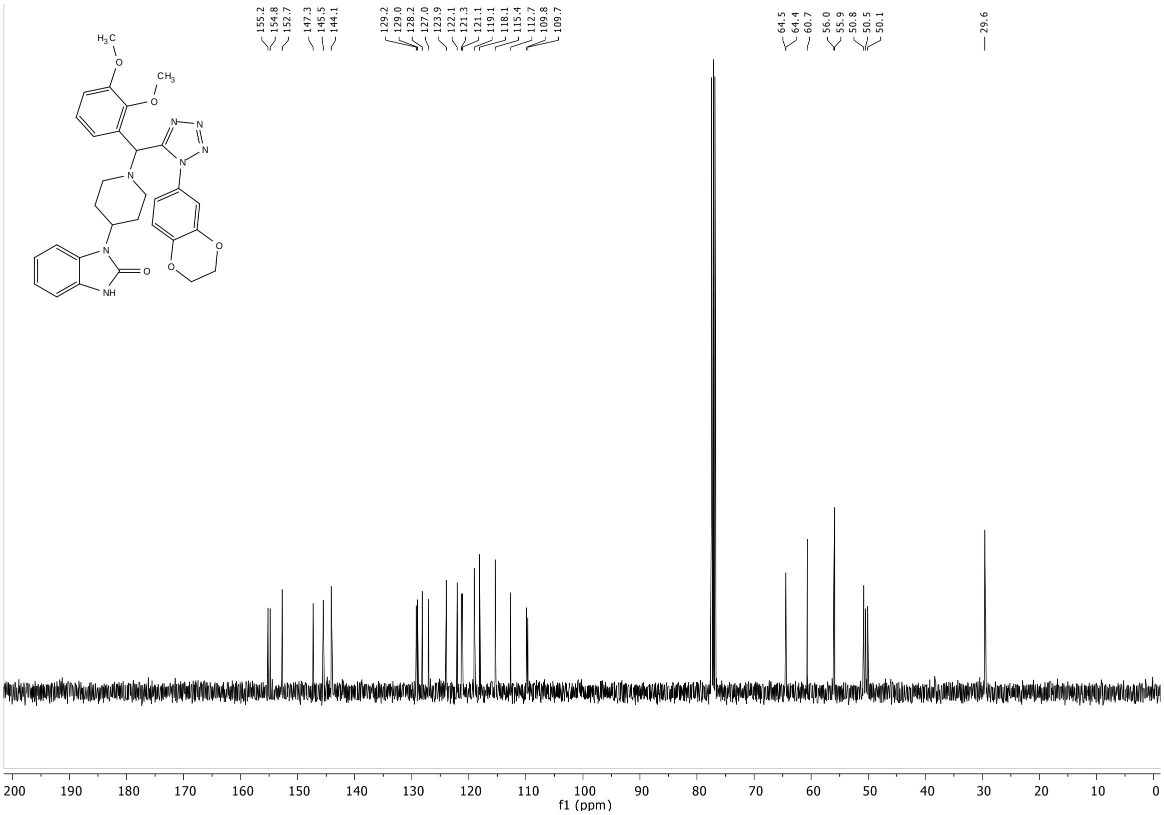

^{1}H NMR for **9l** ((DMSO-d_6)

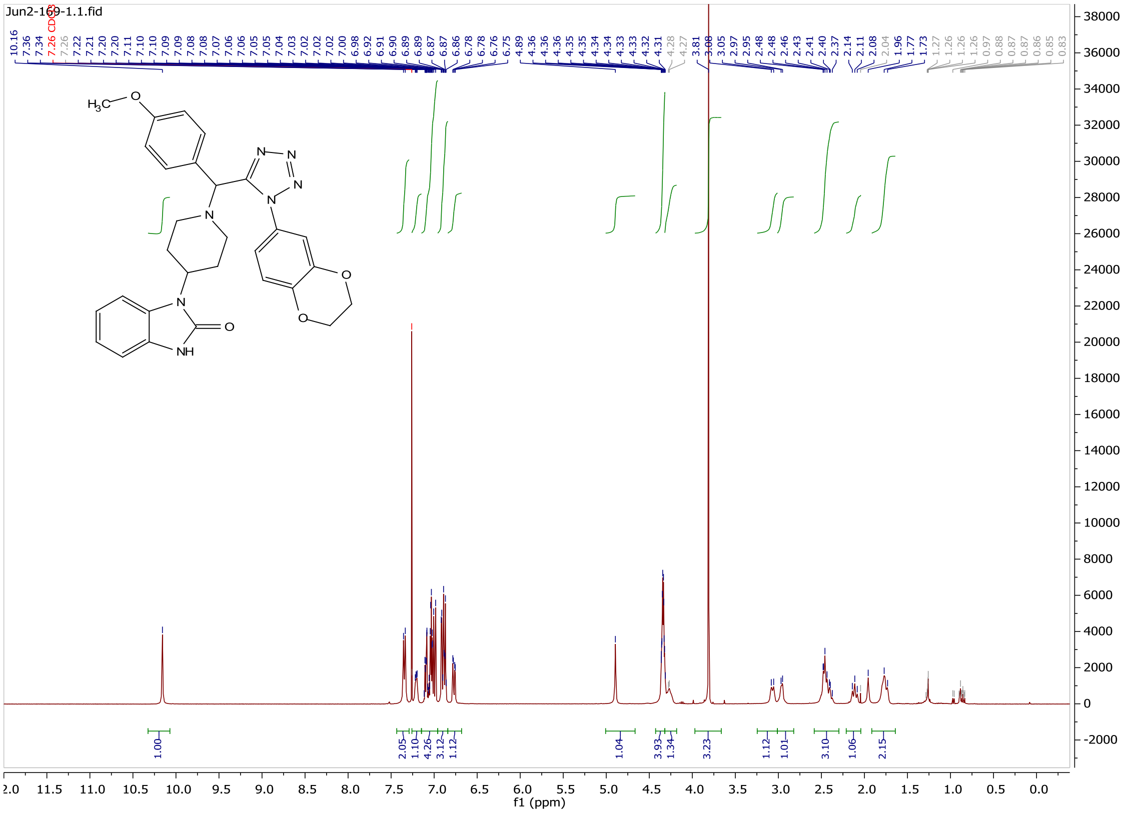

^{13}C NMR for **9l** (DMSO-d_6)

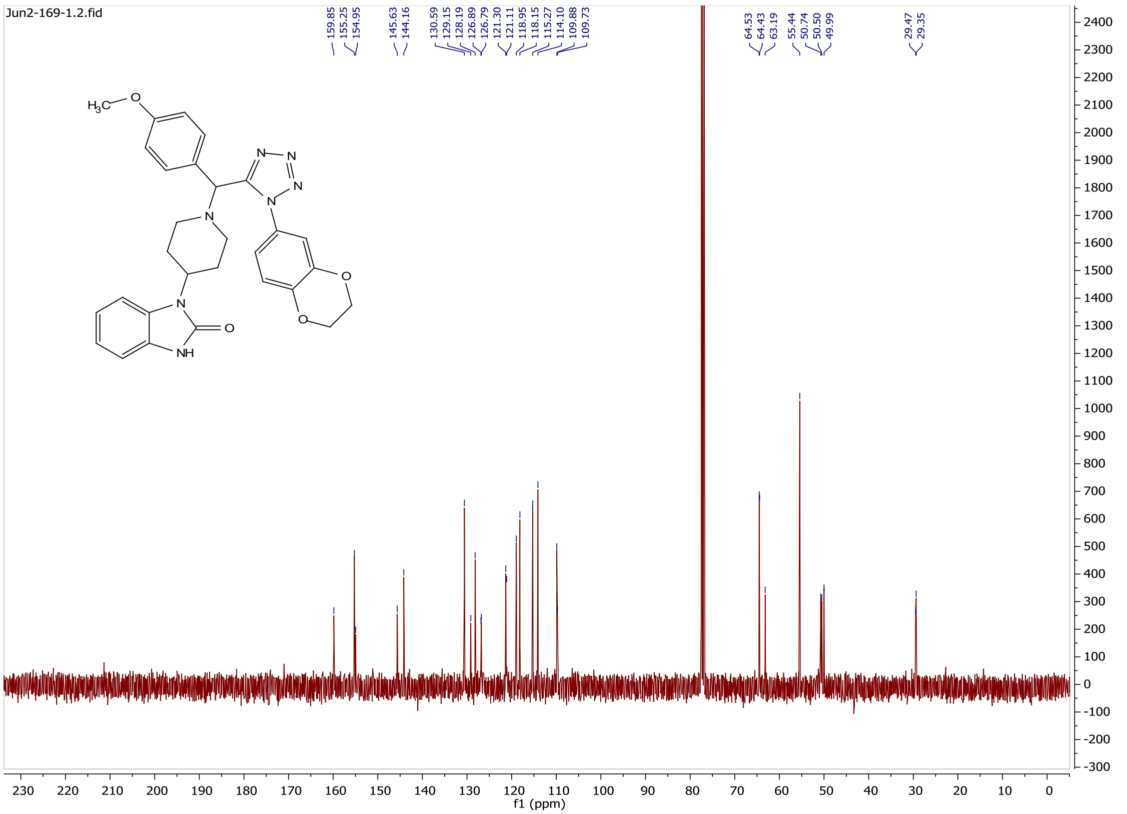

^{1}H NMR for **9m** ($CDCl_3$)

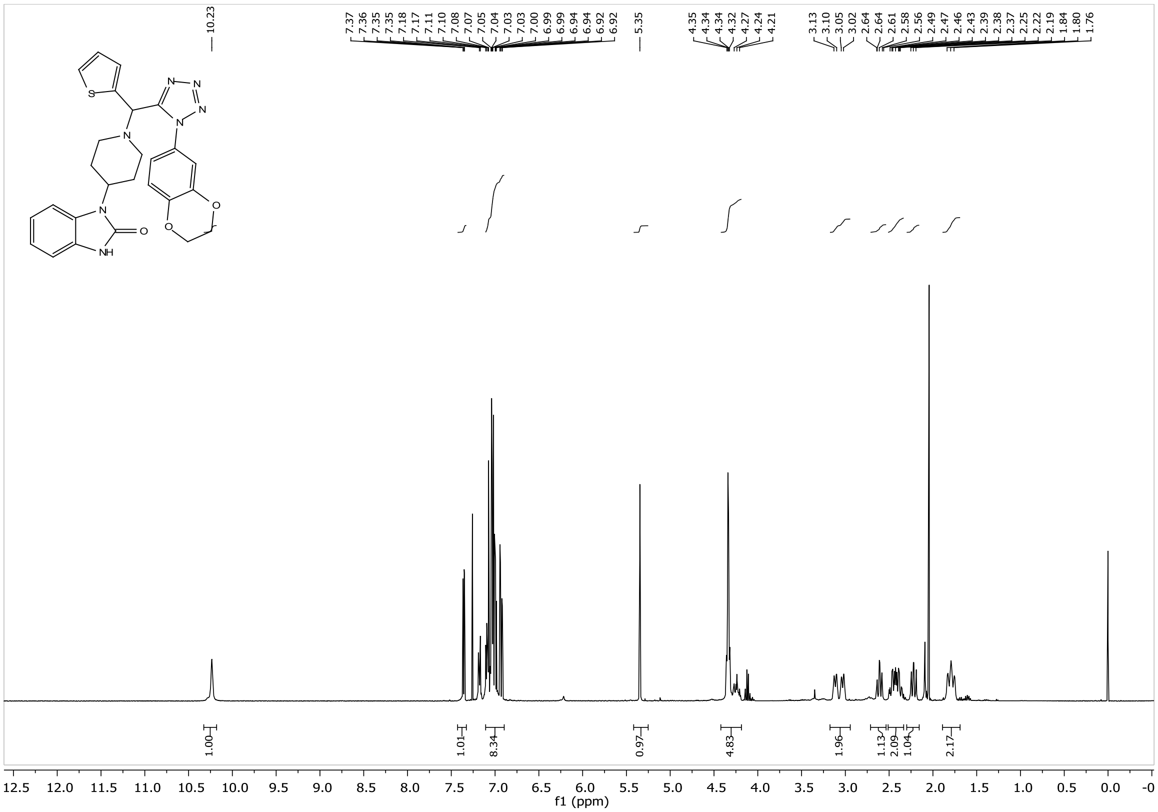

^{13}C NMR for **9m** ($CDCl_3$)

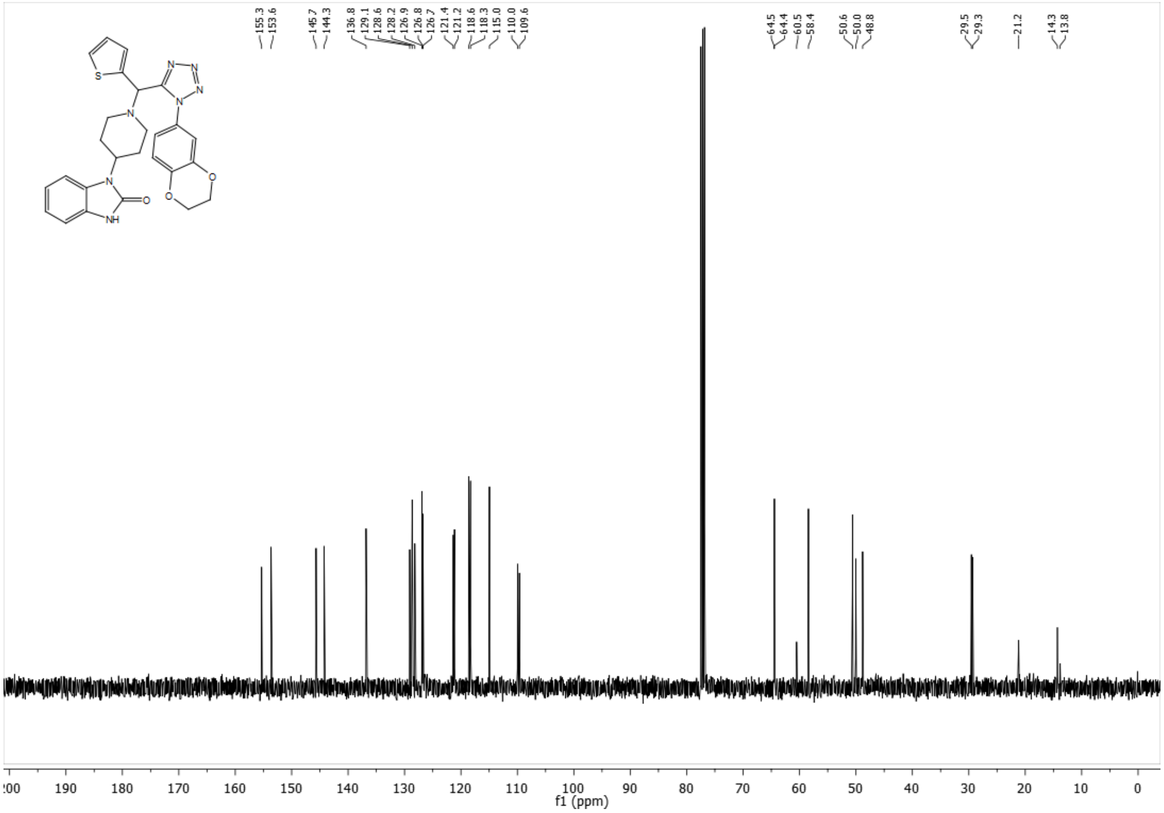

^{1}H NMR for **9n** ($CDCl_3$)

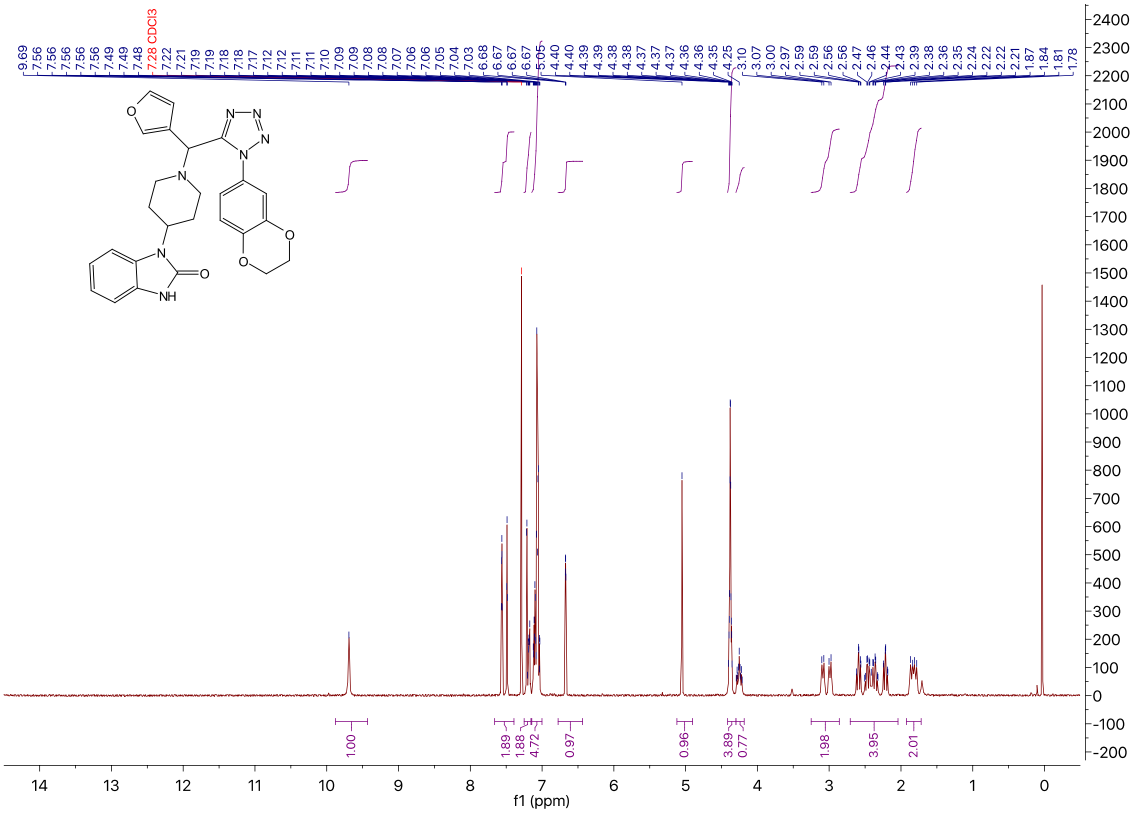

^{13}C NMR for **9n** ($CDCl_3$)

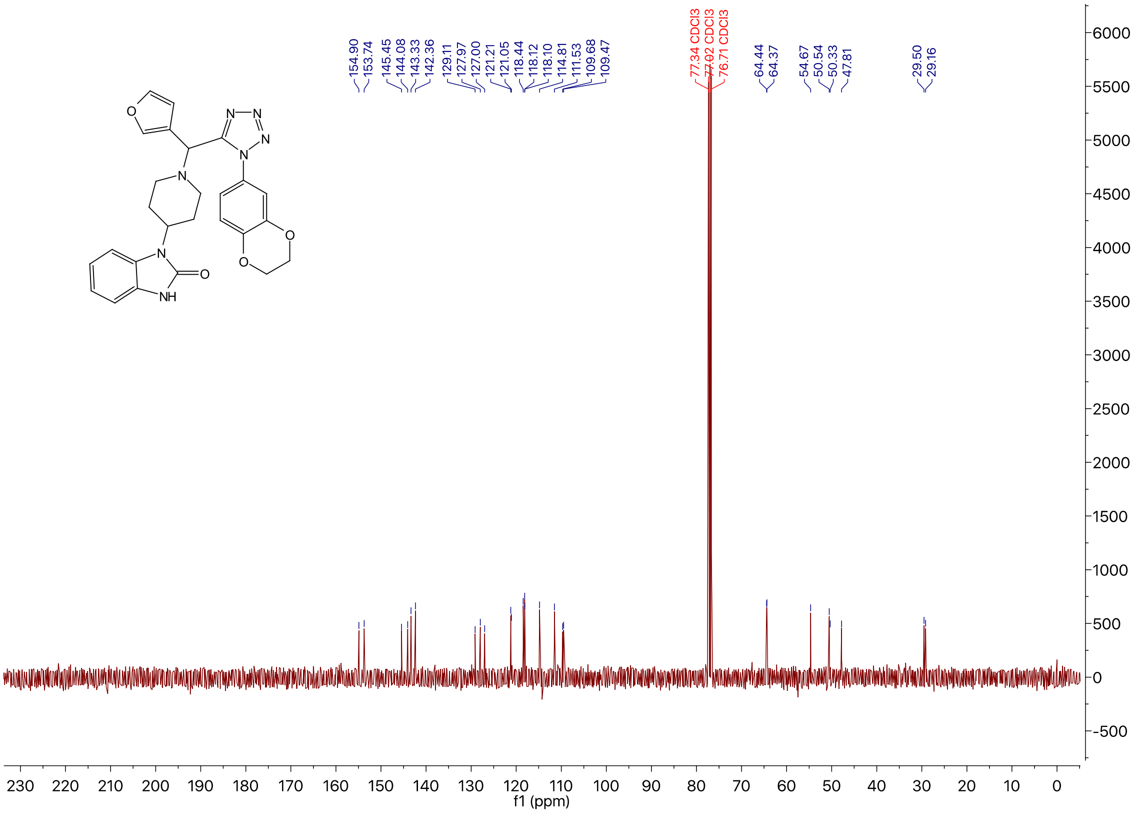

^{1}H NMR for **9o** ($CDCl_3$)

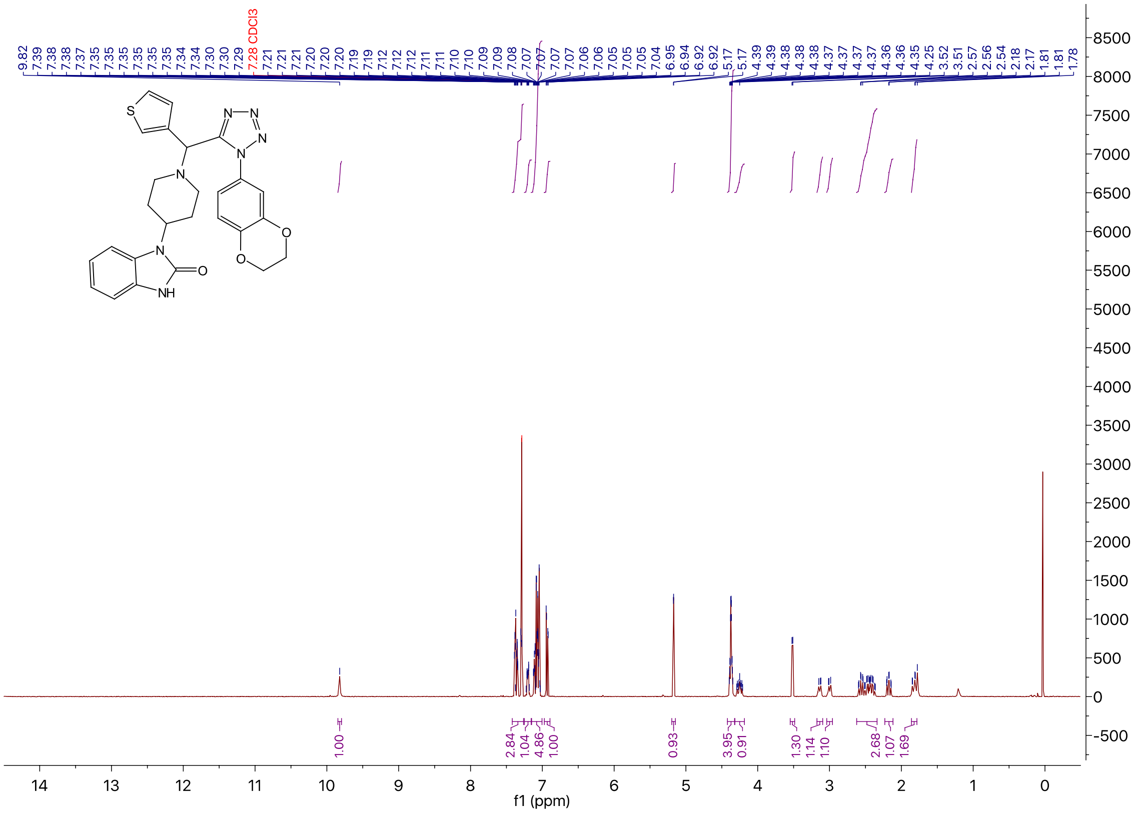

^{13}C NMR for **9o** ($CDCl_3$)

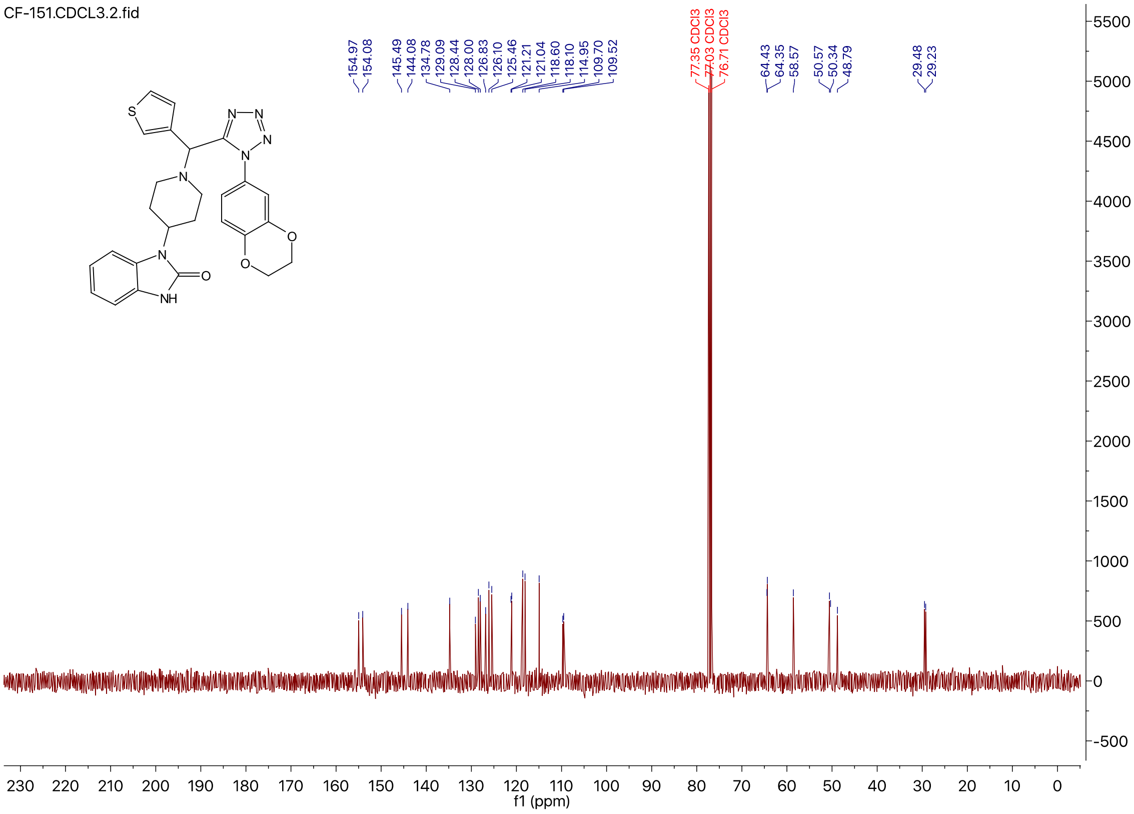

^{1}H NMR for **9p** ((DMSO-d_6)

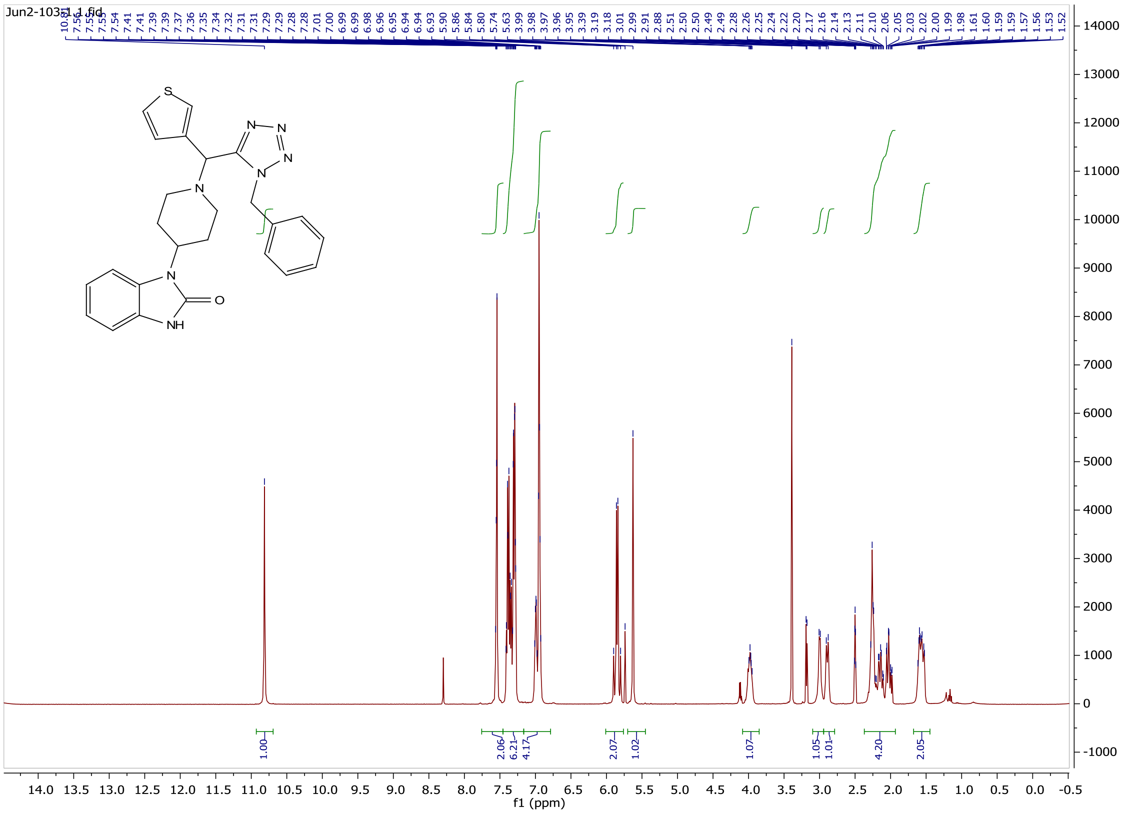

^{13}C NMR for **9p** (DMSO-d_6)

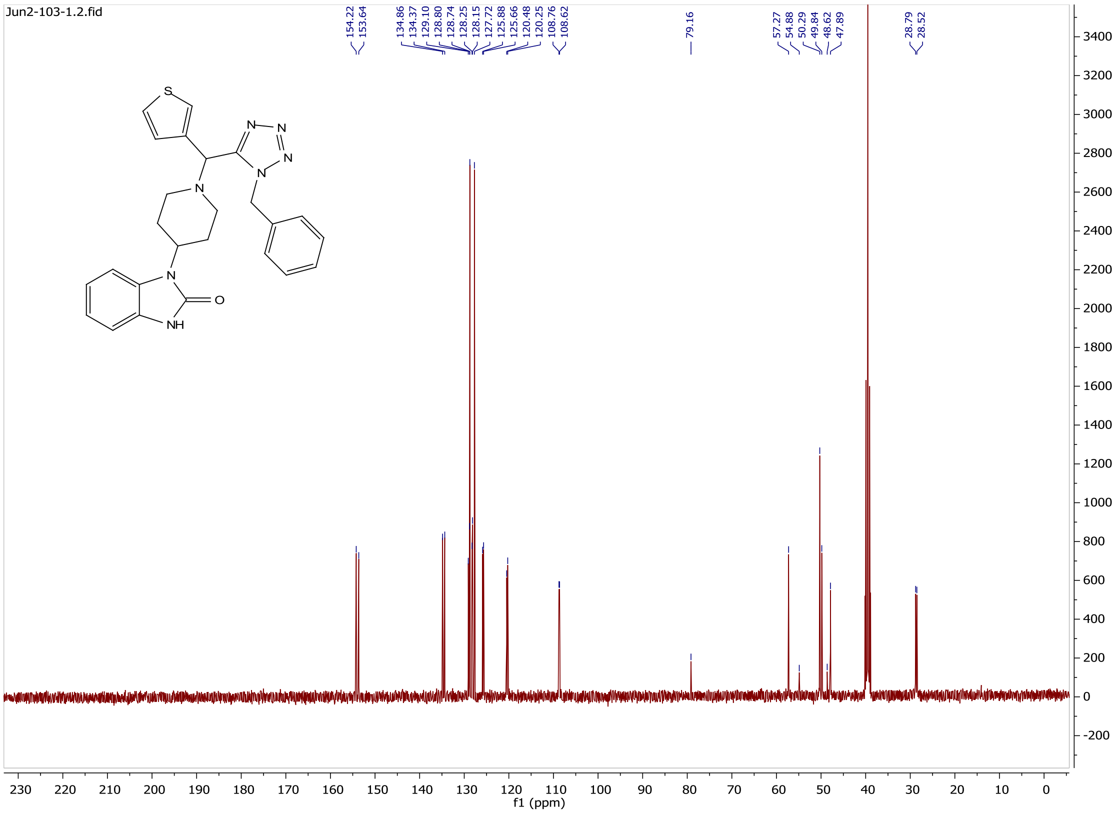

^{1}H NMR for **9q** ((DMSO-d_6)

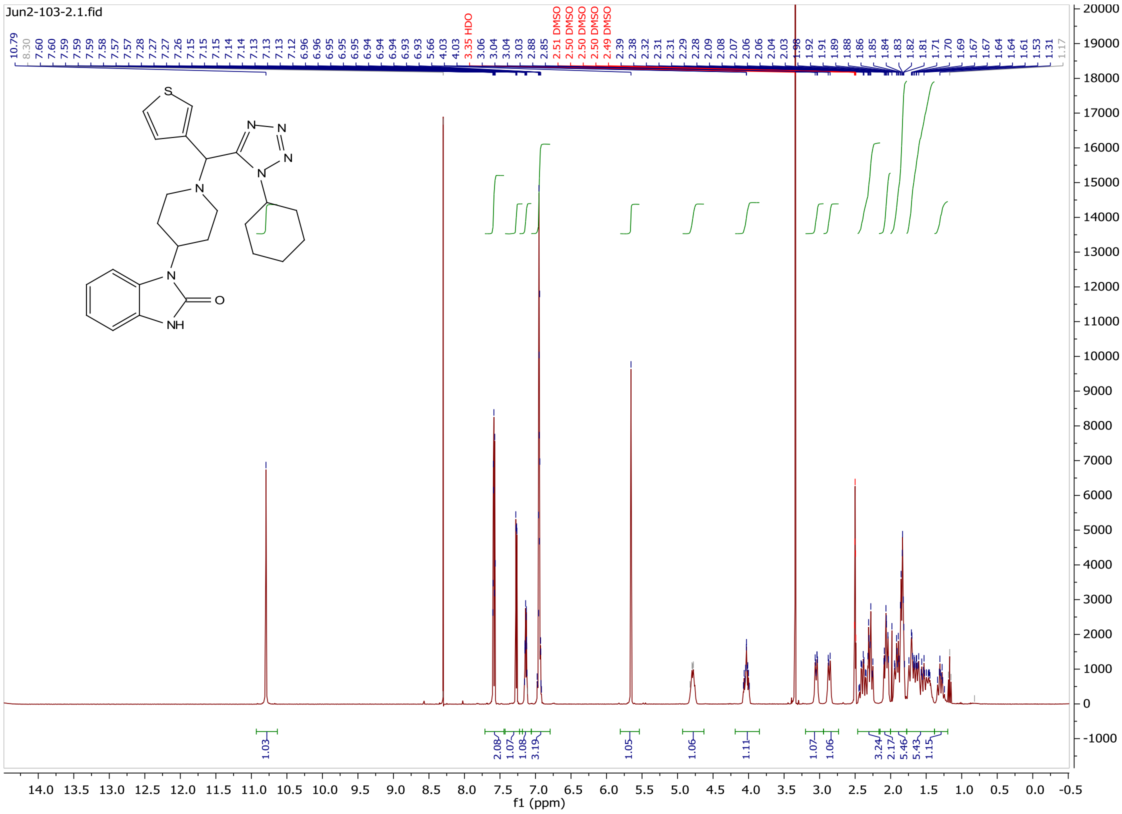

^{13}C NMR for **9q** (DMSO-d_6)

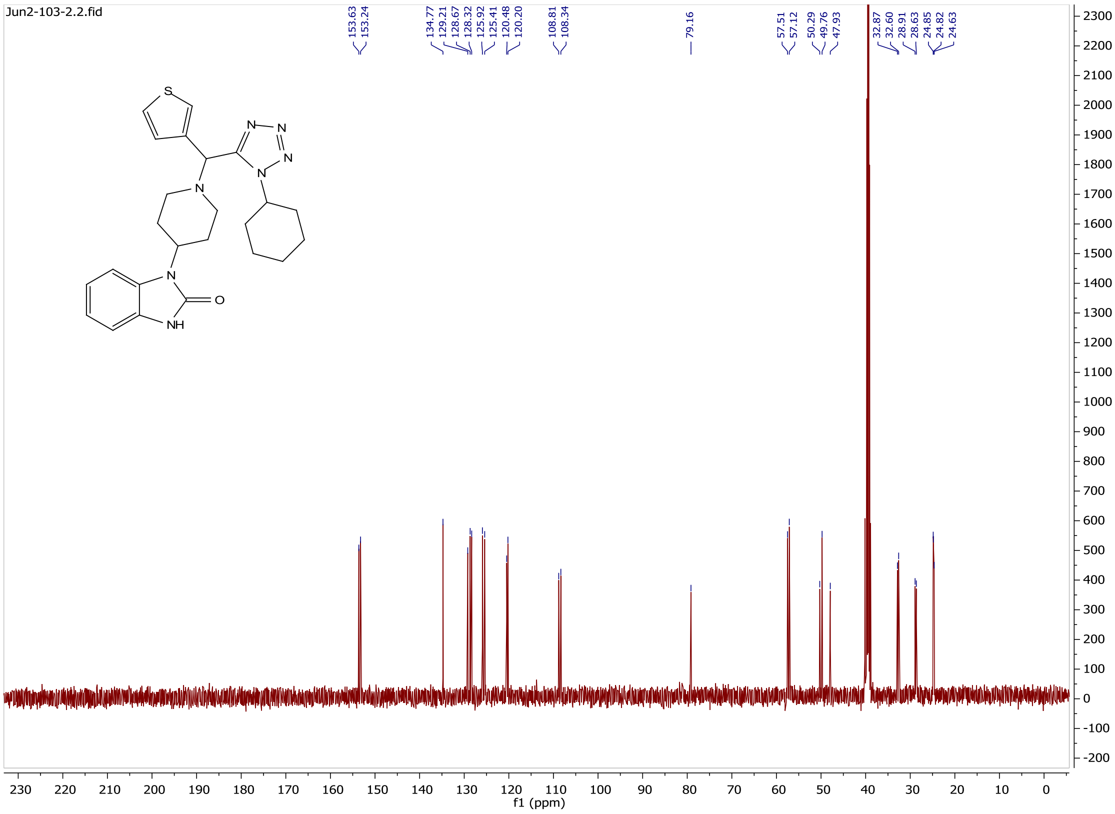

^{1}H NMR for **9r** ((DMSO-d$_6$)

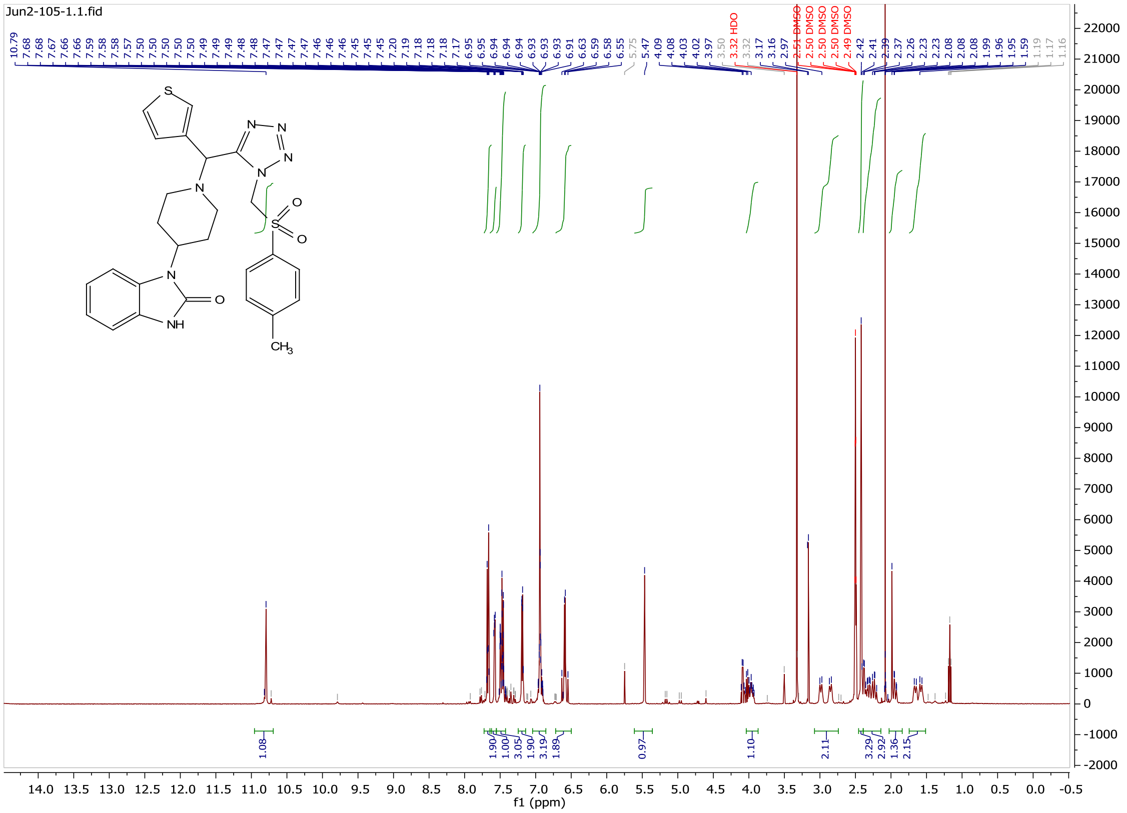

^{13}C NMR for **9r** (DMSO-d$_6$)

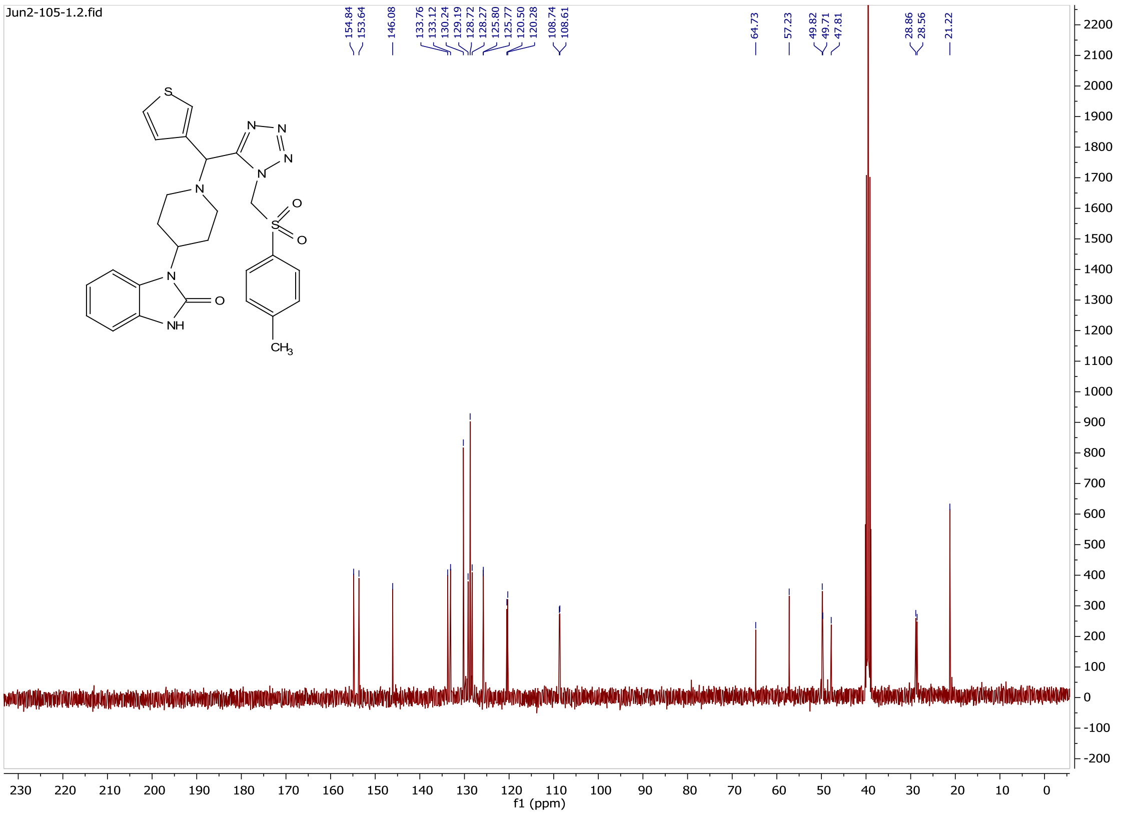

^{1}H NMR for **9s** ((DMSO-d$_6$)

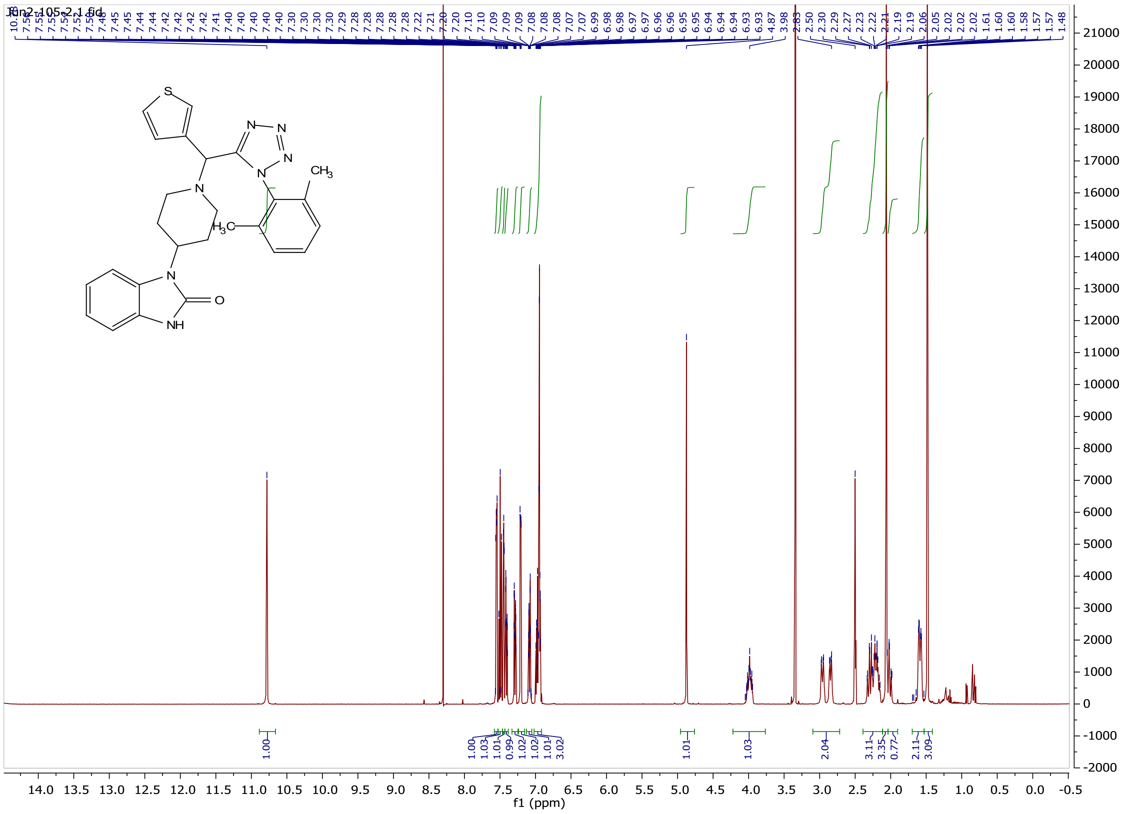

^{13}C NMR for **9s** (DMSO-d$_6$)

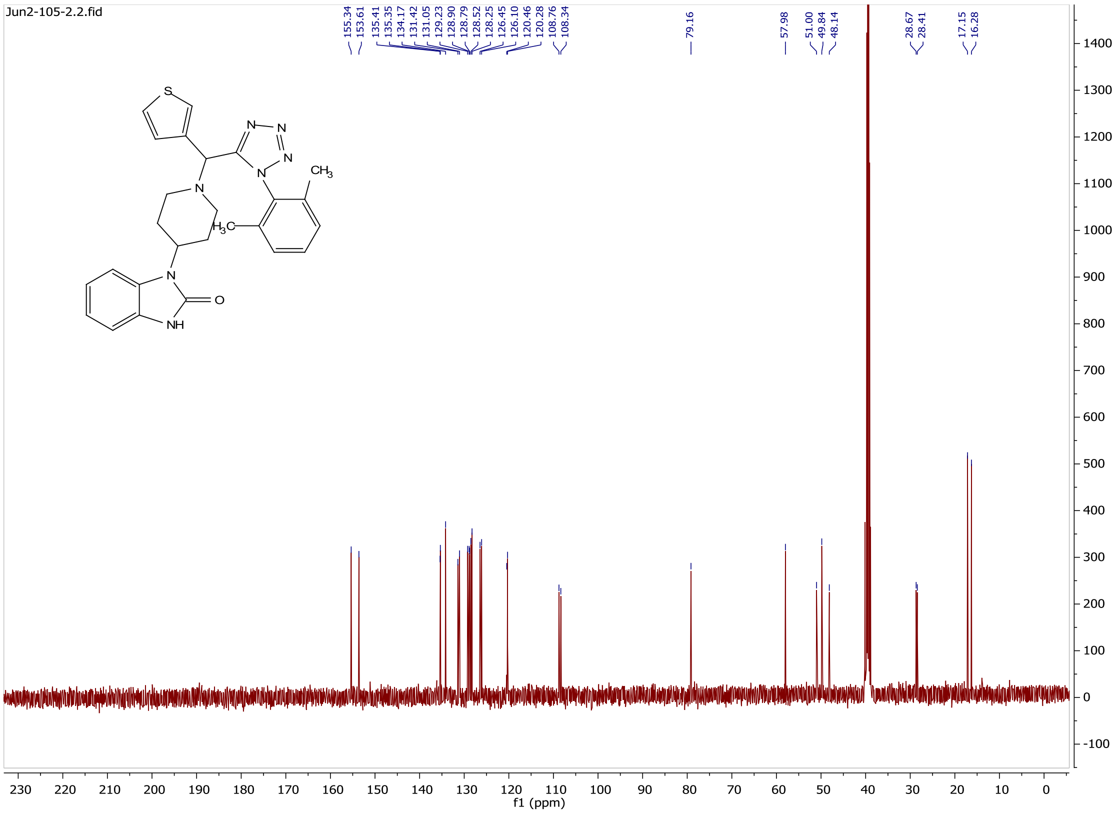

^{1}H NMR for **9t** ($CDCl_3$)

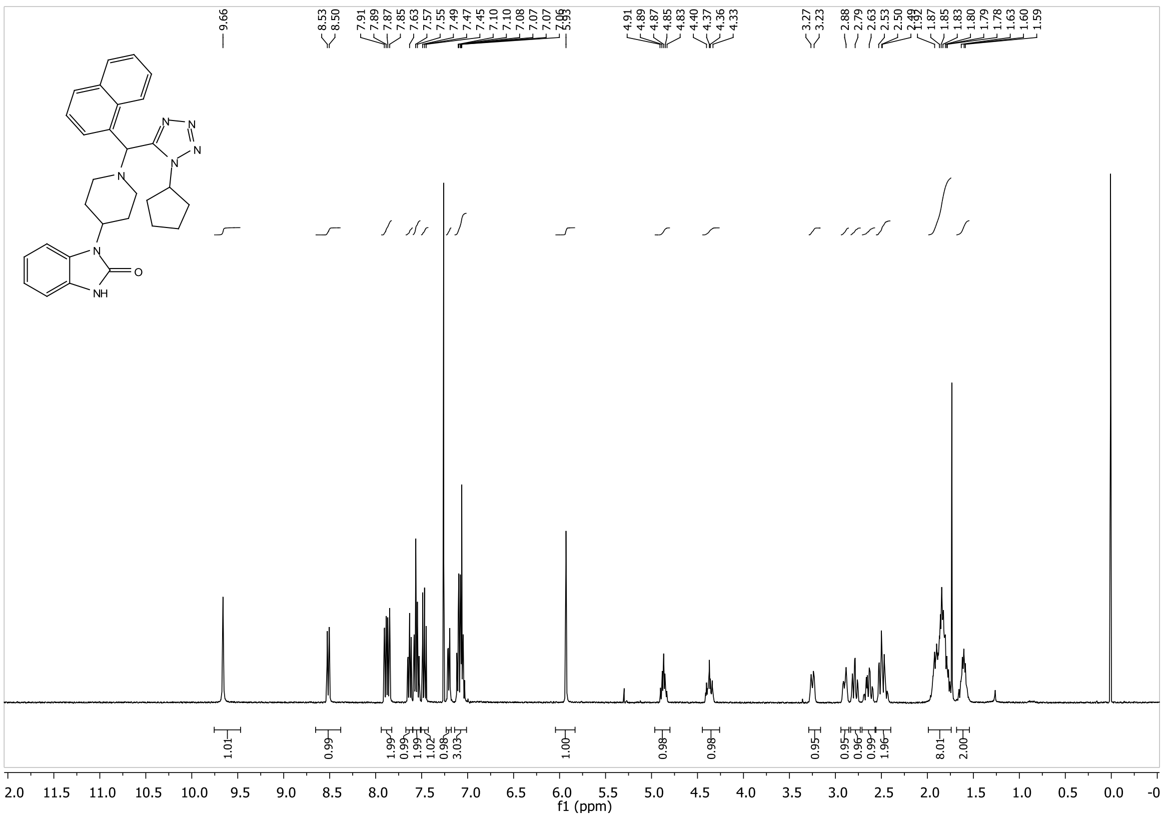

^{13}C NMR for **9t** ($CDCl_3$)

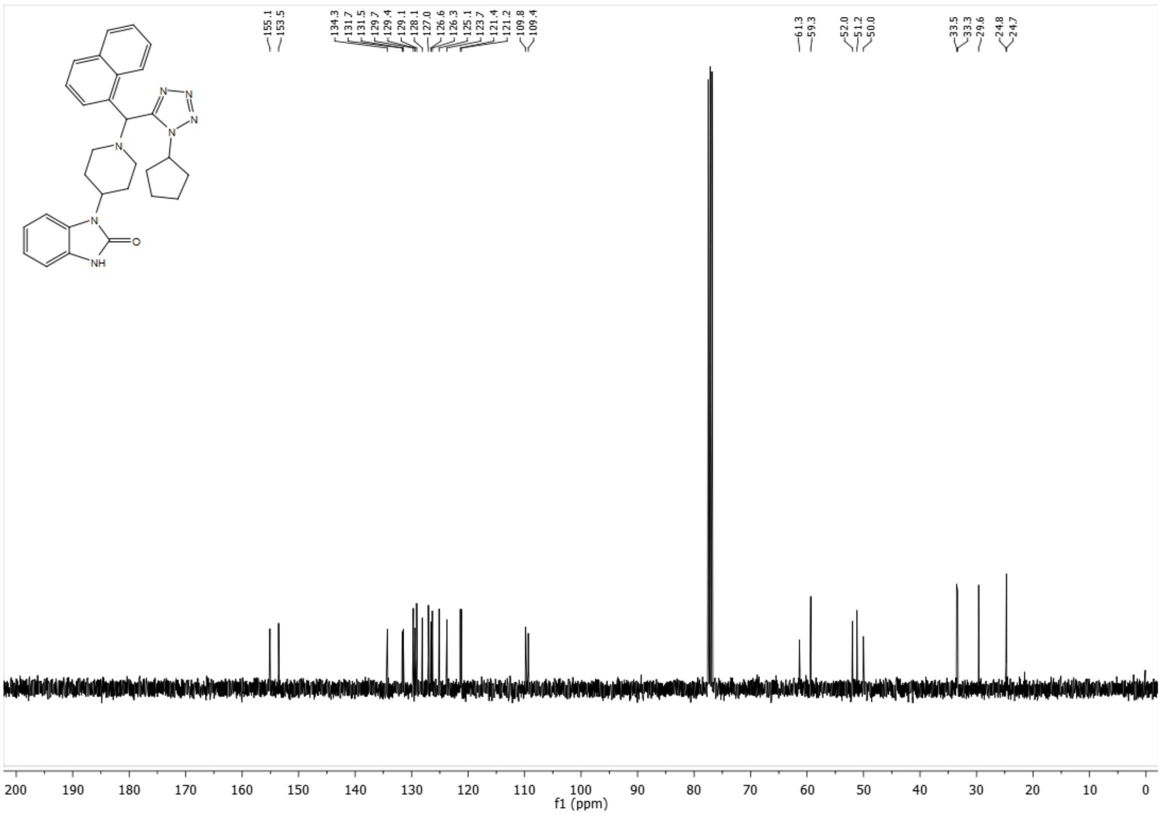

^{1}H NMR for **9u** ($CDCl_3$)

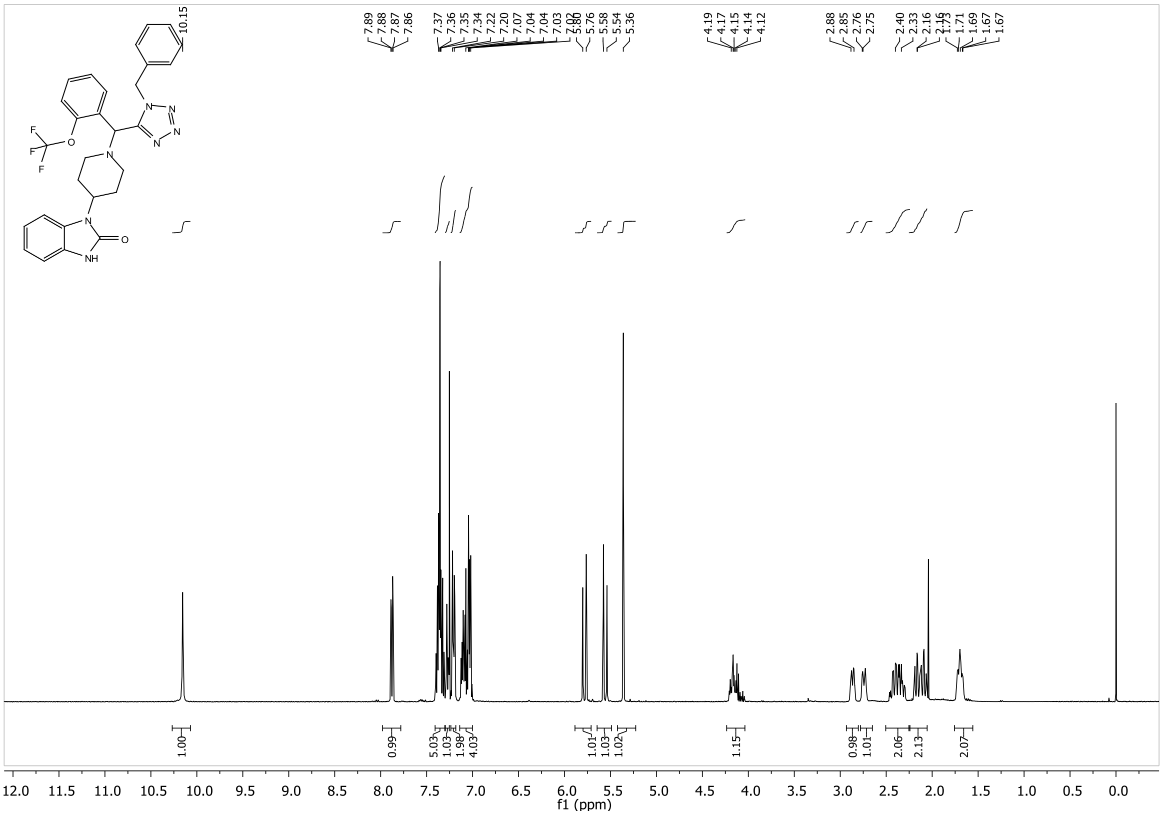

^{13}C NMR for **9u** ($CDCl_3$)

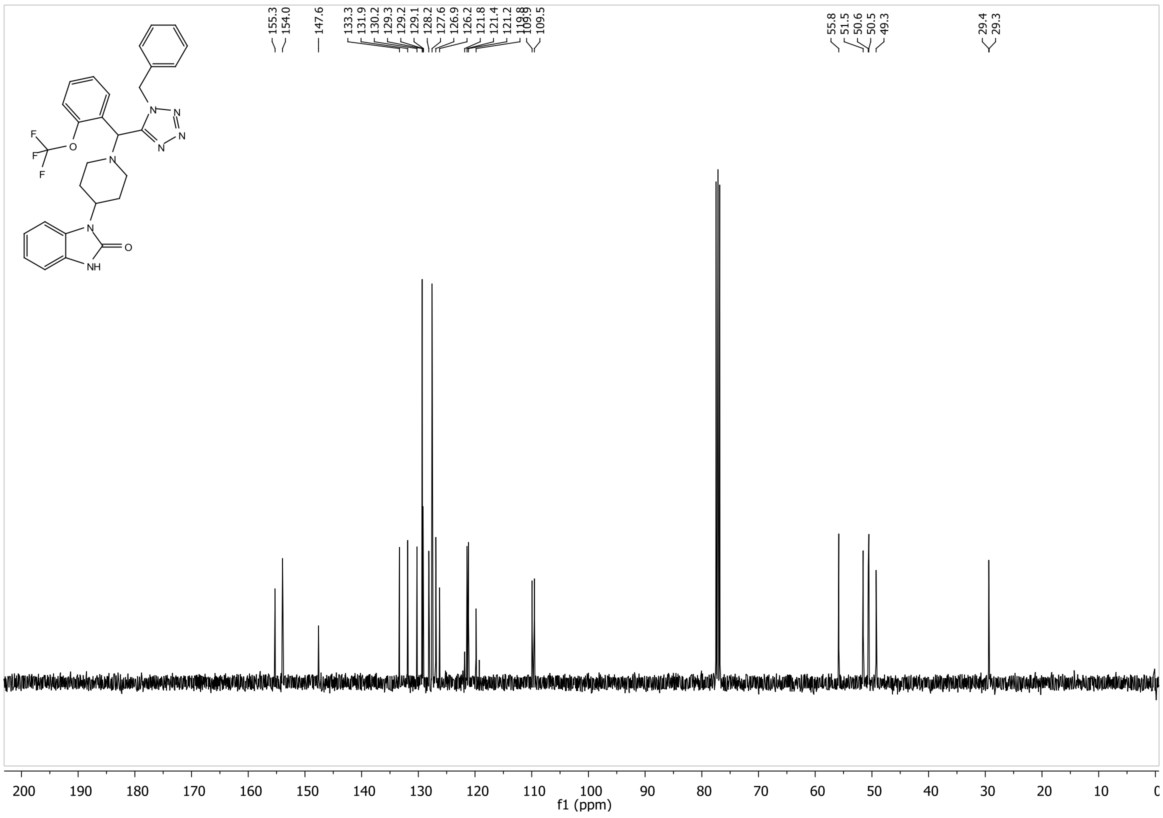

^{1}H NMR for **12a** ($CDCl_3$)

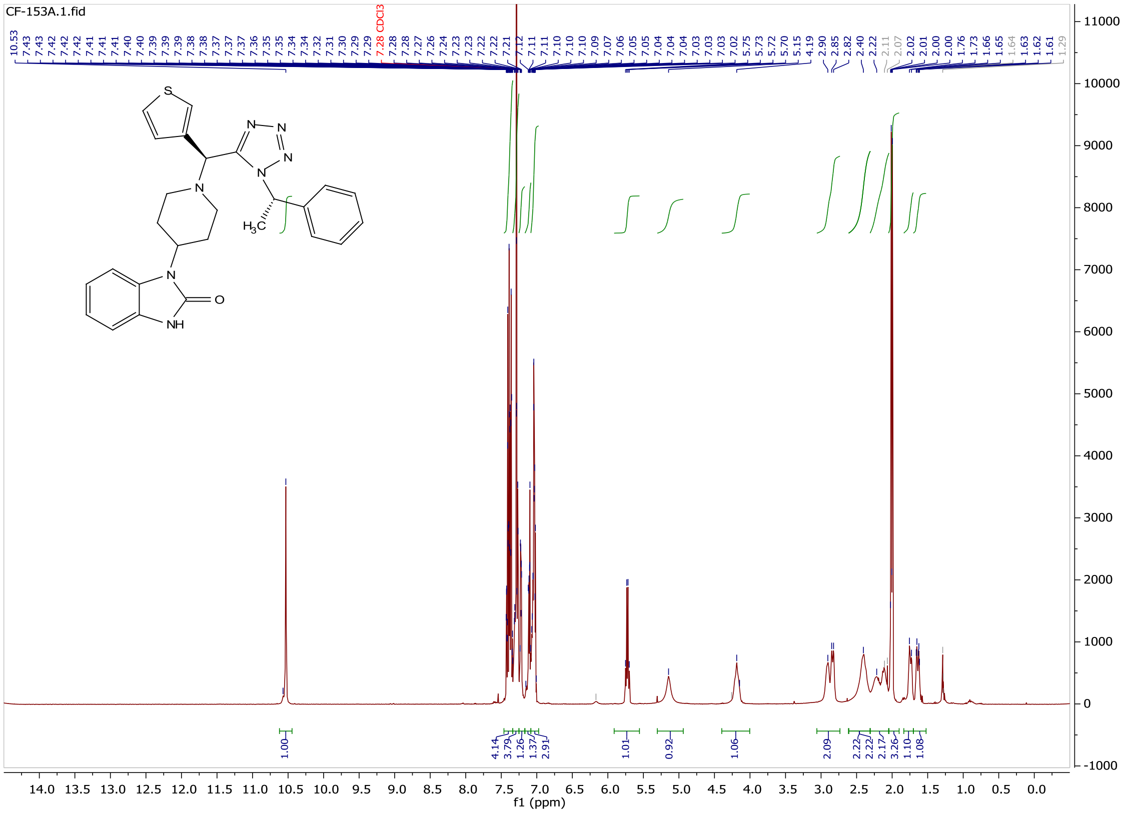

^{13}C NMR for **12a** ($CDCl_3$)

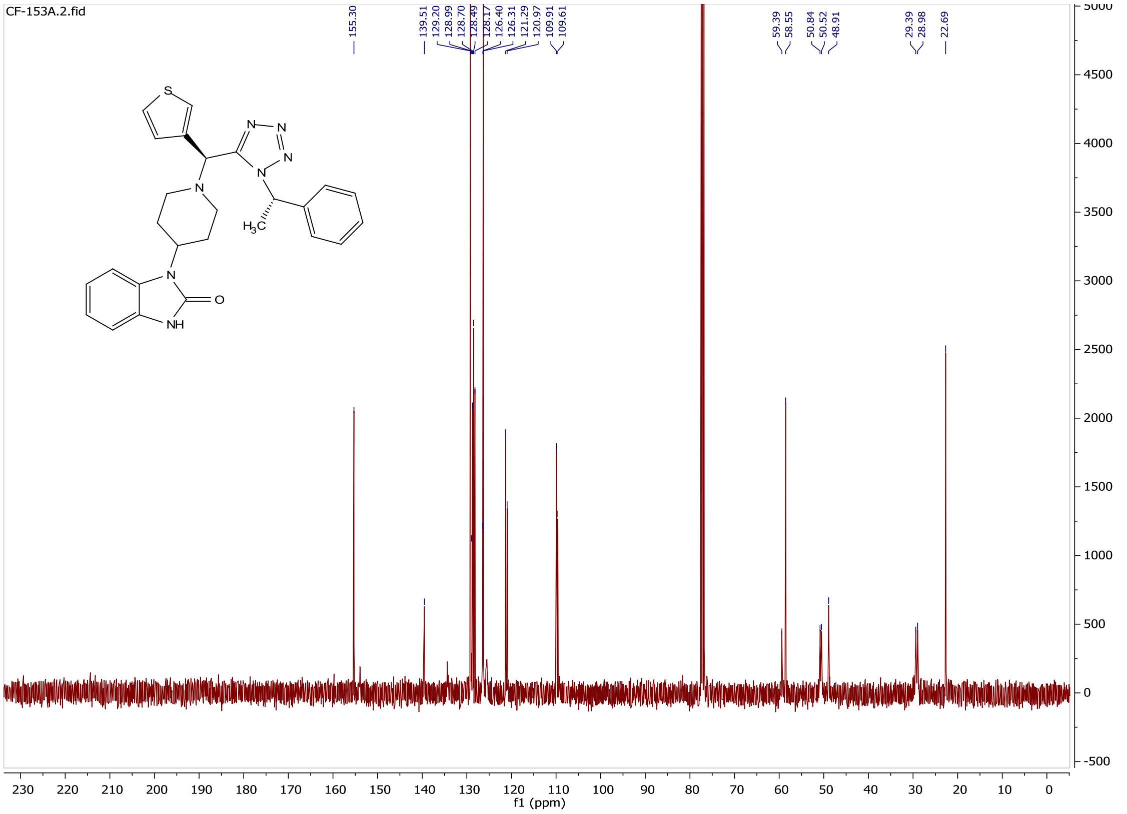

^{1}H NMR for **12b** ($CDCl_3$)

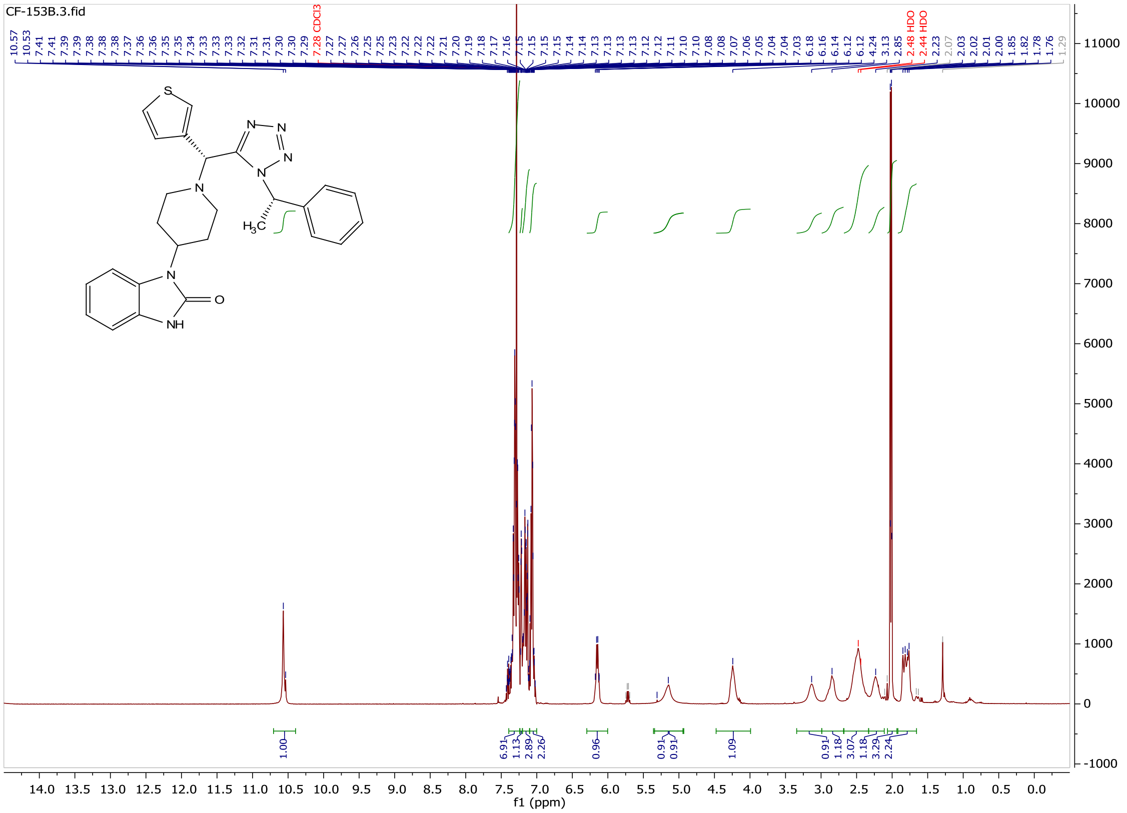

^{13}C NMR for **12b** ($CDCl_3$)

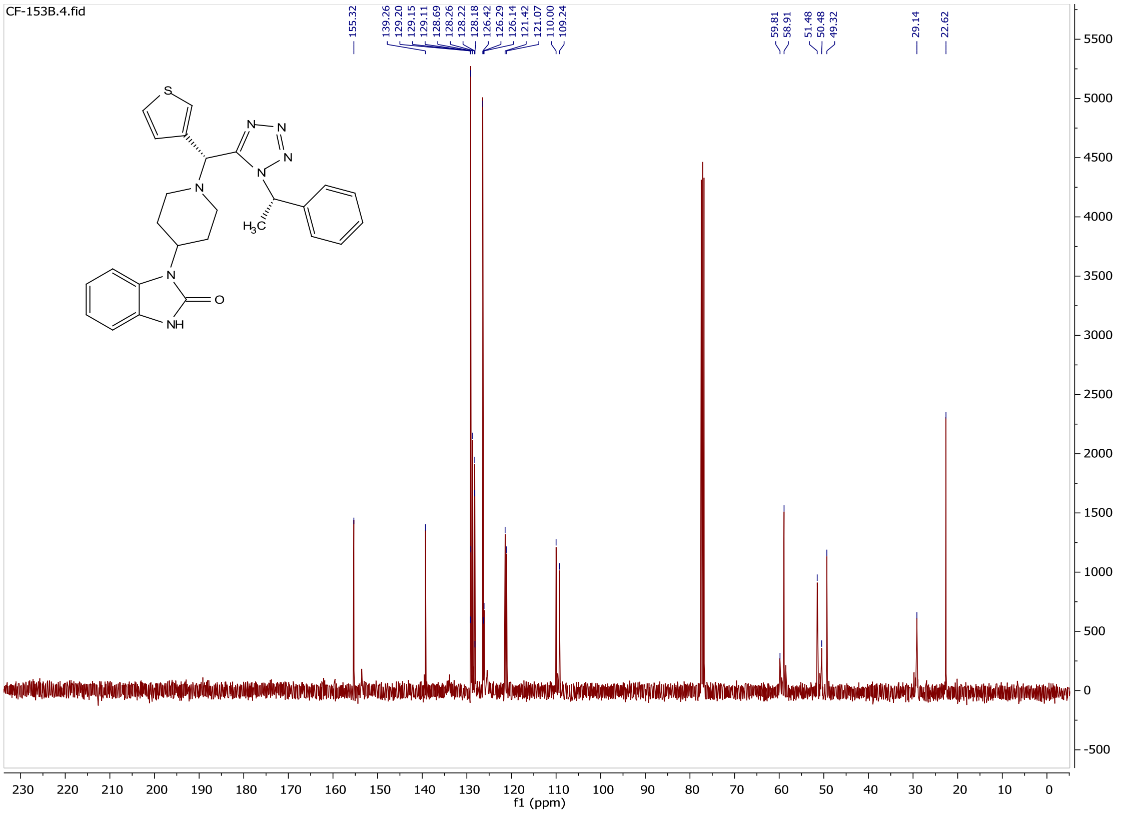

Supplement: Supplementary file 1 — Supplementary information [file 41598_2018_22875_MOESM1_ESM.pdf]
